# PhageGE: an interactive web platform for exploratory analysis and visualization of bacteriophage genomes

**DOI:** 10.1093/gigascience/giae074

**Published:** 2024-09-25

**Authors:** Jinxin Zhao, Jiru Han, Yu-Wei Lin, Yan Zhu, Michael Aichem, Dimitar Garkov, Phillip J Bergen, Sue C Nang, Jian-Zhong Ye, Tieli Zhou, Tony Velkov, Jiangning Song, Falk Schreiber, Jian Li

**Affiliations:** Infection Program and Department of Microbiology, Biomedicine Discovery Institute, Monash University, Clayton 3800, Australia; Monash Biomedicine Discovery Institute-Wenzhou Medical University Alliance in Clinical and Experimental Biomedicine, Monash University, Clayton 3800, Australia; Population Health and Immunity Division, The Walter and Eliza Hall Institute of Medical Research, Parkville 3052, Australia; Infection Program and Department of Microbiology, Biomedicine Discovery Institute, Monash University, Clayton 3800, Australia; Infection Program and Department of Microbiology, Biomedicine Discovery Institute, Monash University, Clayton 3800, Australia; Systems Biology Center, Tianjin Institute of Industrial Biotechnology, Chinese Academy of Sciences, Tianjin 300308, China; Department of Computer and Information Science, University of Konstanz, Konstanz 78457, Germany; Department of Computer and Information Science, University of Konstanz, Konstanz 78457, Germany; Infection Program and Department of Microbiology, Biomedicine Discovery Institute, Monash University, Clayton 3800, Australia; Infection Program and Department of Microbiology, Biomedicine Discovery Institute, Monash University, Clayton 3800, Australia; Key Laboratory of Clinical Laboratory Diagnosis and Translational Research of Zhejiang Province, Department of Clinical Laboratory, The First Affiliated Hospital of Wenzhou Medical University, Wenzhou 325015, China; Wenzhou Medical University–Monash Biomedicine Discovery Institute Alliance in Clinical and Experimental Biomedicine, The First Affiliated Hospital of Wenzhou Medical University,Wenzhou 325015, China; Key Laboratory of Clinical Laboratory Diagnosis and Translational Research of Zhejiang Province, Department of Clinical Laboratory, The First Affiliated Hospital of Wenzhou Medical University, Wenzhou 325015, China; Wenzhou Medical University–Monash Biomedicine Discovery Institute Alliance in Clinical and Experimental Biomedicine, The First Affiliated Hospital of Wenzhou Medical University,Wenzhou 325015, China; Department of Pharmacology, Biomedicine Discovery Institute, Monash University, Clayton 3800, Australia; Monash Biomedicine Discovery Institute-Wenzhou Medical University Alliance in Clinical and Experimental Biomedicine, Monash University, Clayton 3800, Australia; Department of Biochemistry and Molecular Biology, Biomedicine Discovery Institute, Monash University, Clayton 3800, Australia; Department of Computer and Information Science, University of Konstanz, Konstanz 78457, Germany; Faculty of Information Technology, Monash University, Clayton 3800, Australia; Infection Program and Department of Microbiology, Biomedicine Discovery Institute, Monash University, Clayton 3800, Australia; Monash Biomedicine Discovery Institute-Wenzhou Medical University Alliance in Clinical and Experimental Biomedicine, Monash University, Clayton 3800, Australia

**Keywords:** phage genome, biological web application, genomic analysis, phylogeny, lifestyle

## Abstract

**Background:**

Antimicrobial resistance is a serious threat to global health. Due to the stagnant antibiotic discovery pipeline, bacteriophages (phages) have been proposed as an alternative therapy for the treatment of infections caused by multidrug-resistant pathogens. Genomic features play an important role in phage pharmacology. However, our knowledge of phage genomics is sparse, and the use of existing bioinformatic pipelines and tools requires considerable bioinformatic expertise. These challenges have substantially limited the clinical translation of phage therapy.

**Findings:**

We have developed PhageGE (Phage Genome Explorer), a user-friendly graphical interface application for the interactive analysis of phage genomes. PhageGE enables users to perform key analyses, including phylogenetic analysis, visualization of phylogenetic trees, prediction of phage life cycle, and comparative analysis of phage genome annotations. The new R Shiny web server, PhageGE, integrates existing R packages and combines them with several newly developed functions to facilitate these analyses. Additionally, the web server provides interactive visualization capabilities and allows users to directly export publication-quality images.

**Conclusions:**

PhageGE is a valuable tool that simplifies the analysis of phage genome data and may expedite the development and clinical translation of phage therapy. PhageGE is publicly available at https://jason-zhao.shinyapps.io/PhageGE_Update/.

## Introduction

The rapid emergence and spread of antimicrobial resistance (AMR) is one of the 3 greatest threats to human health globally [1]. It is estimated that by 2050, life-threatening infections caused by antimicrobial-resistant pathogens will kill more people than any other diseases [2]. Of particular concern is the increased prevalence of infections caused by Gram-negative pathogens, which are more difficult to treat than Gram-positive pathogens [3]. Given the sluggish global antibiotic pipeline [4], bacteriophages (phages) have attracted significant attention over the past decade as a potential alternative therapy for bacterial infections [5]. Phages are bacterial viruses and the advantages of phage therapy over antibiotics include a narrow spectrum of activity, the capacity to multiply at the infection site, and safety [6–8]. Optimizing phage therapy in patients requires key pharmacological information, including infection cycle, gene content, and phage taxonomy [9, 10]. For example, temperate phages do not immediately lyse bacterial host cells and have an inherent capacity to mediate the transfer of genes between bacteria, potentially facilitating increased bacterial virulence and AMR. In contrast, lytic phages kill bacteria upon infection and are commonly used for the treatment of multidrug-resistant (MDR) bacterial infections in patients [11–14].

Multiomics has the potential to expedite the clinical translation of phage therapy for the treatment of MDR bacterial infections [15]. For example, whole genome–based phylogenetic analysis offers significant advantages in understanding phage evolutionary dynamics and designing potential phage cocktails [16, 17]. Furthermore, combining whole-genome sequencing (WGS) with *in silico* prediction enables rapid prediction of phage lifestyle [18]. Several popular bioinformatic pipelines and tools are available for multiple sequence alignment (MAFFT) [19], phylogenetic reconstruction (RAxML and IQ-TREE) [20, 21], visualization of phylogeny (ggtree) [22], and phage lifestyle prediction (PHACTS and BACPHLIP) [18, 23]; however, utilizing these tools requires proficient programming skills. Therefore, a user-friendly platform for phage genomic analyses is urgently needed to overcome the challenges associated with the requirement for advanced programming expertise.

Here, we developed an integrated web server platform, PhageGE, that offers 4 key functionalities: phage phylogenetic analysis, tree visualization, lifestyle prediction, and manipulation of phage genome annotation datasets. PhageGE differs from existing phage genomic analysis tools in that it facilitates the seamless export of all associated results in a publication-ready format without requiring complex procedures or long running times. Overall, PhageGE provides a user-friendly interface to streamline phage genomic analysis with WGS data.

## Results

The PhageGE web server (biotoolsID: biotools:phagege and RRID:SCR_025380) was designed to ensure user-friendliness and compatibility with major web browsers, including Google Chrome, Mozilla Firefox, Apple Safari, and Microsoft Edge (Table 1).

**Table 1:** Browsers and operating systems (OS) tested with PhageGE

OS	Chrome	Edge	Firefox	Safari
**Linux**	120.0	120.0	121.0	n/a
**MacOS**	107.0	108.0	107.0.1	15.6.1
**Windows**	105.0	108.0	107.0.1	n/a

n/a, not applicable.

### Web server submission and case studies

**Figure 1:**
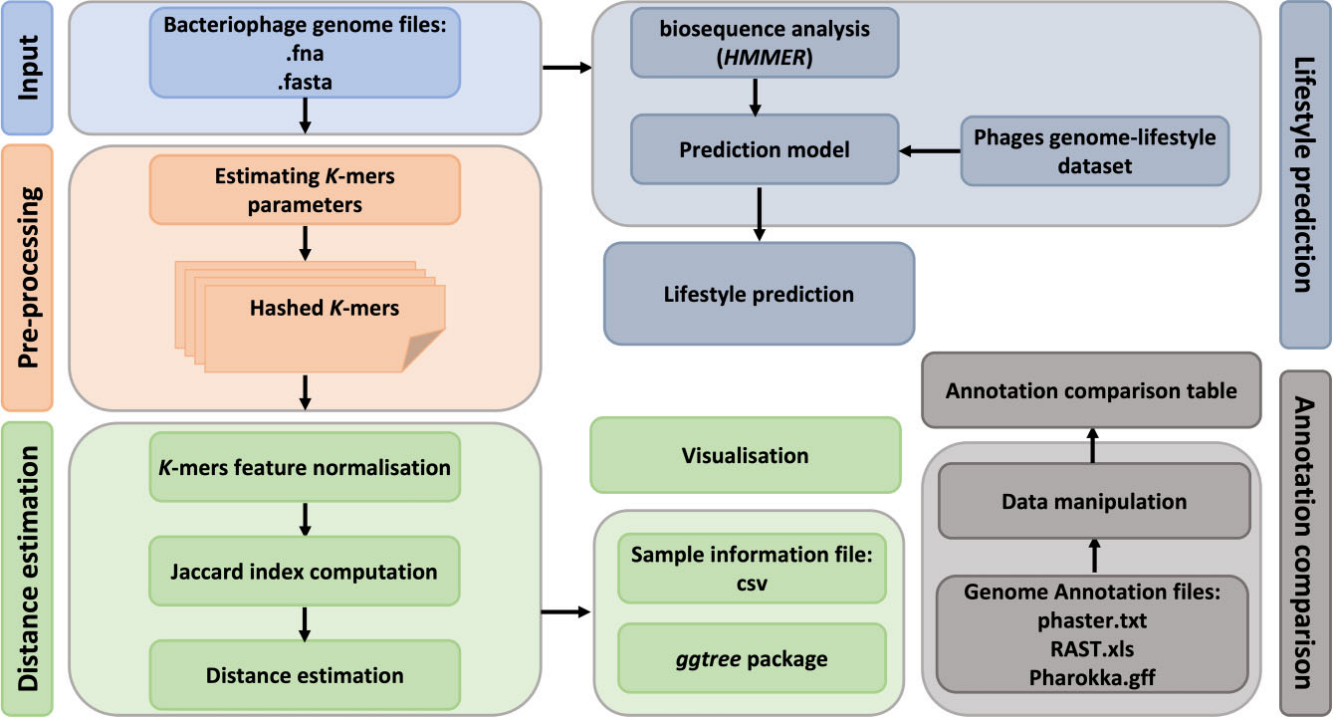
The workflow and application of PhageGE. Illustration of the workflow of PhageGE, highlighting its components and processes for phage genomic analysis. (1) Phylogenetic analysis. Input: Phage genome files in fasta format are uploaded. Preprocessing: The uploaded genome files are processed to estimate *k*-mer parameters and the *k*-mer are hashed for further analysis. Distance estimation: *k*-mers features are normolized and then used for Jaccard index computation. Distance estimation: Distances are estimated based on the computed Jaccard index. (2) Visualization. The results are visualized using the ggtree package and sample information files in CSV format. (3) Lifestyle prediction. Biosequence analysis (HMMER): Biosequence analysis is performed using HMMER. Prediction model: A prediction model based on a phage genome-lifestyle dataset is applied. Lifestyle prediction: The lifestyle of the phages is predicted with the uploaded phage genome. (4) Annotation comparison. Data manipulation: Genome annotation files (phaster.txt, RAST.xls, Pharokka.gff) are manipulated with built-in functions. Annotation comparison table: An annotation comparison table is generated using built-in functions.

To demonstrate the functions and the scope of application of PhageGE, we herein describe the results of a case study using PhageGE, including phage whole-genome data (i.e., .fasta), a phylogenetic tree file (i.e., .tre), and genome annotation data (i.e., .xls, .txt and .gff), which are collectively referred to as “Example Data” (Fig. 1). The complete set of Example Data used in the case studies can be accessed on the PhageGE GitHub repository [24].

### Phage phylogenetic analysis and visualization

**Figure 2:**
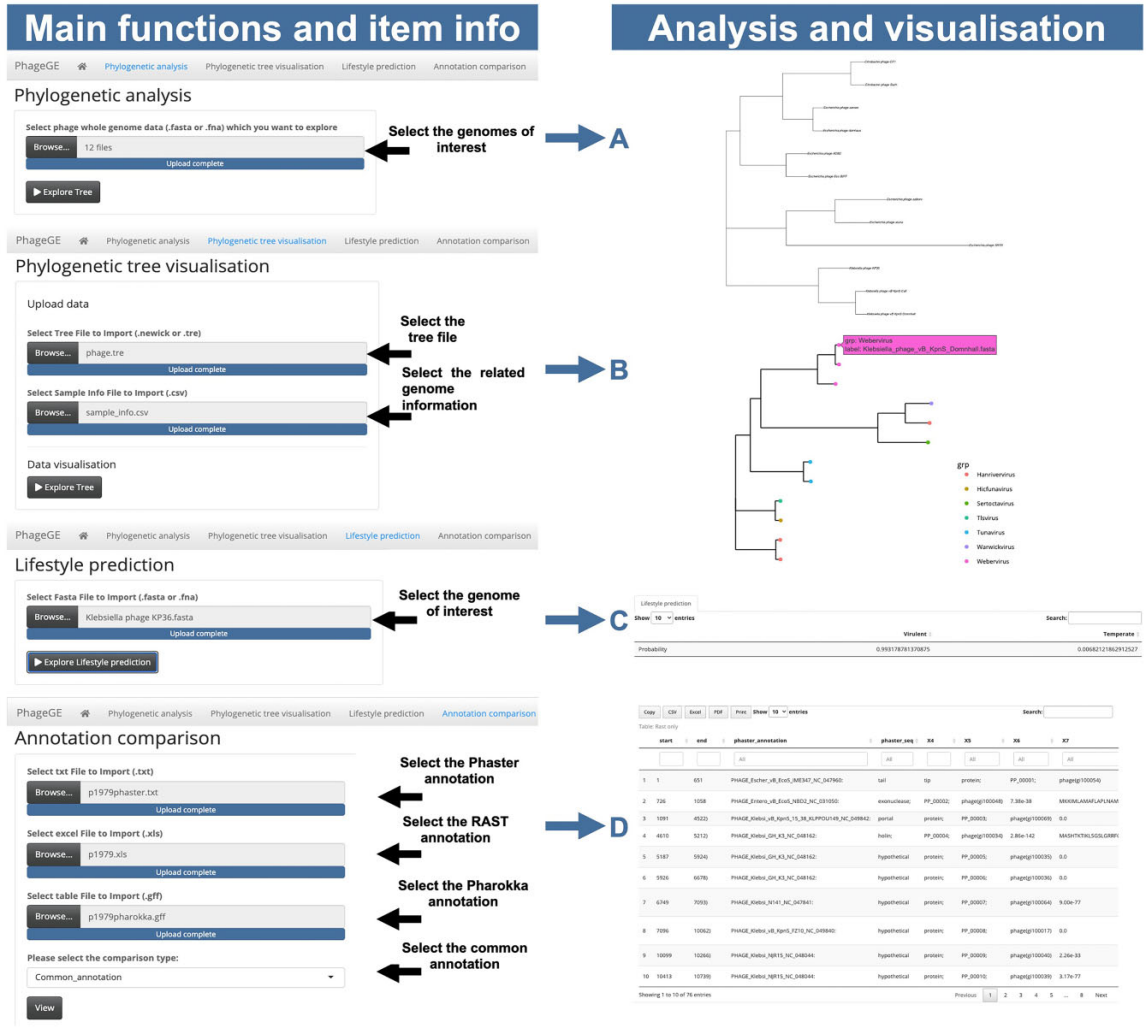
Overview of PhageGE and its related functions. The main functions and item information in PhageGE are illustrated in the figure, highlighting the steps for phylogenetic analysis, tree visualization, lifestyle prediction, and annotation comparison. (A) Phylogenetic analysis: Users can select the genomes of interest by uploading phage whole-genome data files (.fasta), selecting the layout of the tree (i.e., phylogram, cladogram, fan, radial, and tidy), and clicking the “Explore Tree” button to initiate the phylogenetic analysis. (B) Phylogenetic tree visualization: Users can upload a tree file (Newick or .tre format) and related genome information file (.csv). The tree visualization displays the phylogenetic relationships among the uploaded genomes, with detailed annotations. (C) Lifestyle prediction: Users can select a genome of interest for lifestyle prediction by uploading a fasta file (.fasta). By clicking the “Explore Lifestyle Prediction” button, the user can predict the lifestyle of the selected genome, displaying the results with relevant statistics. (D) Annotation comparison: Users can upload multiple annotation files (Phaster, RAST, and Pharokka) and select the type of comparison. The resulting comparison table displays the annotated features from each source, facilitating detailed comparative analysis.

**Figure 3:**
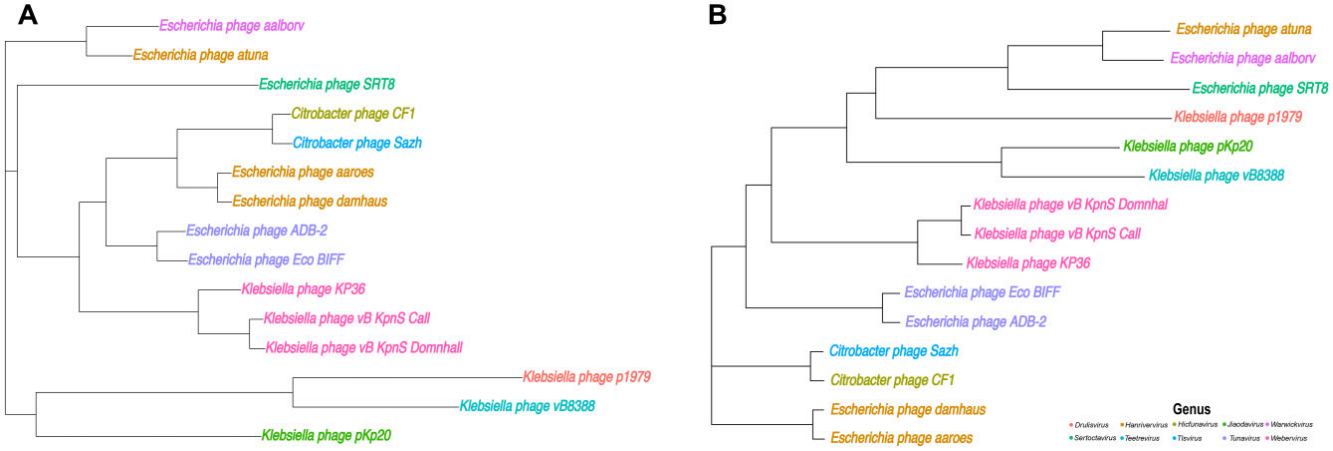
Comparison of phylogeny estimations from PhageGE and MSA. (A) Alignment-free phylogenetic trees of 15 phages inferred from WGS data and (B) the topology of the reference tree inferred from multiple sequence alignment of WGS. The trees illustrate the classification and related taxa positions, demonstrating the consistency and accuracy of PhageGE’s alignment-free approach in relation to the traditional MSA-based method.

To illustrate the phylogenetic analysis function in PhageGE and its application in clinical translation, we analyzed our GitHub example dataset, which consists of 15 phage genomes. The hosts of the 15 phage genomes in the phylogenetic analysis are from 3 different bacterial species: *Citrobacter freundii, Escherichia coli, and Klebsiella pneumoniae* (Fig. 2A). This dataset includes 1 anti-*Klebsiella* phage, pKp20, which was isolated in our lab and used in a clinical case [25]. In that case, a recurrent urinary tract infection (rUTI) was successfully treated with 4 weeks of adjunctive intravenous bacteriophage therapy, with no recurrence during a year of follow-up [25]. Both taxonomy information from phylogeny analysis and the lifestyle prediction played key roles in the selection of pKp20 over a wide range of phages [25]. The phage WGS data in the fasta format can be obtained either from NCBI or prepared locally using standard genome assembly pipelines (e.g., SPAdes) based on the previous BLASTn result [25]. To compare the results obtained from PhageGE with the multiple sequence alignment-based approach, we also conducted a multiple sequence alignment-based phylogenetic analysis using MAFFT v7.47 and fasttree v2.1.10, alongside the phylogenetic analysis using PhageGE. We first uploaded the selected fasta files or a multi-fasta file, which contains all phage genomes on the Phylogenetic Analysis page in PhageGE, then selected the layout of the tree (i.e., phylogram, cladogram, fan, radial, or tidy) and clicked the “Explore Tree” icon. The resulting phylogenetic tree, representing the relationships among the uploaded genomes, was generated using the built-in *k*-mer–based alignment-free phylogenetic approach, as detailed in the Methods section (Figs. 2A and 3A). To enhance the clarity, we manually highlighted the 15 phages with distinct colors according to their genus. Comparison of the phylogenetic trees generated by PhageGE and MAFFT revealed that both trees shared largely the same classification (e.g., positions of each phage and the related taxa) (Fig. 3). Moreover, PhageGE demonstrates a significant improvement in runtime efficiency. For example, on a 2-GHz CPU with a 64-GB RAM server, the runtimes of generating phylogenetics trees by PhageGE were 0.22 minutes for 15 phage genomes and 4.42 minutes for 146 phage genomes. In contrast, the multiple sequence alignment (MSA)–based approach (using tools like MAFFT along with FastTree) took 30 minutes and 296 minutes, respectively. This demonstrates that the performance of the phylogenetic analysis of PhageGE is accurate, fast, and comparable to the MSA-based approach.

**Figure 4:**
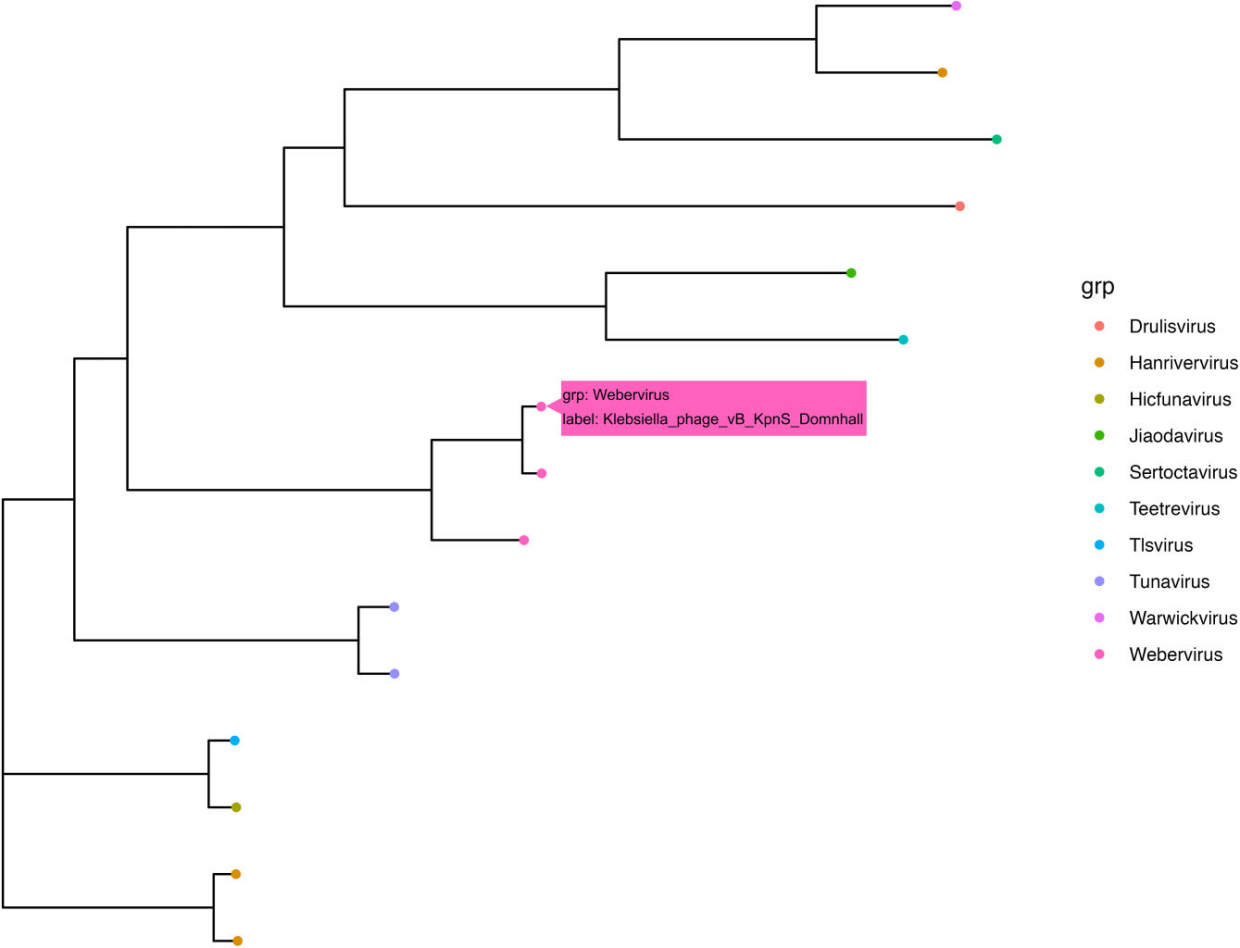
Interactive visualization of the phylogenetic tree of 15 phages. Each colored dot represents 1 phage, with the color indicating the associated taxa. The pink box illustrates the additional information that can be obtained by hovering the cursor over each dot.

The phylogenetic visualization function handles the phylogenetic tree along with diverse accompanying data. Its aim is to provide an interactive visualization platform that enhances the accessibility of phylogenetic data and facilitates the phylogenetic analysis of phage comparative genomics studies. The phylogenetic tree and associated data can be extracted using a built-in function within PhageGE. This function is illustrated using a tree file “phage.tre” obtained from phage phylogenetic analysis (whether generated by PhageGE or other phylogenetic analysis pipeline) and a sample information file named “sample_info.csv” containing the taxonomy information for all 14 phages (Fig. 2B). As shown in Fig. 4, each dot in the dendrogram represents 1 phage with the color indicating its taxonomic classification in the same genus. In addition, detailed information of each phage (e.g., name and taxonomy) can be easily accessed by hovering the cursor over the dot of interest (as indicated by the pink box in Fig. 4). This interactive feature allows users to dynamically integrate and visualize the underlying information in a user-friendly manner.

### Performance of phage lifestyle prediction

**Figure 5:**
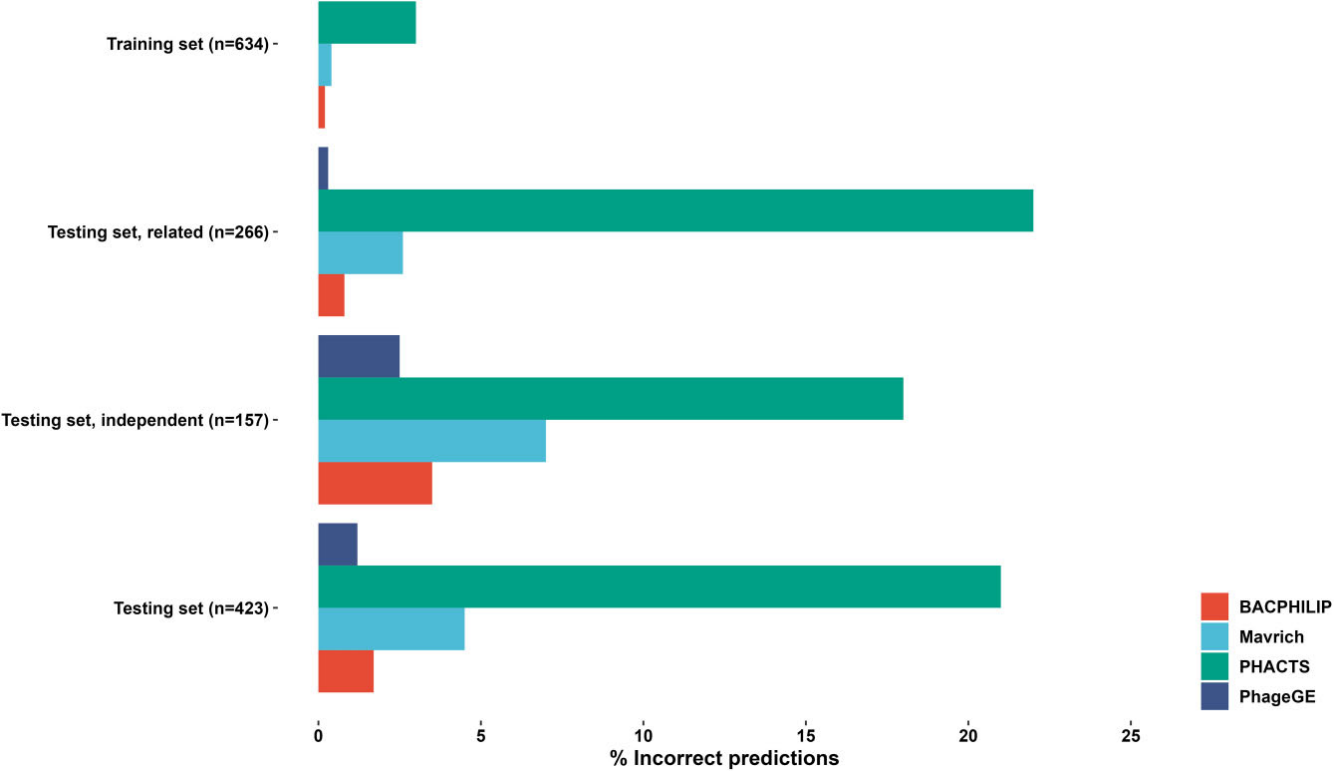
Comparison of classification accuracy of PhageGE with previously published tools across all datasets analyzed. Incorrect classification involves misidentifying the phage lifestyle (temperate or lytic).

The lifestyle prediction function builds on a random forest classifier that incorporates up-to-date conserved protein domains with the ability to classify temperate and lytic phages using WGS data. To evaluate its performance, we compared the function with other published tools using the dataset of 1,057 phages in the literature [26]. The PhageGE lifestyle prediction function achieved the lowest error rates (0%, 1.2%, 0.3%, and 2.5%, equivalent to 100%, 98.8%, 99.7%, and 97.5% classification accuracy, respectively) across all tested datasets, substantially outperforming those existing tools for phage lifestyle classification (Fig. 5). The prediction accuracy of PhageGE exceeded that of the most accurate existing tool, BACPHLIP, which had prediction accuracies of 99.8%, 98.3%, 99.2%, and 96.5%, respectively (Fig. 5). Similarly, WGS data for individual phages (e.g., *Klebsiella* phage KP36.fasta, vB8388.fasta, and FK1979.fasta from the example dataset described here) can be uploaded as input to generate the phage lifestyle probability table (Fig. 2C and Table 2). The result presented in Table 2 predicts that *Klebsiella* phages KP36 (a model phage in our laboratory), FK1979 and vB8388 [27] (2 phages isolated from hospital sewage, The First Affiliated Hospital of Wenzhou Medical University, China), and pKp20 (used in the rUTI clinical case study) [25] are highly likely lytic phages, with the probability of 99.3%, 95.6%, and 96.9%, respectively. Meanwhile, the 4 phages from the NCBI in Table 2 NC_017985, NC_027339, NC_009815, and NC_019768 are highly likely temperate phages. This function empowers users to rapidly analyze the lifestyle of a phage of interest *in silico* with high prediction accuracy, providing key insights into the intricate phage ecosystems and enabling optimal design of phage therapy.

**Table 2:** Lifestyle prediction for 8 different phages

	Lytic	Temperate
**KP36**	0.993	0.007
**FK1979**	0.956	0.044
**vB8838**	0.969	0.031
**pKp20**	0.974	0.026
**NC_017985**	0	1
**NC_027339**	0.002	0.998
**NC_009815**	0.016	0.984
**NC_019768**	0.01	0.99

### Comparison of phage genome annotation

Notably, PhageGE also provides a function to compare phage genome annotations obtained from different pipelines (i.e., Pharokka, Phaster, and RAST). This analysis involves the integration of R package flextable, which allows for the generation of downloadable comparison results in multiple formats (e.g., csv, Excel, and PDF). The user interface offers the flexibility to rank the results based on multiple parameters (e.g., location and/or length of the coding sequence [CDS]). In the case study presented here, we used PhageGE to compare genome annotations of *Klebsiella* phages KP36, vB8838, and FK1979 generated from Phaster, RAST, and Pharokka (Fig. 2D). By selecting “common_annotation,” a table with 75, 45, and 51 genes that were annotated in all 3 pipelines was generated for KP36, vB8838, and FK1979, respectively. We also identified 17, 7, and 12 unique genes from the Pharokka pipeline by selecting the “Pharokka_only” option. To gain a better understanding of those unique annotated genes, PhageGE allows users to directly copy and download both the nucleotide and amino acid sequences associated with the genes from the interactive table. This feature facilitates further investigation of these unique annotations.

## Discussion

With the dramatic rise in MDR bacterial infections, phage therapy has emerged as a safe and potentially effective alternative treatment option to antibiotics [28]. However, the development of effective phage therapies is complex, involving the isolation, culturing, characterization, and timely preparation of efficacious phages. Traditionally, this process is time-consuming and costly [29, 30]. Nevertheless, with the next-generation sequencing techniques, it has become possible to rapidly and cost-effectively characterize phages. Despite this advancement, there is a paucity of intuitive tools available for phage genomics, with the majority requiring operation in command-line mode. The availability of large phage genomic datasets presents unique opportunities to develop bioinformatics tools that aid in phage biology and pharmacology research. The use of computational methods to study phages has shown promise in generating novel insights, such as phylogeny and lifestyle, through bioinformatic analysis [18, 26, 31]. However, there is currently no single tool available that encompasses all those functions (e.g., phylogenetic analysis, tree visualization, lifestyle prediction, and genome annotation comparison) in the web server platform. Herein, we describe the development of the PhageGE web server GUI streamlined for user-friendly phage genomic analysis.

PhageGE is a novel, user-friendly GUI application for the interactive analysis of phage genomes. The overarching goal of PhageGE is to provide an interactive analysis and visualization platform for the rapid exploration of phage genomic associations, thereby promoting efficient genomic data-driven discovery of phage therapy. PhageGE comprises a set of functions for phage genomic analysis, including phylogenetic analysis, tree visualization, lifestyle prediction, and genome annotation comparison. While current tools like PhaGAA can provide lifestyle reorganization analysis, their primary utility lies in analyzing phage lifestyle for their preferred phage dataset (e.g., gut flora of human neonates) [32]. In contrast, PhageGE integrates a more comprehensive dataset with a wide range of phage genomes, allowing for broader and deeper exploration of phage lifestyles. Moreover, the comparison of annotations from different pipelines highlights the key role of PhageGE in advancing phage genomics through enhanced analysis and visualization functions. To exemplify the utility of PhageGE, we investigated the phylogeny, lifestyle, and annotation comparison of *Klebsiella* phages KP36, vB8838, and FK1979, which were independently isolated in 2 different countries. Our findings demonstrate that the various functions of PhageGE yield comparable or better results than existing state-of-the-art approaches. These results highlight the significant potential of PhageGE in analyzing various phage genomic features using phage WGS data.

Notably, PhageGE requires only phage WGS data as the input for conducting the related analysis. The phage phylogenetic analysis function takes phage WGS in the fasta format as input and applies an alignment-free phylogenetic approach to infer phylogenetic relationships. Compared to current phylogenetic analysis pipelines (i.e., multiple sequence alignment–based phylogenetic analysis), analysis from PhageGE showed similar phage phylogeny information in a shorter computing time (approximately 13 seconds versus 30 minutes for 15 phage genomes). Moreover, the result from phylogenetic analysis can be easily exported in various graphical formats (e.g., SVG, PDF, and JPEG) and textual formats (e.g., Newick and Nexus) and can be interactively managed and viewed through our designed user interface. In addition, PhageGE introduces an enhanced phage lifestyle prediction function, using a machine learning approach with updated databases for conserved protein domains. The overall approaches applied for both phylogenetic analysis and lifestyle prediction demonstrate that analyses results from PhageGE are comparable to previously published tools (Figs. 3 and 5), showing its effectiveness in accurately analyzing phage phylogeny and predicting phage lifestyle. Notably, PhageGE incorporates a function of annotation comparison to facilitate the efficient organization of genome annotation files derived from different annotation pipelines. This feature allows users to efficiently compare genome annotation data obtained with different tools. Overall, all 4 functions from PhageGE serve as a guide for the exploration of phage genomic features and will expedite the clinical translation of phage therapy.

## Conclusion

In conclusion, PhageGE is the first user-friendly tool for the analysis of phage genomes, offering improved functions compared to existing tools without the need for considerable programming skills. Uniquely incorporating features like phylogenetic analysis, interactive tree visualization, lifestyle prediction, and genome annotation comparison, we anticipate that PhageGE will become an instrumental bioinformatic web server for phage genomic analysis, guiding experimental validations and advancing the development of phage therapy.

## Methods

### Implementation

PhageGE 1.0 (RRID:SCR_025380) was developed in R and is hosted on Shinyapps. This application seamlessly integrates various R packages, including Rshiny, seqinr, Biostrings, ape, textmineR, tidyverse, ggtree, ploty, ggplot, reticulate, and pyhmmer [22, 33–39]. Furthermore, it incorporates several key functions, including *k*-mer–based phylogeny estimation, phylogenetic tree visualization, lifestyle prediction, and annotation comparison. To use PhageGE, input files in the standard WGS fasta format are required, along with textual tables in standard formats (e.g., csv or xlsx) containing sequence details and annotation information. The workflow is illustrated in Fig. 1.

### Phage genomic analysis pipeline

The functionalities offered in the web interface of PhageGE utilize WGS fasta files for phylogenetic analysis and lifestyle prediction. Users can input tree files (e.g., Newick or Nexus) and textual files (i.e., csv or xlsx) for phylogenetic tree visualization and genome annotation comparisons. Using these standard formats as input files facilitates effective use and simplifies data export for users.

### Phylogenetic analysis and phylogenetic tree visualization

The phylogenetic analysis function enables fast and efficient analysis of phage phylogeny. It includes phylogeny reconstruction based on the input WGS data and visualization of phylogenetic information. This function incorporates a *k*-mer–based alignment-free phylogenetic approach [40]. Alignment-free phylogenetic approaches offer a scalable alternative for inferring phylogenetic relationships and computing local alignment boundaries from WGS data [41, 42]. This approach is particularly robust for genome sequences that exhibit genetic recombinations and rearrangements. It has demonstrated the ability to accurately reconstruct biologically relevant phylogenies with thousands of microbial genomes [43–45]. The description of this function is briefly outlined below.

Consider a sequence consisting of 4 characters (A, T, C, G) of length *k* (“*k*-mer”), described by Equation 1. There are 4*^k^* possible *k*-mers (Equation 2), which can serve as features of each genome. The value assigned to a specific *k*-mer feature will correspond to the number of occurrences of that *k*-mer in the genome. Using these *k*-mer features, a data matrix is generated with dimensions of the numbers of genomes of interest (*n* columns) by 4*^k^* rows. To establish a representative probability distribution of the 4*^k^ k*-mers, each row of the data matrix is normalized by its row total. This normalization results in a feature-frequency profile (*F_k_*, described by Equation 3) for each *k*-mer sequence [40]. The Jensen–Shannon divergence (*D_k_*, described by Equation 4) is then employed to estimate the genome pairwise distances [46]. Subsequently, the resulting distance matrix is used as an input for a clustering algorithm (e.g., neighbor-joining algorithm) to summarize the relatedness of the phage genomes and construct a phylogenetic tree [36].


(1) \begin{eqnarray*}
{C}_k = \ \left\langle {{C}_{k,1},\ {C}_{k,2} \cdots {C}_{k,m}} \right\rangle
\end{eqnarray*}

(2) \begin{eqnarray*}
m = {4}^k
\end{eqnarray*}

(3) \begin{eqnarray*}
{F}_{{n}_i,\ k} = \frac{{{C}_{{n}_i,\ {k}_m}\ }}{{\mathop \sum \nolimits_{{n}_i} {C}_{{n}_i,\ k}}}
\end{eqnarray*}

(4) \begin{eqnarray*}
{D}_k = JS\left( {{F}_{{n}_1,\ k},\ {F}_{{n}_i,\ k}} \right) \end{eqnarray*}

An interactive visualization of a phylogenetic tree was generated from the phylogenetic analysis function or a customized phylogenetic tree that includes additional information, such as species classification, duplication events, and bootstrap values. It is implemented using ggtree and ploty R packages [22], ensuring the ability to handle most common tree formats (e.g., Newick, Nexus, and tre).

### Lifestyle prediction

The Lifestyle Prediction function in PhageGE generates a phage lifestyle probability table based on the input of phage WGS data. This function adapted previously reported approaches into our user-friendly interface [18, 23, 26]. By employing an improved search function (i.e., searching a sequence file against the build-in hidden Markov model database), PhageGE provides an efficient way to predict phage lifestyle based on the phage genomic information.

In brief, we first conducted a search in the Conserved Domain Database (accessed: 11/2023) to collect protein domains from temperate phages [47]. The following key words were used to identify relevant protein domains: “temperate,” “lysogen,” “integrase,” “excisionase,” “recombinase,” “transposase,” “parA|parB,” and “xerC|xerD.” We obtained a total of 477 protein domains from the initial collection, which were then subjected to a careful manual curation and filtration (e.g., minimal domain length >30 and validated in the existing experimental data), resulting in a refined set of 261 protein domains. Next, a lifestyle classification model was trained and tested using a published dataset consisting of 1,057 phages from 6 different families (Inoviridae, Myoviridae, Plasmaviridae, Podoviridae, Siphoviridae, and Tectiviridae) across 55 host genera, with known genome and lifestyle information [26]. The dataset was randomly split into training and testing sets, with a ratio of 60:40 (634 phages in the training set and 423 phages in the testing set). At this stage, the testing set was fully set aside for subsequent descriptions related to model training and development. For each genome sequence in the training set, we generated a list of all possible 6-frame translation sequences that were at least 40 amino acids long. HMMER3 was then used to search for the presence or absence of the various protein domains listed above, resulting in a vector for each phage describing the presence (1) or absence (0) of each domain [48]. This information allowed us to filter the initial set of 477 putatively useful protein domains down to the final set of 261. Subsequently, a random forest classifier was fitted to the training set of phage genomes, and cross-validation was employed to fine-tune the model hyperparameters. The “best”-performing model was then selected by choosing the hyperparameters that yielded the highest minimum accuracy across the independent validation set tests. The parameters of that model were then refitted to the entire training set data, resulting in the final model.

### Annotation comparison

The Rapid Annotation using Subsystem Technology (RAST) server (RRID:SCR_014606) was developed in 2008 to annotate microbial genomes based on the manually curated SEED database (RRID:SCR_002129) [49]. The PHAge Search Tool—Enhanced Release (PHASTER) was specifically designed to identify and annotate prophage sequences within bacteria using prophage/virus databases [50]. More recently, another phage annotation tool, Pharokka, has been developed using PHROGS, CARD, and VFDB databases [51]. Since these pipelines employ different databases for phage genome annotation, it is possible to obtain different annotations from each pipeline. To provide more comprehensive annotation results, there is an urgent need for annotation comparison tables that incorporate all annotation information from RAST, PHASTER, and Pharokka. The Annotation Comparison function in PhageGE generates interactive tables that display comments and differing genome annotation information obtained from RAST, PHASTER, and Pharokka. This comparison includes checking the coding regions and related annotations from each pipeline. Moreover, it provides an overview of common and different annotation counts, facilitating the tracking of differences between the 3 pipelines. This function is implemented using the flextable, tidyselect, data.table, and tidyverse packages [38].

## Code Availability and Requirements

- Project name: PhageGE (Phage Genome Exploration)
- Project homepage: https://github.com/JinxinMonash/PhageGE [24]
- Operating system(s): Linux, Windows and MacOS (Table 1)
- Programming language: R
- License: MIT license
- RRID:SCR_025380

## Abbreviations

AMR: antimicrobial resistance; MDR: multidrug resistant; MSA: multiple sequence alignment; NCBI: National Center for Biotechnology Information; RAST: Rapid Annotation using Subsystem Technology; rUTI: recurrent urinary tract infection; WGS: whole-genome sequencing.

## Supplementary Material

giae074_GIGA-D-24-00040_Original_Submission

giae074_GIGA-D-24-00040_Revision_1

giae074_GIGA-D-24-00040_Revision_2

giae074_GIGA-D-24-00040_Revision_3

giae074_Response_to_Reviewer_Comments_Original_Submission

giae074_Response_to_Reviewer_Comments_Revision_1

giae074_Response_to_Reviewer_Comments_Revision_2

giae074_Reviewer_1_Report_Original_Submission Andre Mu -- 2/18/2024 Reviewed

giae074_Reviewer_1_Report_Revision_1 Andre Mu -- 7/15/2024 Reviewed

giae074_Reviewer_2_Report_Original_Submission Huaiqiu Zhu -- 4/7/2024 Reviewed

## Data Availability

In general, all data used in this work were from openly accessible public repositories and released with other publications under open-source licenses. The data used were solely for research purposes, and we confirm that they were not used for any other noncommercial or commercial purpose. The datasets supporting the results of this article are available in the GitHub repository, [24]. The data used as examples can be found in the release branch called “Example data” or “Example data.zip” within our repository. The GitHub repository also contains up-to-date tutorials. Snapshots of our code and other data further supporting this work are openly available in the *GigaScience* repository, GigaDB [52].
