# Supplementary material for: PhageGE: an interactive web platform for exploratory analysis and visualization of bacteriophage genomes
Source: Gigascience. 2024 Sep 25;13:giae074. doi: 10.1093/gigascience/giae074 (PMC11423353; doi:10.1093/gigascience/giae074)
Supplement: giae074_GIGA-D-24-00040_Original_Submission [file giae074_giga-d-24-00040_original_submission.pdf]

GigaScience

PhageGE: An interactive web platform for exploratory analysis and visualisation of bacteriophage genomes

--Manuscript Draft--

**Manuscript Number:**	GIGA-D-24-00040
**Full Title:**	PhageGE: An interactive web platform for exploratory analysis and visualisation of bacteriophage genomes
**Article Type:**	Technical Note
**Funding Information:**	Division of Microbiology and Infectious Diseases, National Institute of Allergy and Infectious Diseases (R01 AI156766) — Prof Jian Li
**Abstract:**	Background<br><br>Antimicrobial resistance is a serious threat to global health. Attributable to the stagnant antibiotic discovery pipeline, bacteriophages (phages) have been proposed as an alternative therapy for the treatment of infections caused by multidrug-resistant (MDR) pathogens. Phage genomic features play an important role in its pharmacology; however, our knowledge of phage genomics is sparse and the use of existing bioinformatic pipelines and tools requires considerable bioinformatic expertise. These challenges have substantially limited the clinical translation of phage therapy.<br><br>Findings<br><br>A user-friendly graphical interface application PhageGE (Phage Genome Explorer) was developed for the interactive analysis of phage genomes. The new R Shiny webserver, PhageGE, was designed for analysing phage whole-genome sequence (WGS) data. PhageGE also integrates several existing R packages and combines them with several newly developed functions to perform phylogeny analysis and lifestyle prediction. The webserver offers several additional key functions, including interactive phylogenetic tree visualisation and annotation comparison. The output from PhageGE can be exported directly with publication-quality images.<br><br>Conclusions<br><br>We anticipate that PhageGE will be a valuable tool for analysing phage genome data, thereby expediting the development of phage therapy. PhageGE is publicly available at http://phagege.com/.
**Corresponding Author:**	Jian Li<br>Monash Biomedicine Discovery Institute<br>AUSTRALIA
**Corresponding Author Secondary Information:**	
**Corresponding Author's Institution:**	Monash Biomedicine Discovery Institute
**Corresponding Author's Secondary Institution:**	
**First Author:**	Jinxin Zhao, Ph.D.
**First Author Secondary Information:**	
**Order of Authors:**	Jinxin Zhao, Ph.D.
	Jiru Han, Ph.D.
	Yu-Wei Lin, Ph.D.
	Yan Zhu, Ph.D.
	Michael Aichem
	Dimitar Garkov

Powered by Editorial Manager® and ProduXion Manager® from Aries Systems Corporation

	Phillip J. Bergen, Ph.D.
	Sue C. Nang, Ph.D.
	Jian-Zhong Ye, Ph.D.
	Tieli Zhou, Ph.D.
	Tony Velkov, Ph.D.
	Jiangning Song, Ph.D.
	Falk Schreiber, Ph.D.
	Jian Li, Ph.D.
**Order of Authors Secondary Information:**	

**Additional Information:**	
**Question**	**Response**
Are you submitting this manuscript to a special series or article collection?	No
**Experimental design and statistics**<br><br>Full details of the experimental design and statistical methods used should be given in the Methods section, as detailed in our Minimum Standards Reporting Checklist. Information essential to interpreting the data presented should be made available in the figure legends.<br><br>Have you included all the information requested in your manuscript?	Yes
**Resources**<br><br>A description of all resources used, including antibodies, cell lines, animals and software tools, with enough information to allow them to be uniquely identified, should be included in the Methods section. Authors are strongly encouraged to cite Research Resource Identifiers (RRIDs) for antibodies, model organisms and tools, where possible.<br><br>Have you included the information requested as detailed in our Minimum Standards Reporting Checklist?	Yes
**Availability of data and materials**	Yes

Powered by Editorial Manager® and ProduXion Manager® from Aries Systems Corporation

All datasets and code on which the conclusions of the paper rely must be either included in your submission or deposited in publicly available repositories (where available and ethically appropriate), referencing such data using a unique identifier in the references and in the "Availability of Data and Materials" section of your manuscript.<br><br>Have you have met the above requirement as detailed in our Minimum Standards Reporting Checklist?	

Manuscript

Click here to access/download;Manuscript;PhageGE-Main document 20240205.docx

PhageGE: An interactive web platform for exploratory analysis and visualisation of bacteriophage genomes

Jinxin Zhao[1, 2*], Jiru Han[3], Yu-Wei Lin[1], Yan Zhu[1, 4], Michael Aichem[5], Dimitar Garkov[5], Phillip J. Bergen[1], Sue C. Nang[1], Jian-Zhong Ye[6, 7], Tieli Zhou[6, 7], Tony Velkov[8], Jiangning Song[2, 9], Falk Schreiber[5, 10], Jian Li[1, 2*]

[1] Infection Program and Department of Microbiology, Biomedicine Discovery Institute, Monash University, Clayton, VIC, Australia

[2] Monash Biomedicine Discovery Institute-Wenzhou Medical University Alliance in Clinical and Experimental Biomedicine, Monash University, Clayton, VIC, Australia

[3] Population Health and Immunity Division, The Walter and Eliza Hall Institute of Medical Research, Parkville, VIC, Australia

[4] Systems Biology Center, Tianjin Institute of Industrial Biotechnology, Chinese Academy of Sciences, Tianjin, China

[5] Department of Computer and Information Science, University of Konstanz, Konstanz, Germany

[6] Key Laboratory of Clinical Laboratory Diagnosis and Translational Research of Zhejiang Province, Department of Clinical Laboratory, The First Affiliated Hospital of Wenzhou Medical University, Zhejiang, China

[7] Wenzhou Medical University-Monash Biomedicine Discovery Institute Alliance in Clinical and Experimental Biomedicine, The First Affiliated Hospital of Wenzhou Medical University,Wenzhou, China

[8] Department of Pharmacology, Biomedicine Discovery Institute, Monash University, Melbourne, VIC, Australia

[9] Department of Biochemistry and Molecular Biology, Biomedicine Discovery Institute, Monash University, Melbourne, VIC, Australia

[10] Faculty of Information Technology, Monash University, Clayton, VIC, Australia

Email addresses: JZ, jinxin.zhao@monash.edu; JH, han.ji@wehi.edu.au; YL, yu-wei.lin@monash.edu; YZ, Yan.Zhu@monash.edu; MA,, michael.aichem@uni-konstanz.de; DG, dimitar.garkov@uni-konstanz.de; PB, phillip.bergen@monash.edu; SN, sue.nang@monash.edu; JY, jzye89@163.com; TZ, wyztli@163.com; TV, tony.velkov@monash.edu; JS, jiangning.song@monash.edu; FS, falk.schreiber@uni-konstanz.de; JL, jian.li@monash.edu.

Running title: PhageGE for bacteriophage genomic analysis

*Corresponding authors:

Dr Jinxin Zhao, Tel: +61 3 99056288, Email: jinxin.zhao@monash.edu;

Professor Jian Li, Tel: +61 3 99039172 Fax: +61 0399056450; Email: jian.li@monash.edu.

Abstract

Background: Antimicrobial resistance is a serious threat to global health. Attributable to the stagnant antibiotic discovery pipeline, bacteriophages (phages) have been proposed as an alternative therapy for the treatment of infections caused by multidrug-resistant (MDR) pathogens. Phage genomic features play an important role in its pharmacology; however, our knowledge of phage genomics is sparse and the use of existing bioinformatic pipelines and tools requires considerable bioinformatic expertise. These challenges have substantially limited the clinical translation of phage therapy.
Findings: A user-friendly graphical interface application PhageGE (Phage Genome Explorer) was developed for the interactive analysis of phage genomes. The new R Shiny webserver, PhageGE, was designed for analysing phage whole-genome sequence (WGS) data. PhageGE also integrates several existing R packages and combines them with several newly developed functions to perform phylogeny analysis and lifestyle prediction. The webserver offers several additional key functions, including interactive phylogenetic tree visualisation and annotation comparison. The output from PhageGE can be exported directly with publication-quality images.

Conclusions: We anticipate that PhageGE will be a valuable tool for analysing phage genome data, thereby expediating the development of phage therapy. PhageGE is publicly available at http://phagege.com/.

Keywords: phage genome, biological web application, genomic analysis, phylogeny, lifestyle

Introduction

The rapid emergence and spread of antimicrobial resistance (AMR) is one of the three greatest threats to human health globally [1]. It is estimated that by 2050, life-threatening infections caused by antimicrobial-resistant pathogens will kill more people than any other diseases [2]. Of particular concern is the increased prevalence of infections caused by Gram-negative pathogens, which are more difficult to treat than Gram-positive pathogens [3]. Given the sluggish global antibiotic pipeline [4], bacteriophages (phages) have attracted significant attention over the last decade as a potential alternative therapy for bacterial infections [5]. Phages are bacterial viruses and the advantages of phage therapy over antibiotics include a narrow spectrum of activity, the capacity to multiply at the infection site, and safety [6-8]. Optimising phage therapy in patients requires key pharmacological information, including infection cycle, gene content and phage taxonomy [9, 10]. For example, temperate phages do not immediately lyse bacterial host cells and have an inherent capacity to mediate the transfer of genes between bacteria, potentially facilitating increased bacterial virulence and AMR. In contrast, lytic phages kill bacteria upton infection and are commonly used for the treatment of MDR bacterial infections in patients [11-14].

Multi-omics have the potential to expedite the clinical translation of phage therapy for the treatment of MDR bacterial infections [15]. For example, whole genome-based phylogenetic analysis offers significant advantages in understanding phage evolutionary dynamics and designing potential phage cocktails [16, 17]. Furthermore, combining whole-genome sequencing (WGS) with *in silico* prediction enables rapid prediction of phage life style [18]. Several popular bioinformatic pipelines and tools are available for such analyses, including MAFFT, RAxML and IQ-TREE (for multiple sequence alignment and phylogenetic analysis) [19-21], ggtree (for the visualisation

of phylogeny data) [22], PHACTS and BACPHLIP (for phage lifestyle prediction) [18, 23]. However, utilising these tools requires proficient programming skills, therefore, a biologist-friendly pipeline for phage genomic analyses is urgently needed to address the aforementioned limitations in phage genomic analysis.

Here, we developed an integrated webserver platform PhageGE that offers four key functionalities, namely phage phylogenetic analysis, tree visualisation, lifestyle prediction, and manipulation of phage genome annotation datasets. PhageGE is different from the existing phage genomic analysis tools, in that it facilitates the seamless export of all associated results in publication-ready format without requiring complex procedures and long running time. Overall, PhageGE provides a biologist-friendly interface to streamline phage genomic analysis with WGS data.

Results

The PhageGE webserver was designed to ensure biologist-friendliness and compatibility with major web browsers, including Google Chrome, Mozilla Firefox, Apple Safari and Microsoft Edge (**Table 1**).

Webserver submission and case studies

To demonstrate the functions and application scope of PhageGE, we herein describe the results of presenting PhageGE with an example dataset, referred to the "Example Data" (**Figure 1**). The complete set of Example Data used in the case studies can be accessed on the PhageGE GitHub repository (https://github.com/JinxinMonash/PhageGE).

Phage phylogenetic analysis and visualisation

To illustrate the phylogenetic analysis function in PhageGE, we employed our GitHub example dataset which consists of 14 phage genomes (*Citrobacter*, *Escherichia*, and *Klebsiella*) from 9 different genera (**Figure 2A**). The WGS data in the .fna or .fasta format can be either obtained from NCBI or prepared locally using standard genome assembly pipelines (e.g., SPAdes). To compare the results obtained from PhageGE with the multiple sequence alignment-based approach, we also conducted a multiple sequence alignment-based phylogenetic analysis using MAFFT v7.47 alongside the phylogenetic analysis conducted in PhageGE. After uploading the selected genomes on the Phylogenetic Analysis page in PhageGE and clicking the “Explore Tree” icon, the resulting phylogenetic tree representing the relationships associated with the uploaded genomes is generated (**Figures 2A and 3A**). To enhance the clarity, we manually highlighted the 14 phages with distinct colours according to their genus. Comparison of the phylogenetic trees generated by PhageGE and MAFFT revealed that both trees shared largely the same classification (e.g., positions of each phage and the related taxa) (**Figure 3**). This demonstrates that the phylogenetic analysis performance of PhageGE is accurate and comparable to the multiple sequence alignment-based approach.

The phylogenetic visualisation function handles the phylogenetic tree along with diverse accompanying data. Its aim is to provide an interactive visualisation platform that improves the reusability of phylogenetic data and facilitates the phylogenetic analysis of phage comparative genomics studies. The phylogenetic tree and associated data can be extracted using a built-in function within PhageGE. This function is illustrated using a tree file “phage.tre” obtained from phage phylogenetic analysis (whether generated by PhageGE or other phylogenetic analysis pipeline) and a sample information file named “sample_info.csv” containing the taxonomy

information for all 14 phages (**Figure 2B**). As shown in **Figure 4**, each dot in the dendrogram represents one phage with the colour indicating its taxonomic classification in the same genus. In addition, detailed information of each phage (e.g., name and taxonomy) can be easily accessed by hovering the cursor over the dot of interest (as indicated by the pink box in **Figure 4**). This interactive feature allows users to dynamically integrate and visualise the underlying information in a user-friendly manner.

Performance of phage lifestyle prediction

The lifestyle prediction function builds on a Random Forest classifier that incorporates up-to-date conserved protein domains with the ability to classify temperate and lytic phages using WGS data. To evaluate its performance, we compared the function with other published tools using the dataset of 1,057 phages in the literature [24]. The PhageGE lifestyle prediction function achieved the lowest error rates (0%, 1.2%, 0.3% and 2.5%, equivalent to 100%, 98.8%, 99.7% and 97.5% classification accuracy, respectively) across all tested datasets, substantially outperforming those existing tools for phage lifestyle classification (**Figure 5**). The prediction accuracy of PhageGE exceeded that of the most accurate existing tool, BACPHLIP, which had prediction accuracies of 99.8%, 98.3%, 99.2% and 96.5%, respectively (**Figure 5**). Similarly, WGS data for individual phages (e.g., *Klebsiella* phage KP36.fasta, vB8388.fasta and FK1979.fasta from the example dataset described here) can be uploaded as input to generate the phage lifestyle probability table (**Figure 2C** and **Table 2**). The result presented in **Table 2** indicates that *Klebsiella* phages KP36 (a model phage in our laboratory), FK1979 and vB8388 [25] (two phages isolated from hospital sewage, The First Affiliated Hospital of Wenzhou Medical University, China) are highly likely to be lytic phages, with the probability of 99.3%, 95.6% and 96.9%, respectively. This

function empowers users to rapidly analyse the lifestyle of a phage of interest *in silico* with high prediction accuracy, which provides key insights into the intricate phage ecosystems and enables optimal design of phage therapy.

Comparison of phage genome annotation

Notably, PhageGE also provides a function to compare phage genome annotations obtained from different pipelines (i.e., Pharokka, Phaster and RAST). This analysis involves the integration of R package flextable, which allows for the generation of downloadable comparison results in multiple formats (e.g., csv, Excel and PDF). The user interface offers the flexibility to rank the results based on multiple parameters (e.g., location of the coding sequence [CDS] and the length of the CDS). In the case study presented here, we used PhageGE to compare genome annotations of *Klebsiella* phages KP36, vB8838 and FK1979 generated from Phaster, RAST and Pharokka (**Figure 2D**). By selecting “common_annotation”, a table with 75, 45, 51 genes that were annotated in all three pipelines were generated for KP36, vB8838 and FK1979, respectively. We also identified 17, 7 and 12 unique genes, respectively, from the Pharokka pipeline by selecting “Pharokka_only” option. To gain a better understanding of those unique annotated genes, PhageGE allows users to directly copy and download both nucleotide sequences and amino acid sequences associated with the genes from the interactive table. This feature facilitates further investigation of these unique annotations.

Discussion

With the dramatic rise in MDR bacterial infections, phage therapy has emerged as a safe and potentially effective alternative treatment option to antibiotics [26]. Notwithstanding, the development of effective phage therapies is complex, involving the isolation, culturing, characterisation and timely preparation of efficacious phages. Traditionally, this process is time-consuming and costly [27, 28]. However, with the next-generation sequencing techniques, it has become possible to rapidly and cost-effectively characterise phages. Nevertheless, there is a paucity of intuitive tools available for phage genomics, with the majority requiring operation in command-line mode. The availability of large phage genomic datasets presents unique opportunities to develop bioinformatics tools that aid in phage biology and pharmacology research. The use of computational methods to study phages have shown promise in generating novel insights, such as phylogeny and lifestyle, through bioinformatic analysis [18, 24, 29]. However, there is currently no single tool available that encompasses all those functions (e.g., phylogenetic analysis, tree visualisation, lifestyle prediction and genome annotation comparison) in the webserver platform. Herein, we describe the development of the PhageGE webserver GUI streamlined for user-friendly phage genomic analysis.

PhageGE is a novel, biologist-friendly GUI application for the interactive analysis of phage genomes. The overarching goal of PhageGE is to provide an interactive analysis and visualisation platform for the rapid exploration of phage genomic associations, thereby promoting efficient genomic data-driven discovery of phage therapy. PhageGE comprises a set of functions for phage genomic analysis, including phylogenetic analysis, tree visualisation, lifestyle prediction and genome annotation comparison. To exemplify the utility of PhageGE, we investigated the phylogeny, lifestyle, and annotation comparison of *Klebsiella* phages KP36, vB8838 and FK1979

which were independently isolated in two different countries. Our findings demonstrate that the various functions of PhageGE yield comparable and even better results than the existing state-of-the-art approaches. These results highlight the significant potential of PhageGE in analysing various phage genomic features using phage WGS data.

Notably, PhageGE requires only phage WGS data as the input for conducting the related analysis. The phage phylogenetic analysis function takes phage WGS in the fasta format as the input and applies an alignment-free phylogenetic approach to infer phylogenetic relationships. Compared to current phylogenetic analysis pipelines (i.e., multiple sequence alignment-based phylogenetic analysis), analysis from PhageGE showed similar phage phylogeny information in a shorter computing time (~2 mins versus 1 hour 11 mins for 14 phage genomes). Moreover, the result from phylogenetic analysis can be easily exported in various graphical formats (e.g., SVG, PDF and JPEG) and textual formats (e.g., Newick and Nexus), and be interactively managed and viewed through our designed user interface. In addition, PhageGE introduces an enhanced phage lifestyle prediction function, using a machine-learning approach with updated databases for conserved protein domains. The overall approaches applied for both phylogenetic analysis and lifestyle prediction demonstrate that analyses results from PhageGE are comparable to previously published tools (**Figures 3** and **5**), showing its effectiveness in accurately analysing phage phylogeny and predicting phage lifestyle**.** Notably, PhageGE incorporates a function of annotation comparison to facilitate the efficient organisation of genome annotation files derived from different annotation pipelines. This feature allows users to efficiently compare genome annotation data obtained with different tools. Overall, all fours functions from PhageGE

serve as a guide for the exploration of phage genomic features and will expedite the clinical translation of phage therapy.

Conclusion

In conclusion, PhageGE is the first biologist-friendly tool for the analysis of phage genomes, offering improved functions compared to existing tools without the need for considerable programming skills. Uniquely incorporating features like phylogenetic analysis, interactive tree visualisation, lifestyle prediction, and genome annotation comparison, we anticipate that PhageGE will become an instrumental bioinformatic webserver for phage genomic analysis, guiding experimental validations and advancing the development of phage therapy.

Methods

Implementation

PhageGE 1.0 was developed in R and is hosted at the Shinyapps. This application seamlessly integrates various R packages, including Rshiny, seqinr, Biostrings, ape, textmineR, tidyverse, ggtree, ploty, ggplot, reticulate and pyhmmer [22, 30-36]. Furthermore, it incorporates several key functions, including *k*-mer-based phylogeny estimation, phylogenetic tree visualisation, lifestyle prediction and annotation comparison. To use PhageGE, input files in the standard WGS fasta format are required, along with textual tables in standard formats (e.g., csv or xlsx) containing sequence details and annotation information. The workflow is illustrated in **Figure 1**.

Phage genomic analysis pipeline

The functionalities offered in the web interface of PhageGE utilise WGS fasta files for phylogenetic analysis and lifestyle prediction. Users can input tree files (e.g., Newick or Nexus) and textual files (i.e., csv or xlsx) for phylogenetic tree visualisation and genome annotation comparisons. Using these standard formats as input files facilitates the effective use and simplifies data export for users.

Phylogenetic analysis and phylogenetic tree visualisation

The phylogenetic analysis function enables fast and efficient analysis of phage phylogeny. It includes phylogeny reconstruction based on the input WGS data and visualisation of phylogenetic information. This function incorporates a *k*-mer-based alignment-free phylogenetic approach [37]. Alignment-free phylogenetic approaches offer a scalable alternative for inferring phylogenetic relationships and computing local alignment boundaries from WGS data [38, 39]. This approach is particularly robust for genome sequences that exhibit genetic recombinations and rearrangements. It has demonstrated the ability to accurately reconstruct biologically relevant phylogenies with thousands of microbial genomes [40-42]. The description of this function is briefly outlined below.

Consider a sequence consisting of four characters (A, T, C, G) of length k (‘*k*-mer’), described by **Equation 1**. There are 4^k possible *k*-mers (**Equation 2**), which can serve as features of each genome. The value assigned to a specific *k*-mer feature will correspond to the number of occurrences of that *k*-mer in the genome. Using these *k*-mer features, a data matrix is generated with dimensions of the numbers of genomes of interest (n columns) by 4^k rows. To establish a representative probability distribution of the 4^k *k*-mers, each row of the data matrix is normslised by its row total. This normalisation results in feature-frequency profile (F_k, described by **Equation 3**) for

each *k*-mers sequence [37]. The Jensen-Shannon divergence (D_k, described by **Equation 4**) is then employed to estimate the genome pairwise distances [43]. Subsequently, the resulting distance matrix is used as an input for a clustering algorithm (e.g., neighbor-joining algorithm) to summarise the relatedness of the phage genomes and construct a phylogenetic tree [33].

Equation 1: $C_k = < C_{k,1}, C_{k,2} \cdots C_{k,m} >$

Equation 2: $m = 4^k$

Equation 3: $F_{n_i,k} = \frac{C_{n_i,k_m}}{\sum_{n_i} C_{n_i,k}}$

Equation 4: $D_k = JS(F_{n_1,k}, F_{n_i,k})$

An interactive visualisation of a phylogenetic tree was generated either from the phylogenetic analysis function or a customised phylogenetic tree that includes additional information, such as species classification, duplication event and bootstrap value. It is implemented using ggtree and ploty R packages [22], ensuring the ability of handling most common tree formats (e.g., Newick, Nexus and tre).

Lifestyle prediction

The Lifestyle Prediction function in PhageGE generates a phage lifestyle probability table based on the input of phage WGS data. This function adapted previously reported approaches into our user-friendly interface [18, 23, 24]. By employing an improved searching function (i.e. searching a sequence file against the build-in HMM [Hidden Markov Model] database), PhageGE provides an efficient way to predict phage lifestyle based on the phage genomic information.

In brief, we first conducted a search in the Conserved Domain Database (accessed: 11/2023) to collect protein domains from temperate phages [44]. The following key words were to identify relevant protein domains: ‘temperate’, ‘lysogen’, ‘integrase’, ‘excisionase’, ‘recombinase’, ‘transposase’, ‘parA|parB’ and ‘xerC|xerD’. We obtained a total of 477 protein domains from the initial collection, which were then subjected to a careful manual curation and filtration (e.g., minimal domain length >30 and validated in the existing experimental data), resulting in a refined set of 261 protein domains. Next, a lifestyle classification model was trained and tested using a published dataset consisting of 1,057 phages (6 different families, *Inoviridae*, *Myoviridae*, *Plasmaviridae*, *Podoviridae*, *Siphoviridae*, *Tectiviridae*, across 55 host genera) with known genome and lifestyle information [24]. The dataset was randomly split into training and testing sets, with a ratio of 60:40 (634 phages in the training set and 423 phages in the testing set). At this stage, the testing set was fully set aside for subsequent descriptions related to model training and development. For each genome sequence in the training set, we generated a list of all possible 6-frame translation sequences that were at least 40 amino acids long. HMMER3 was then used to search for the presence or absence of the various protein domains listed above, resulting in a vector for each phage describing the presence (1) or absence (0) of each domain [45]. This information allowed us to filter the initial set of 477 putatively useful protein domains down to the final set of 261. Subsequently, a Random Forest classifier was fitted to the training set of phage genomes, and cross-validation was employed to fine-tune the model hyper-parameters. The ‘best’ performing model was then selected by choosing the hyper-parameters that yielded the highest minimum accuracy across the independent validation set tests. The parameters of that model were then re-fitted to the entire training set data, resulting in the final model.

Annotation comparison

The Rapid Annotation using Subsystem Technology (RAST) server was developed in 2008 to annotate microbial genomes based on the manually curated SEED database [46]. The PHAge Search Tool – Enhanced Release (PHASTER) was specifically designed to identify and annotate prophage sequences within bacteria using prophage/virus databases [47]. More recently, another phage annotation tool, Pharokka, has been developed using PHROGS, CARD and VFDB databases [48]. Since these pipelines employed different databases for phage genome annotation, it is possible to obtain different annotations from each pipeline. To provide a more comprehensive annotation results, there is an urgent need for annotation comparison tables that incorporate all annotation information from RAST, PHASTER and Pharokka. The Annotation Comparison function in PhageGE generates interactive tables that display comments and differing genome annotation information obtained from RAST, PHASTER and Pharokka. This comparison includes checking the coding regions and related annotations from each pipeline. Moreover, it provides an overview of common and different annotation counts, facilitating the tracking of differences between the three pipelines. This function is implemented using the flextable, tidyselect, data.table and tidyverse packages [35].

Code availability and requirements

- Project name: PhageGE (Phage Genome Exploration)
- Project homepage: https://github.com/JinxinMonash/PhageGE
- Operating system(s): Linux, Windows and MacOS (**Table 1**)
- Programming language: R

- License: MIT license

Data availability

In general, all data used in this work were from open-accessible public repositories, released with other publications under open-source licenses. All data used in this work were only for research purposes, and we confirm that we did not use these for any other noncommercial purpose or commercial purpose. The datasets supporting the results of this article are available in the Github repository, [https://github.com/JinxinMonash/PhageGE]. The data we used as examples can be found in the release branch called "Example data" or "Example data.zip" within our repository.The GitHub repository also contains up-to-date tutorials.

Competing interests

The authors declare that they have no competing interests.

Funding

This work was supported by the National Institute of Allergy and Infectious Diseases of the National Institutes of Health [grant number R01 AI156766 to J.L.]. The content is solely the responsibility of the authors and does not necessarily represent the official views of the National Institute of Allergy and Infectious Diseases or the National Institutes of Health.

Author's contributions

J.Z. collected all the data and participated in developing the webserver and writing the manuscript. J.H., Y.W.L., Y.Z., M.A. and D.G. and J.N.S. contributed to the development of the web server. P.J.B., S.N., J.Z.Y., T.L.Z. and T.V. took part in the discussion of the data. J.Z., F.S. and J.L. conceived the study, coordinated the work and contributed to writing the manuscript. All authors are involved in the discussion and finalisation of the manuscript.

Acknowledgements

J.Z. is a recipient of the 2022 Faculty of Medicine, Nursing and Health Sciences Bridging Fellowship, Monash University. J.L. is an Australian National Health Medical Research Council (NHMRC) Principal Research Fellow and T.V. is an REDI MPTConnect Industry Fellow. Y.W.L. is currently an employee of Certara, Australia and Co-Director of the Malaya Translational and Clinical Pharmacometrics Group, University of Malaya, Malaysia.

Reference

1. Murray CJ, Ikuta KS, Sharara F, Swetschinski L, Aguilar GR, Gray A, et al. Global burden of bacterial antimicrobial resistance in 2019: a systematic analysis. The Lancet. 2022;399 10325:629-55.
2. Luepke KH, Suda KJ, Boucher H, Russo RL, Bonney MW, Hunt TD, et al. Past, present, and future of antibacterial economics: increasing bacterial resistance, limited antibiotic pipeline, and societal implications. Pharmacotherapy: The Journal of Human Pharmacology and Drug Therapy. 2017;37 1:71-84.
3. Bassetti M and Garau J. Current and future perspectives in the treatment of multidrug-resistant Gram-negative infections. Journal of Antimicrobial Chemotherapy. 2021;76 Supplement_4:iv23-iv37.
4. Böttcher L, Gersbach H and Wernli D. Restoring the antibiotic R&D market to combat the resistance crisis. Science and Public Policy. 2022;49 1:127-31.
5. Uyttebroek S, Chen B, Onsea J, Ruythooren F, Debaveye Y, Devolder D, et al. Safety and efficacy of phage therapy in difficult-to-treat infections: a systematic review. The Lancet Infectious Diseases. 2022.
6. Kortright KE, Chan BK, Koff JL and Turner PE. Phage therapy: a renewed approach to combat antibiotic-resistant bacteria. Cell Host & Microbe. 2019;25 2:219-32.
7. Mousavi SM, Babakhani S, Moradi L, Karami S, Shahbandeh M, Mirshekar M, et al. Bacteriophage as a novel therapeutic weapon for killing colistin-resistant multi-drug-resistant and extensively drug-resistant gram-negative bacteria. Current Microbiology. 2021;78 12:4023-36.
8. Lin Y, Chang RY, Rao G, Jermain B, Han M-L, Zhao J, et al. Pharmacokinetics/pharmacodynamics of antipseudomonal bacteriophage therapy in rats: a proof-of-concept study. Clinical Microbiology and Infection. 2020;26 9:1229-35.
9. Hyman P. Phages for phage therapy: isolation, characterization, and host range breadth. Pharmaceuticals. 2019;12 1:35.
10. Gordillo Altamirano FL and Barr JJ. Phage therapy in the postantibiotic era. Clinical Microbiology Reviews. 2019;32 2:e00066-18.
11. Roach DR, Leung CY, Henry M, Morello E, Singh D, Di Santo JP, et al. Synergy between the host immune system and bacteriophage is essential for successful

phage therapy against an acute respiratory pathogen. Cell Host & Microbe. 2017;22 1:38-47. e4.

12. Harrison E and Brockhurst MA. Ecological and evolutionary benefits of temperate phage: what does or doesn't kill you makes you stronger. BioEssays. 2017;39 12:1700112.
13. Gill JJ and Hyman P. Phage choice, isolation, and preparation for phage therapy. Current Pharmaceutical Biotechnology. 2010;11 1:2-14.
14. Abedon ST, García P, Mullany P and Aminov R. Phage therapy: past, present and future. Frontiers Media SA, 2017, p. 981.
15. Debnath M, Prasad GB and Bisen PS. Omics technology. Molecular Diagnostics: Promises and Possibilities. Springer; 2010. p. 11-31.
16. Parmar KM, Dafale NA, Tikariha H and Purohit HJ. Genomic characterization of key bacteriophages to formulate the potential biocontrol agent to combat enteric pathogenic bacteria. Archives of Microbiology. 2018;200 4:611-22.
17. Philipson CW, Voegtly LJ, Lueder MR, Long KA, Rice GK, Frey KG, et al. Characterizing phage genomes for therapeutic applications. Viruses. 2018;10 4:188.
18. McNair K, Bailey BA and Edwards RA. PHACTS, a computational approach to classifying the lifestyle of phages. Bioinformatics. 2012;28 5:614-8.
19. Katoh K and Standley DM. MAFFT multiple sequence alignment software version 7: improvements in performance and usability. Molecular Biology and Evolution. 2013;30 4:772-80.
20. Stamatakis A. RAxML version 8: a tool for phylogenetic analysis and post-analysis of large phylogenies. Bioinformatics. 2014;30 9:1312-3.
21. Minh BQ, Schmidt HA, Chernomor O, Schrempf D, Woodhams MD, Von Haeseler A, et al. IQ-TREE 2: new models and efficient methods for phylogenetic inference in the genomic era. Molecular Biology and Evolution. 2020;37 5:1530-4.
22. Xu S, Li L, Luo X, Chen M, Tang W, Zhan L, et al. Ggtree: A serialized data object for visualization of a phylogenetic tree and annotation data. iMeta. 2022:e56.
23. Hockenberry AJ and Wilke CO. BACPHLIP: predicting bacteriophage lifestyle from conserved protein domains. PeerJ. 2021;9:e11396.

24. Mavrich TN and Hatfull GF. Bacteriophage evolution differs by host, lifestyle and genome. Nature Microbiology. 2017;2 9:1-9.

25. Zhao Y, Feng L, Zhou B, Zhang X, Yao Z, Wang L, et al. A newly isolated bacteriophage vB8388 and its synergistic effect with aminoglycosides against multi-drug resistant Klebsiella oxytoca strain FK-8388. Microbial Pathogenesis. 2023;174:105906.

26. Khan A, Rao TS and Joshi HM. Phage therapy in the Covid-19 era: Advantages over antibiotics. Current Research in Microbial Sciences. 2022;3:100115.

27. Camarillo-Guerrero LF, Almeida A, Rangel-Pineros G, Finn RD and Lawley TD. Massive expansion of human gut bacteriophage diversity. Cell. 2021;184 4:1098-109. e9.

28. Carrigy NB, Larsen SE, Reese V, Pecor T, Harrison M, Kuehl PJ, et al. Prophylaxis of Mycobacterium tuberculosis H37Rv infection in a preclinical mouse model via inhalation of nebulized bacteriophage D29. Antimicrobial Agents and Chemotherapy. 2019;63 12:e00871-19.

29. Martinez-Vaz BM and Mickelson MM. *In silico* phage hunting: bioinformatics exercises to identify and explore bacteriophage genomes. Frontiers in Microbiology. 2020;11:577634.

30. Sievert C. Interactive web-based data visualization with R, plotly, and shiny. Journal of the Royal Statistical Society Series A: Statistics in Society. 2020;184:1150.

31. Charif D and Lobry JR. SeqinR 1.0-2: a contributed package to the R project for statistical computing devoted to biological sequences retrieval and analysis. Structural approaches to sequence evolution. Springer; 2007. p. 207-32.

32. Pagès H, Aboyoun P, Gentleman R, DebRoy S. Biostrings: Efficient manipulation of biological strings. 2024. Biostrings (Version 2.70.2) https://bioconductor.org/packages/Biostrings.

33. Paradis E and Schliep K. ape 5.0: an environment for modern phylogenetics and evolutionary analyses in R. Bioinformatics. 2019;35 3:526-8.

34. Jones T, Doane W and Jones MT. Package ‘textmineR’. Functions for text mining and topic modeling. 2021. textmineR (Version 3.0.5) https://github.com/TommyJones/textmineR.

35. Wickham H and Wickham MH. Welcome to the tidyverse. Journal of Open Source Software. 2019. 4(43), 1686.

36. Wickham H and Wickham MH. ggplot2: Elegant Graphics for Data Analysis. Springer-Verlag New York . 2016. https://ggplot2.tidyverse.org/
37. Sims GE, Jun S-R, Wu GA and Kim S-H. Alignment-free genome comparison with feature frequency profiles (FFP) and optimal resolutions. Proceedings of the National Academy of Sciences. 2009;106 8:2677-82.
38. Mapleson D, Garcia Accinelli G, Kettleborough G, Wright J and Clavijo BJ. KAT: a K-mer analysis toolkit to quality control NGS datasets and genome assemblies. Bioinformatics. 2017;33 4:574-6.
39. Jain C, Koren S, Dilthey A, Phillippy AM and Aluru S. A fast adaptive algorithm for computing whole-genome homology maps. Bioinformatics. 2018;34 17:i748-i56.
40. Bernard G, Chan CX and Ragan MA. Alignment-free microbial phylogenomics under scenarios of sequence divergence, genome rearrangement and lateral genetic transfer. Scientific Reports. 2016;6 1:1-12.
41. Jacobus AP, Stephens TG, Youssef P, González-Pech R, Ciccotosto-Camp MM, Dougan KE, et al. Comparative genomics supports that Brazilian bioethanol Saccharomyces cerevisiae comprise a unified group of domesticated strains related to cachaça spirit yeasts. Frontiers in Microbiology. 2021;12:644089.
42. Bernard G, Greenfield P, Ragan MA and Chan CX. k-mer similarity, networks of microbial genomes, and taxonomic rank. Msystems. 2018;3 6:e00257-18.
43. Sims GE and Kim S-H. Whole-genome phylogeny of Escherichia coli/Shigella group by feature frequency profiles (FFPs). Proceedings of the National Academy of Sciences. 2011;108 20:8329-34.
44. Lu S, Wang J, Chitsaz F, Derbyshire MK, Geer RC, Gonzales NR, et al. CDD/SPARCLE: the conserved domain database in 2020. Nucleic Acids Research. 2020;48 D1:D265-D8.
45. Eddy SR. Profile hidden Markov models. Bioinformatics. 1998;14 9:755-63.
46. Overbeek R, Olson R, Pusch GD, Olsen GJ, Davis JJ, Disz T, et al. The SEED and the Rapid Annotation of microbial genomes using Subsystems Technology (RAST). Nucleic Acids Research. 2014;42 D1:D206-D14.
47. Arndt D, Grant JR, Marcu A, Sajed T, Pon A, Liang Y, et al. PHASTER: a better, faster version of the PHAST phage search tool. Nucleic Acids Research. 2016;44 W1:W16-W21.

48. Bouras G, Nepal R, Houtak G, Psaltis AJ, Wormald P-J and Vreugde S. Pharokka: a fast scalable bacteriophage annotation tool. Bioinformatics. 2023;39 1:btac776.

Table 1. Browsers and operating systems (OS) tested with PhageGE

OS	Chrome	Edge	Firefox	Safari
**Linux**	120.0	120.0	121.0	n/a
**MacOs**	107.0	108.0	107.0.1	15.6.1
**Windows**	105.0	108.0	107.0.1	n/a

n/a, not applicable

Table 2. Phage lifestyle prediction for *Klebsiella* phages KP36, FK1979 and vB8838

	Lytic	Temperate
**KP36**	0.993	0.007
**FK1979**	0.956	0.044
**vB8838**	0.969	0.031

Figures legends:

Figure 1. The workflow and application of PhageGE.

Figure 2. Overview of PhageGE and its related functions.

Figure 3. Comparison of phylogeny estimations from PhageGE and MSA. Alignment-free phylogenetic trees of 14 phages inferred from WGS data (**A**) and the topology of reference tree inferred from multiple sequence alignment of WGS (**B**).

Figure 4. Interactive visualisation of the phylogenetic tree of 14 phages. Each coloured dot represents one phage, with the colour indicating the associated taxa. The pink box illustrates the additional information that can be obtained by hovering the cursor over each dot.

Figure 5. Comparison of classification accuracy of PhageGE with previously published tools across all datasets analysed. Incorrect classification involves incorrectly identifying the phage lifestyle (temperate or lytic).

Figure 1 Click here to access/download;Figure;Figure 1.jpg

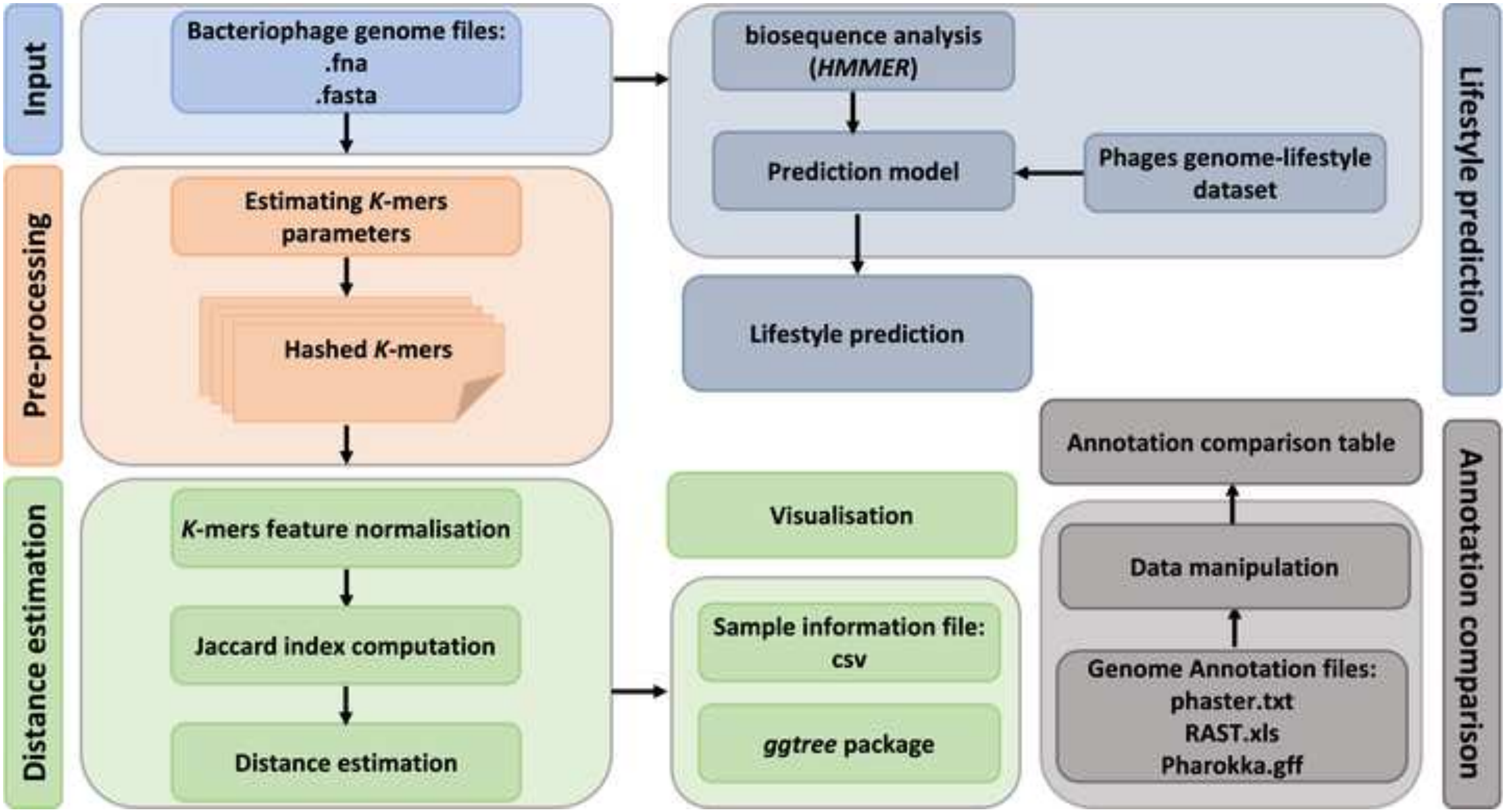

Figure 2

Click here to access/download;Figure;Figure 2.jpg

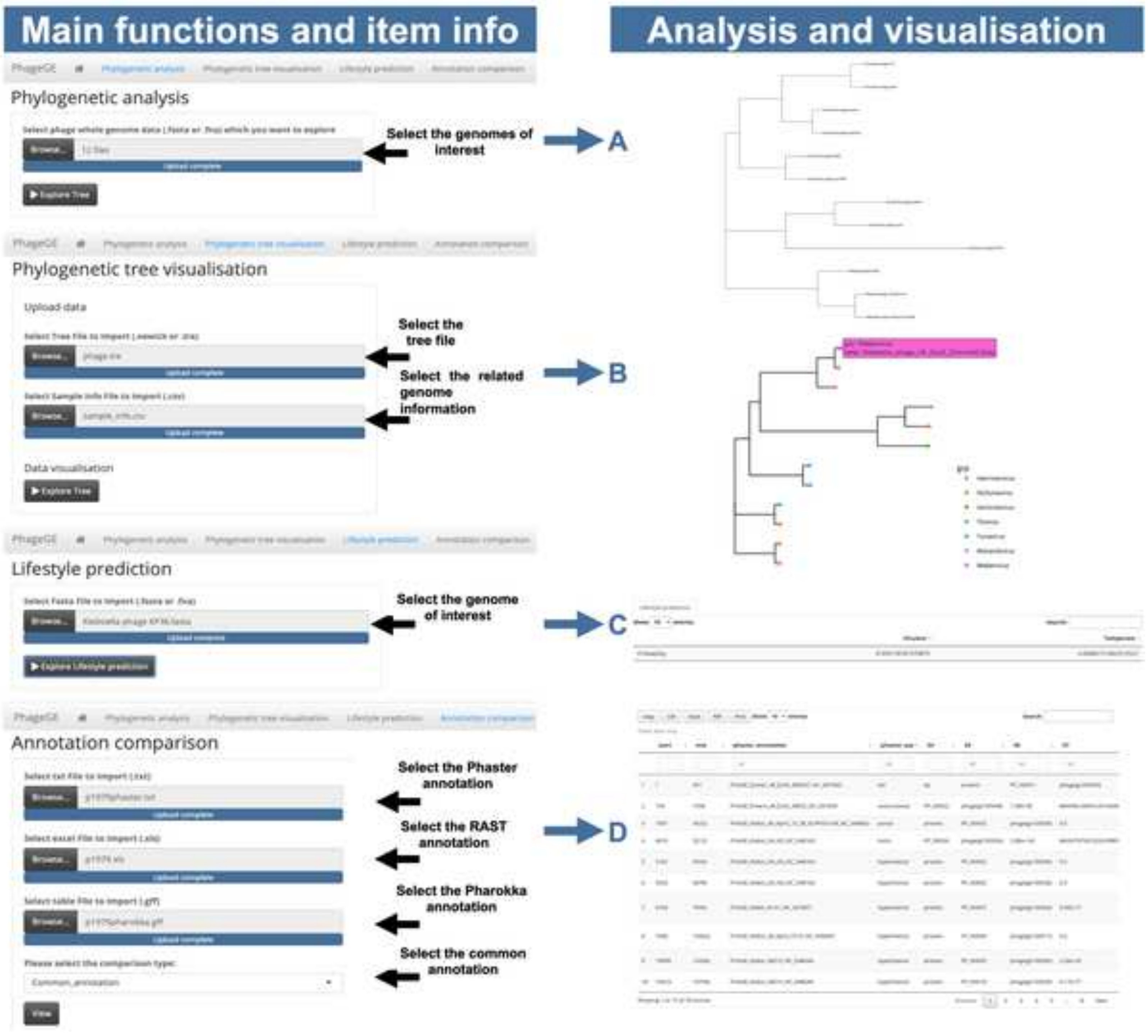

Figure 3

Click here to access/download;Figure;Figure 3.jpg

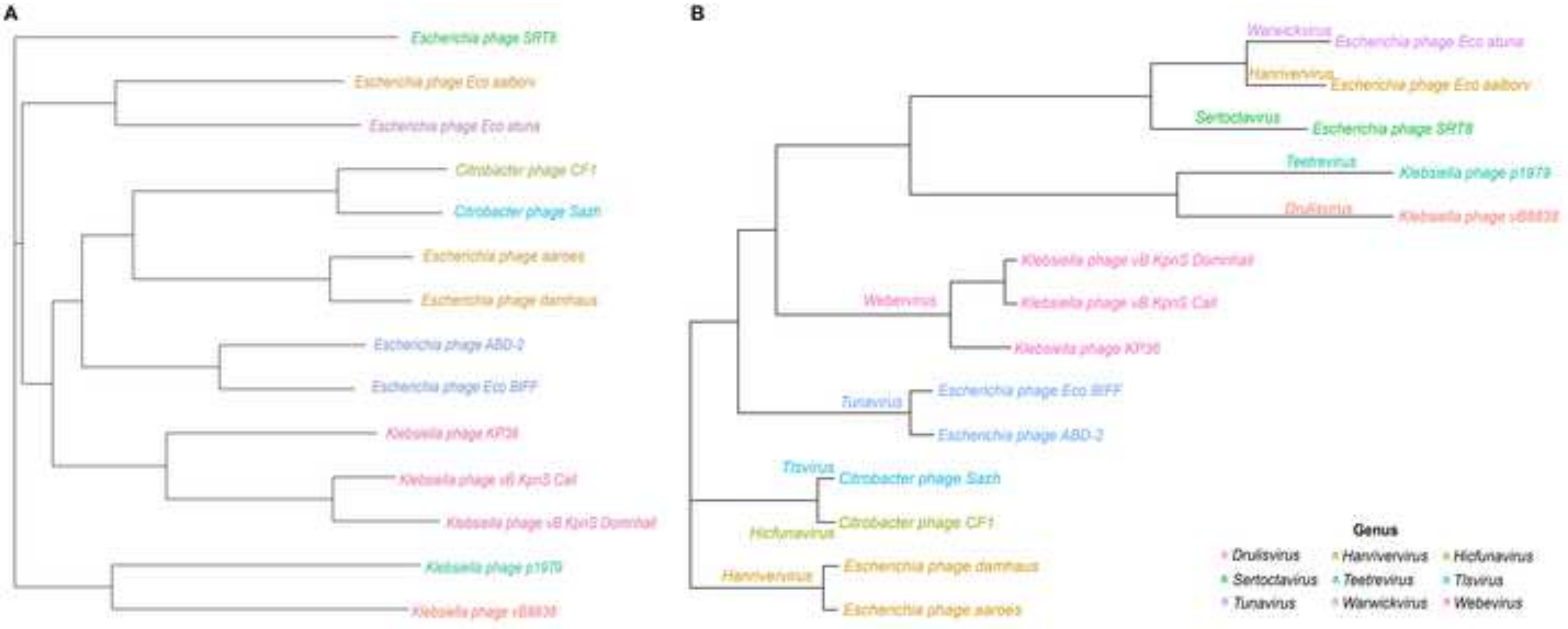

Figure 4

Click here to access/download;Figure;Figure 4.jpg

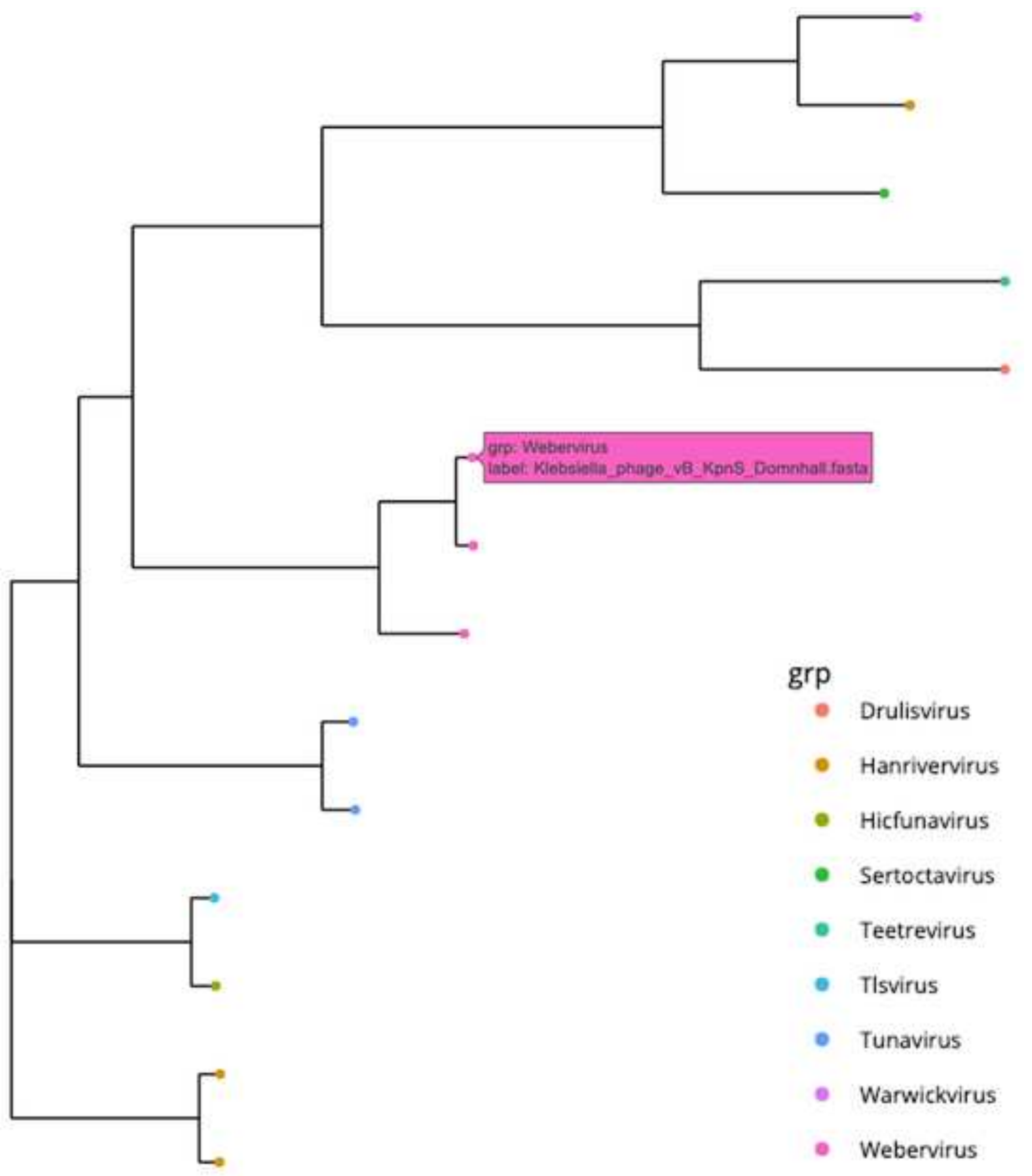

Figure 5

Click here to access/download;Figure;Figure 5.jpg

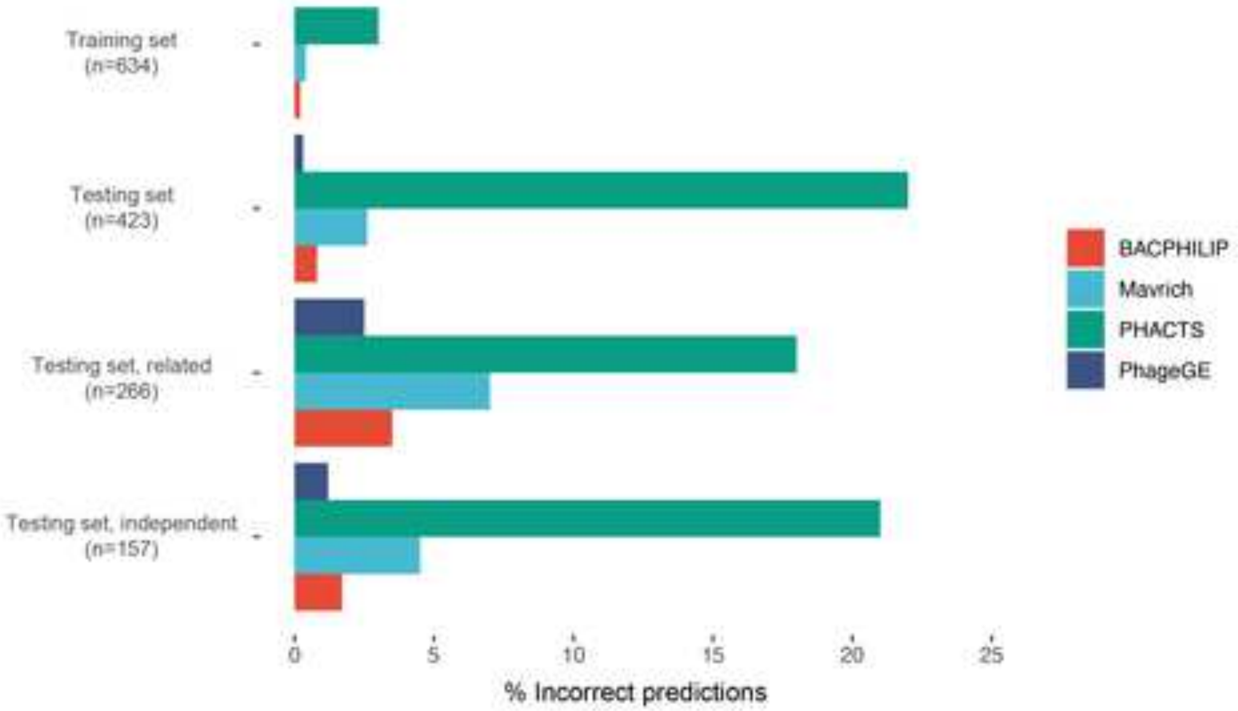

Personal Cover
Click here to access/download;Personal Cover;Cover letter.docx

MONASH University

Professor Jian Li
Fellow of the Australian Academy of Science
Fellow of the American Academy of Microbiology
Fellow of the Australian Academy of Health and Medical Sciences
Head, Laboratory of Antimicrobial Systems Pharmacology
Monash Biomedicine Discovery Institute

Dr Scott Edmunds

GigaScience

5th February 2024

Dear Dr Edmunds:

We are delighted to submit our manuscript entitled "*PhageGE: An interactive web platform for exploratory analysis and visualisation of bacteriophage genomes*" as a technical note. We believe that the developed tool from this study fits well in the scope of *GigaScience* and will be of interest to its readership.

Antibiotic resistance has become one of the greatest threats to human health globally. It is highlighted by the World Health Organization as an urgent issue requiring worldwide collaboration. With the drying antibiotic discovery pipeline, bacteriophages (phages) have been investigated as a potential therapeutic alternative against life-threatening bacterial infections. To optimise the phage therapy in patients, key pharmacological information is usually required, including infection cycle, genomics, and phage taxonomy. Phage genomic features play an important role in its pharmacology; however, **our knowledge of phage genomics is sparse and the use of existing bioinformatic tools requires considerable bioinformatic expertise**. These challenges have substantially limited the clinical translation of phage therapy.

Therefore, in the present study we developed an easy-to-use graphical user interface webserver PhageGE (Phage Genome Explorer) for investigations of phage genomics (e.g., phylogeny, lifestyle and gene contents). PhageGE can not only provide more functionalities for phage genomics analysis (e.g., phylogeny estimation, phylogenetic tree visualisation, lifestyle prediction and comparison of genome annotation) within one single tool, but also generate publication-ready analysis output. Within PhageGE, we implemented a phylogeny estimation function that applies an alignment-free genome comparison with feature frequency profiles derived from phage whole-genome sequences. Additionally, PhageGE incorporates *ggtree* to enable interactive and informative visualisation of phylogenetic trees. The lifestyle prediction function of PhageGE utilises a machine-learning approach to extract gene patterns from temperate phage genomes and build a lifestyle prediction model, providing new insights for further investigation of lifestyle on the basis of phage whole genomes. The overall approaches to both phylogenetic analysis and lifestyle prediction demonstrate that analyses results from PhageGE are comparable to previously published tools, indicating its effectiveness in accurately analysing phage phylogeny and predicting phage lifestyle. Moreover, the annotation comparison function of PhageGE facilitates efficient conversions of genome annotation files derived from different annotation pipelines. In summary, PhageGE is uniquely designed to enhance the efficiency of phage genomic analysis, offering a streamlined and optimised platform for phage biologists and pharmacologists.

We would suggest the following potential reviewers and do not have any conflict of interest with them.
Professor Balachandran Manavalan, Department of Physiology, Ajou University School of Medicine (bala@ajou.ac.kr)

19 Innovation Walk
Monash University
VIC 3800, Australia
Telephone: (+61 3) 990 39702 Facsimile: (+61 3) 990 56450 Email: Jian.Li@monash.edu
Web: https://www.monash.edu/discovery-institute/jian-li-lab

Unintended recipient: please notify as soon as possible and destroy all pages received

MONASH University

Professor Jian Li
Fellow of the Australian Academy of Science
Fellow of the American Academy of Microbiology
Fellow of the Australian Academy of Health and Medical Sciences
Head, Laboratory of Antimicrobial Systems Pharmacology
Monash Biomedicine Discovery Institute

Professor Wenming Zhao. Beijing Institute of Genomics, Chinese Academy of Sciences (zhaowm@big.ac.cn)
Professor Xiaoting Hua. Sir Run Run Shaw Hospital, Zhejiang University (xiaotinghua@zju.edu.cn)

We confirm that this manuscript has not been published elsewhere, nor has it been submitted to any other journals. All co-authors have agreed on the contents of the manuscript and there is no conflict of interest. Funding sources are provided in the Acknowledgements.

Thank you for considering our manuscript and we look forward to hearing from you soon.

Yours sincerely,

Jian Li PhD

19 Innovation Walk
Monash University
VIC 3800, Australia
Telephone: (+61 3) 990 39702 Facsimile: (+61 3) 990 56450 Email: Jian.Li@monash.edu
Web: https://www.monash.edu/discovery-institute/jian-li-lab

Unintended recipient: please notify as soon as possible and destroy all pages received
