# Supplementary material for: PhageGE: an interactive web platform for exploratory analysis and visualization of bacteriophage genomes
Source: Gigascience. 2024 Sep 25;13:giae074. doi: 10.1093/gigascience/giae074 (PMC11423353; doi:10.1093/gigascience/giae074)
Supplement: giae074_GIGA-D-24-00040_Revision_1 [file giae074_giga-d-24-00040_revision_1.pdf]

GigaScience

PhageGE: An interactive web platform for exploratory analysis and visualisation of bacteriophage genomes

--Manuscript Draft--

Manuscript Number:	GIGA-D-24-00040R1
Full Title:	PhageGE: An interactive web platform for exploratory analysis and visualisation of bacteriophage genomes
Article Type:	Technical Note
Funding Information:	Division of Microbiology and Infectious Diseases, National Institute of Allergy and Infectious Diseases (R21 AI156766) — Prof Jian Li
Abstract:	Background: Antimicrobial resistance is a serious threat to global health. Due to the stagnant antibiotic discovery pipeline, bacteriophages (phages) have been proposed as an alternative therapy for the treatment of infections caused by multidrug-resistant (MDR) pathogens. Genomic features play an important role in phage pharmacology. However, our knowledge of phage genomics is sparse and the use of existing bioinformatic pipelines and tools requires considerable bioinformatic expertise. These challenges have substantially limited the clinical translation of phage therapy. Findings: A user-friendly graphical interface application, PhageGE (Phage Genome Explorer), was developed for the interactive analysis of phage genomes. The new R Shiny webserver, PhageGE, was designed for analysing phage whole-genome sequence (WGS) data. PhageGE integrates several existing R packages and combines them with several newly developed functions to perform phylogeny analysis and lifestyle prediction. The webserver offers several additional key functions, including interactive phylogenetic tree visualisation and annotation comparison. The output from PhageGE can be exported directly with publication-quality images.<br>Conclusions: PhageGE is a valuable tool for analysing phage genome data and may expedite the development and clinical translation of phage therapy. PhageGE is publicly available at http://phagege.com/.
Corresponding Author:	Jian Li<br>Monash Biomedicine Discovery Institute<br>AUSTRALIA
Corresponding Author Secondary Information:	
Corresponding Author's Institution:	Monash Biomedicine Discovery Institute
Corresponding Author's Secondary Institution:	
First Author:	Jinxin Zhao, Ph.D.
First Author Secondary Information:	
Order of Authors:	Jinxin Zhao, Ph.D.
	Jiru Han, Ph.D.
	Yu-Wei Lin, Ph.D.
	Yan Zhu, Ph.D.
	Michael Aichem
	Dimitar Garkov
	Phillip J. Bergen, Ph.D.
	Sue C. Nang, Ph.D.
	Jian-Zhong Ye, Ph.D.

Powered by Editorial Manager® and ProduXion Manager® from Aries Systems Corporation

	Tieli Zhou, Ph.D.
	Tony Velkov, Ph.D.
	Jiangning Song, Ph.D.
	Falk Schreiber, Ph.D.
	Jian Li, Ph.D.
**Order of Authors Secondary Information:**	
**Response to Reviewers:**	Point-by-point responses<br><br>Editor comments:<br>1.Please register any new software application in the bio.tools and SciCrunch.org databases to receive RRID (Research Resource Identification Initiative ID) and biotoolsID identifiers, and include these in your manuscript. Computational workflows should be registered in workflowhub.eu and the DOIs cited in the relevant places in the manuscript. These will facilitate tracking, reproducibility and re-use of your tool.<br>Response: We have registered our application in bio.tools and SciCrunch.org databases and included the biotoolsID (biotools:phagege) and RRID (SCR_025380) in the revised manuscript (line 101).<br><br>Reviewers' comments:<br>Reviewer1:<br>The authors report here a new web-based tool called Phage Genome Explorer (PhageGE) for the interactive analysis of phage genomic data, which facilitates phylogenetic analysis and visualisation, the prediction of lytic vs., lysogenic lifestyles, and the interrogation of data generated by genome annotation tools (e.g., Pharokka). I commend the authors for developing this user-friendly tool that allows for greater access to non-experts. I believe this tool will have utility across clinical research and basic phage biology. I've tested the tool using both author supplied test data and data I've generated, and I have no major comments about the results and usability of PhageGE. However, I believe additional revisions are needed to strengthen the overall manuscript.<br><br>1.I would like to see the option to upload multi-fasta files implemented as a means to streamline usability. I think this can be implemented for both "phylogenetic analysis" and "lifestyle prediction" sections.<br>Response: We thank the reviewer for the suggestion and especially for providing the code for implementing multi-fasta format in our tools. We have incorporated the multi-fasta format into the "Phylogenetic analysis" function and revised the related description in the manuscript (lines 128-130). We have updated the previous "Lifestyle prediction" function for predicting multiple phage genomes simultaneously.<br><br>2.How does PhageGE scale to large metagenomic datasets? Unfortunately, I was unable to test this without the multi-fasta input option. However, I think it could scale nicely, especially with a circular tree format.<br>Response: We thank the reviewer for the suggestion. We have updated phageGE with a multi-fasta format input option and also provided an option for the final tree format (e.g., rectangular and circular format) (lines 128-132). We would like to clarify that the primary aim of PhageGE is to analyse phage genomic data, assuming that users already have assembled phage genomes rather than detecting them directly from large metagenomic datasets. This focus allows us to provide a robust and efficient tool specifically tailored for phage genome analysis. We apologise for any confusion this may have caused. The detection of phage sequences directly from large metagenomic datasets is beyond the current scope of PhageGE. Nevertheless, we acknowledge its importance and will consider developing this functionality in the next version of PhageGE.<br><br>3.Viral clusters have been shown to be important in determining viral diversity, and I think it would be a useful addition to the phylogenetic-based analyses. c.f., Camarillo-Guerrero et al., 2021. PMID: 33606979 and rBlast https://github.com/mhahsler/rBLAST<br>Response: We agree that viral clusters play a crucial role in determining viral diversity, as highlighted by Camarillo-Guerrero et al., and we appreciate the reference to rBlast as a valuable tool in this context. However, the primary aim of PhageGE is to serve as

a user-friendly web tool for rapid phylogenetic analysis and lifestyle prediction, particularly catering to users with limited programming experience. Additionally, PhageGE is designed to accelerate the translation of phage therapy into the clinic by providing phage phylogenetic and lifestyle information. As such, we have focused on providing an accessible and efficient platform for these specific purposes. While the inclusion of viral cluster analysis is beyond the current scope of PhageGE, we recognise its importance and potential benefits and will consider incorporating this feature in the next version of PhageGE.

4.On the "Phylogenetic analysis" landing page, I think "select phage whole genome data" should read "select phage genome data" as whole genome data would imply that phage particles were isolated and sequenced.
Response: We apologise for any confusion caused by the terminology on the "Phylogenetic analysis" landing page. We understand that "whole genome data" implies that phages were isolated and sequenced. To clarify, the primary function of PhageGE is to analyse assembled phage genomic data, which should use “phage whole-genome data” in the landing page as well as the usage description. To prevent any further misunderstanding, we have updated the description for PhageGE: "To demonstrate the functions and the scope of application of PhageGE, we herein describe the results of a case study using PhageGE, including phage whole-genome data (i.e., .fasta), a phylogenetic tree file (i.e., .tre), and genome annotation data (i.e., .xls, .txt and .gff), collectively referred to as “Example Data” (Figure 1)." (lines 105-108).

5."This demonstrates that the phylogenetic analysis performance of PhageGE is accurate and comparable to the multiple sequence alignment-based approach." And "It has demonstrated the ability to accurately reconstruct biologically relevant phylogenies with thousands of microbial genomes [40-42]. The description of this function is briefly outlined below." How do phylogenies obtained using whole phage genomes (k-mer, ANI, or otherwise) compare to those reconstructed using the large terminase gene?
Response: We thank the reviewer for the insightful question regarding the comparison between phylogenies obtained from PhageGE and those reconstructed using the large terminase gene. Although both phylogeny analyses from whole phage genomes (k-mer based) and the large terminase gene can provide insights into phage diversity and evolution, there is a distinction. Whole-genome based analysis utilises the entire genomic content, capturing the full extent of genetic variation across the genome; while phylogeny reconstructed using a single gene (i.e. the large terminase gene) provides a narrower view of the phage's evolutionary history and potentially misses some genetic variations present. Furthermore, phages have the capability to lose or duplicate genes, including the large terminase gene, potentially leading to inaccuracies in phylogenetic inference (Nat. Microbiol., 2017, 2(9), 1-9; Nat. Rev. Microbiol., 2021, 15(3), 161-168). In contrast, k-mer based whole-genome phylogenies offer a comprehensive and high-resolution view of phage relationships, particularly valuable in distinguishing closely related phages and providing a more holistic view of their evolutionary relationships (mBio, 2017, 8(4), 10-1128). Therefore, we integrated a k-mer based whole phage genome phylogenetic analysis function into PhageGE to provide a high-resolution view of phage phylogeny for clinical translation.

6."Furthermore, combining whole-genome sequencing (WGS) with in silico prediction enables rapid prediction of phage lifestyle [18]. Several popular bioinformatic pipelines and tools are available for such analyses, including MAFFT, RAxML and IQ-TREE (for multiple sequence alignment and phylogenetic analysis) [19-21], ggtree (for the visualisation of phylogeny data) [22], PHACTS and BACPHLIP (for phage lifestyle prediction) [18, 23]." What do each of the programs do? Perhaps restructure writing to reflect programs at higher-order groups. e.g., Several popular bioinformatic pipelines and tools are available for multiple sequence alignment (MAFFT), phylogenetic reconstruction (RAxML, IQ-TREE), visualisation of phylogeny (ggtree), and for phage lifestyle prediction (PHACTS, BACPHLIP).
Response: We thank the reviewer for the suggestion. The sentence has been restructured accordingly (lines 85-91).

7."However, utilising these tools requires proficient programming skills, therefore, a biologist-friendly pipeline for phage genomic analyses is urgently needed to address the aforementioned limitations in phage genomic analysis." Its not entirely clear what

Powered by Editorial Manager® and ProduXion Manager® from Aries Systems Corporation

the aforementioned limitations are. Are you referring to: "Optimising phage therapy in patients requires key pharmacological information, including infection cycle, gene content and phage taxonomy"
Response: The limitations refer to proficient programming skills required for phage genomic analysis when using these tools. We have clarified this point in the revised manuscript (lines 88-91).

General editorial revisions are required, some examples are given below:
Response: We thank the reviewer for the suggestions. In addition to the general editorial revisions suggested by the reviewer below, we have substantially revised the manuscript to improve grammar. Minor changes were not highlighted.

8."To demonstrate the functions and application scope of PhageGE"
To demonstrate the functions and the scope of application of PhageGE
Response: The sentence has been revised accordingly (line 105).

9."This demonstrates that the phylogenetic analysis performance of PhageGE is accurate and comparable to the multiple sequence alignment-based approach."
This demonstrates that the performance of the phylogenetic analysis of PhageGE is accurate and comparable to the multiple sequence alignment-based approach.
Response: The sentence has been revised accordingly (lines 142-144).

10."Respectively" is used too frequently and creates confusing sentence constructions. e.g., "By selecting "common_annotation", a table with 75, 45, 51 genes that were annotated in all three pipelines were generated for KP36, vB8838 and FK1979, respectively. We also identified 17, 7 and 12 unique genes, respectively, from the Pharokka pipeline by selecting "Pharokka_only" option."
Response: We thank the reviewer for the suggestion. The second sentence above has been rewritten (lines 194-195).

11."By employing an improved searching function (i.e. searching a sequence file against the build-in HMM [Hidden Markov Model] database)"
By employing an improved search function (i.e. searching a sequence file against the built-in HMM [Hidden Markov Model] database)"
Response: The manuscript has been revised accordingly (line 323).

12."To illustrate the phylogenetic analysis function in PhageGE, we employed our GitHub example dataset which consists of 14 phage genomes (Citrobacter, Escherichia, and Klebsiella) from 9 different genera (Figure 2A)."
Need to make clear what the link between the 14 phage genomes to Citrobacter, Escherichia, and Klebsiella are. Are they 14 genomes of lytic phages that target Citrobacter, Escherichia, and Klebsiella? Or are they 14 phage sequences/genomes detected from bacterial isolate genomes of Citrobacter, Escherichia, and Klebsiella? I think a section describing the origin of data used would be helpful for readers.
Response: We thank the reviewer for the suggestion and have revised the manuscript accordingly (lines 112-121). All 15 phages are lytic phages that target Citrobacter freundiifreundii (2 phages), Escherichia coli (7 phages), and Klebsiella pneumoniae (6 phages).
These 15 phage genomes were selected to demonstrate the application of PhageGE to a wide range of phages targeting clinically relevant pathogens. We included a K. pneumoniae phage, pKp20, and performed the phylogenetic analysis for this phage along with the other 14 phages. Notably, the taxonomic and lifestyle results of pKp20 contributed to a recent successful clinical case (Antimicrob. Agents Chemother., 2023, 67(4), e00037-23).

13."To compare the results obtained from PhageGE with the multiple sequence alignment-based approach, we also conducted a multiple sequence alignment-based phylogenetic analysis using MAFFT v7.47 alongside the phylogenetic analysis conducted in PhageGE"
What is the first MSA-based approached referring to here? I think the results section requires a brief overview of the steps executed within PhageGE to orientate the readers. This would provide a baseline understanding in an effort to facilitate the comparative narrative.
Response: We have revised the manuscript to clarify this point (lines 126-133). The

	MSA-based approach here refers to the phylogenetic analysis using MAFFT v7.47 and fasttree v2.1.10. We have also included a brief discussion on the performance of PhageGE in phylogenetic analysis with uploaded phage genomes.<br><br>14."Its aim is to provide an interactive visualisation platform that improves the reusability of phylogenetic data and facilitates the phylogenetic analysis of phage comparative genomics studies." Reusability = reproducibility?<br>Response: This sentence has been changed to "...interactive visualisation platform that enhances the accessibility of phylogenetic data..." (line 147).<br><br>15."Overall, all four functions from PhageGE serve as a guide for the exploration of phage genomic features and will expedite the clinical translation of phage therapy." The test data set requires more phage genomes that serve as positive and negative controls, including eukaryotic viruses. Table 2 phage lifecycle prediction needs controls for temperate phages, and non-phage viruses.<br>Response: We thank the reviewer for the suggestion and have included more phages (e.g. temperate phages) in the lifestyle prediction table (Table 2) to serve as positive (e.g. KP36 and pkp20) and negative (e.g. NC_017985 and NC_027339) controls (lines 176-180). Regarding the inclusion of eukaryotic viruses, PhageGE is for genomic analyses of phages specifically, not non-phage viruses. We have also updated our current function to pop up an error message when non-phage viruses are detected: "The input is not from phage viruses".<br><br>16.Figure legends require more descriptive text in order to assess.<br>Response: We thank the reviewer for the suggestion and have improved the figure legends accordingly.<br><br>17.Image quality of figures needs improvement, especially figure 5.<br>Response: All figures have been updated with a resolution of 300 dpi or higher.<br><br>18.Last sentence of first paragraph - upton = upon; Second paragraph - multi-omics has* the<br>Response: We apologise for the typographic errors and the manuscript has been revised accordingly (lines 78 and 80).<br><br>Reviewer2:<br>Major points:<br>1.It was seen that various annotation tools have been developed for phage genomes, and there are several works developed as integrated tools or pipelines for phage genome annotation and visualization. For example, Prophage Hunter (Song et al. 2019), Galaxy and Apollo (Ramsey et al. 2020), PhaGAA (Wu et al. 2023), … et al. However, the authors did not mention and discuss those works. Compared with those published works, PhageGE was designed with its functions some different from them, but still limited for the research community.<br>Response: We thank the reviewer for the comments regarding the comparison of PhageGE with other phage genome annotation and visualisation tools. In the revised manuscript we have clarified that PhageGE serves as a biologist-friendly interactive platform for phage genome analysis with a particular emphasis on phylogeny, lifestyle prediction, interactive phylogenetic tree visualisation, and annotation comparison (lines 92-98). The interactive visualisation capabilities of PhageGE are tailored to improve the accessibility and usability of phylogenetic data, facilitating comparative genomics studies and clinical translation within the phage research community.<br>Prophage Hunter is for studying active phages from whole genome assemblies of bacteria. The functionalities of PhageGE are designed to complement, rather than replicate, the capabilities of tools like Prophage Hunter.<br>The main annotation pipeline used in Galaxy and Apollo is PHANOTATE, which has been adapted into the Pharokka pipeline (Bioinformatics, 2023, 39(1), p.btac776). PhageGE focuses on integrating annotations into an interactive environment for comparative genome analysis and visualisation. Our approach enhances the utility of the annotations by providing a platform for deeper exploration and interpretation of phylogenetic relationships.<br>PhaGAA is an excellent online integrated platform for phage genome annotation and analysis, focusing on DNA/protein-based annotation, host prediction, and lifestyle reorganisation. The lifestyle reorganisation method in PhaGAA directly integrates

Powered by Editorial Manager® and ProduXion Manager® from Aries Systems Corporation

PhaTYP (Brief. Bioinform., 2023, 24(1), p.bbac487). The primary utility of PhaTYP is analysing phage lifestyle in human neonates' gut data, showcasing its value in studying phages in metagenomic contexts and enhancing our understanding of microbial communities.
In summary, PhageGE offers unique functionalities that complement existing tools, focusing on providing a biologist-friendly and specialised environment for phage genome analysis.

2.As pointed out above, PhageGE's functions were not comprehensive enough, especially did not address the characteristics of the host of bacteriophage or phage-host interaction which are important for phage genome studies. In addition, currently a tool like PhageGE would be expected to analyze metagenomic data with a large of short reads. Moreover, identification of resistance genes, analyzing potentially encoded resistance genes within the phage genome is crucial in phage genome analysis. So, adding analysis function of antibiotic resistance gene dissemination, examining genes related to antibiotic resistance in the phage genome, especially those that might affect host bacterial resistance through horizontal gene transfer, could greatly enhance the understanding of bacteriophages, their evolution, and host interactions if these analytical functions were integrated into the PhageGE pipeline.
Response: We appreciate the reviewer's valuable suggestions for enhancing PhageGE. We agree that understanding host characteristics and phage-host interactions are crucial; however, they are beyond the current scope of PhageGE. As mentioned in our response to Comment #1 above, PhageGE focuses on phylogenetic analysis and lifestyle prediction, aiming to expedite clinical translation of phage therapy (lines 116-121 and 176-177). This focus has led to a successful clinical case study (Antimicrob. Agents Chemother., 2023, 67(4), e00037-23).
Regarding antibiotic resistance gene (ARG) analysis, we recognise its critical role in understanding phage biology and their potential impact on bacterial resistance through horizontal gene transfer. Notably, recent studies have demonstrated that phages and prophages rarely carry ARGs, and bona fide ARGs attributed to phages in human- or mouse-associated viromes were previously overestimated due to bacterial DNA contamination and relaxed detection thresholds, leading to high false-positive rates (ISME, 2017, 11(1), 237-247; ISME Commun., 2021, 1(1), 55). Nonetheless, we will consider incorporating this function in future versions of PhageGE.

3.As a presentation of an application, the authors provided limited cases with example datasets, and limited analysis.
Response: We thank the reviewer for the suggestion. In the revised manuscript we have included more example datasets to demonstrate each function (e.g., phylogenetic analysis and lifestyle prediction) (lines 112-121, 137-144, and 176-180). Moreover, we have demonstrated the application of functions from PhageGE using a clinical case study (lines 116-121 and 177-180).

Minor points:
4.The authors highlight in the background section the role of phage genome analysis in developing phage therapies. Therefore, it would be beneficial to demonstrate the application of this tool in case studies.
Response: We thank the reviewer for the suggestion. The manuscript has been revised to include a clinical case study (Antimicrob. Agents Chemother., 2023, 67(4), e00037-23) which demonstrates the application of phageGE (lines 112-121 and 176-180). This case study involved a recurrent urinary tract infection, and both taxonomy information from phylogeny analysis and the lifestyle prediction had played key roles in the phage selection.

5.While many offline tools for constructing phage evolutionary trees have been developed, a major disadvantage of a web tool is its lengthy runtime. The capacity of the tool to process a significant number of sequence data and the need for a runtime comparison should be addressed.
Response: We thank the reviewer for the suggestion. In the revised version we have included a comparison of the PhageGE runtime with the MSA-based approach (lines 138-144). On a 2-GHz CPU with 64 GB RAM, PhageGE performed phylogenetic analysis for 15 and 146 phage genomes in 0.22 minutes and 4.42 minutes, respectively. In comparison, the MAS-based approach required more than 30 minutes and 296 minutes accordingly. Therefore, PhageGE offers superior computational and

	analysis efficiency.<br><br>6.The image resolution is too low, at only 144 dpi, insufficient for the required 300 dpi. Many characters in Figure 2A are unclear, suggesting a need for improved resolution.<br>Response: As per Reviewer 1, Point 17, all figures have been updated with a resolution of 300 dpi or higher.<br><br>7.The website http://phagege.com/ is not functioning and cannot be accessed.<br>Response: We have retested our current version and the url works properly.
**Additional Information:**	
**Question**	**Response**
Are you submitting this manuscript to a special series or article collection?	No
**Experimental design and statistics**<br><br>Full details of the experimental design and statistical methods used should be given in the Methods section, as detailed in our Minimum Standards Reporting Checklist. Information essential to interpreting the data presented should be made available in the figure legends.<br><br>Have you included all the information requested in your manuscript?	Yes
**Resources**<br><br>A description of all resources used, including antibodies, cell lines, animals and software tools, with enough information to allow them to be uniquely identified, should be included in the Methods section. Authors are strongly encouraged to cite Research Resource Identifiers (RRIDs) for antibodies, model organisms and tools, where possible.<br><br>Have you included the information requested as detailed in our Minimum Standards Reporting Checklist?	Yes
**Availability of data and materials**<br><br>All datasets and code on which the conclusions of the paper rely must be	Yes

either included in your submission or deposited in publicly available repositories (where available and ethically appropriate), referencing such data using a unique identifier in the references and in the “Availability of Data and Materials” section of your manuscript.<br><br>Have you have met the above requirement as detailed in our Minimum Standards Reporting Checklist?	

Powered by Editorial Manager® and ProduXion Manager® from Aries Systems Corporation

Manuscript_highlighted_changes Click here to access/download;Manuscript;GIGA-D-24-00040 PhageGE_main.docx

PhageGE: An interactive web platform for exploratory analysis and visualisation of bacteriophage genomes

Jinxin Zhao[1, 2*], Jiru Han[3], Yu-Wei Lin[1], Yan Zhu[1, 4], Michael Aichem[5], Dimitar Garkov[5], Phillip J. Bergen[1], Sue C. Nang[1], Jian-Zhong Ye[6, 7], Tieli Zhou[6, 7], Tony Velkov[8], Jiangning Song[2, 9], Falk Schreiber[5, 10], Jian Li[1, 2*]

[1] Infection Program and Department of Microbiology, Biomedicine Discovery Institute, Monash University, Clayton, VIC, Australia

[2] Monash Biomedicine Discovery Institute-Wenzhou Medical University Alliance in Clinical and Experimental Biomedicine, Monash University, Clayton, VIC, Australia

[3] Population Health and Immunity Division, The Walter and Eliza Hall Institute of Medical Research, Parkville, VIC, Australia

[4] Systems Biology Center, Tianjin Institute of Industrial Biotechnology, Chinese Academy of Sciences, Tianjin, China

[5] Department of Computer and Information Science, University of Konstanz, Konstanz, Germany

[6] Key Laboratory of Clinical Laboratory Diagnosis and Translational Research of Zhejiang Province, Department of Clinical Laboratory, The First Affiliated Hospital of Wenzhou Medical University, Zhejiang, China

[7] Wenzhou Medical University-Monash Biomedicine Discovery Institute Alliance in Clinical and Experimental Biomedicine, The First Affiliated Hospital of Wenzhou Medical University,Wenzhou, China

[8] Department of Pharmacology, Biomedicine Discovery Institute, Monash University, Melbourne, VIC, Australia

[9] Department of Biochemistry and Molecular Biology, Biomedicine Discovery Institute, Monash University, Melbourne, VIC, Australia

[10] Faculty of Information Technology, Monash University, Clayton, VIC, Australia

Email addresses: JZ, jinxin.zhao@monash.edu; JH, han.ji@wehi.edu.au; YL, yu-wei.lin@monash.edu; YZ, Yan.Zhu@monash.edu; MA, michael.aichem@uni-konstanz.de; DG, dimitar.garkov@uni-konstanz.de; PB, phillip.bergen@monash.edu; SN, sue.nang@monash.edu; JY, jzye89@163.com; TZ, wyztli@163.com; TV, tony.velkov@monash.edu; JS, jiangning.song@monash.edu; FS, falk.schreiber@uni-konstanz.de; JL, jian.li@monash.edu.

Running title: PhageGE for bacteriophage genomic analysis

*Corresponding authors:
Dr Jinxin Zhao, Tel: +61 3 99056288, Email: jinxin.zhao@monash.edu;
Professor Jian Li, Tel: +61 3 99039172 Fax: +61 0399056450; Email: jian.li@monash.edu.

Abstract

Background: Antimicrobial resistance is a serious threat to global health. Due to the stagnant antibiotic discovery pipeline, bacteriophages (phages) have been proposed as an alternative therapy for the treatment of infections caused by multidrug-resistant (MDR) pathogens. Genomic features play an important role in phage pharmacology. However, our knowledge of phage genomics is sparse and the use of existing bioinformatic pipelines and tools requires considerable bioinformatic expertise. These challenges have substantially limited the clinical translation of phage therapy. **Findings:** A user-friendly graphical interface application, PhageGE (Phage Genome Explorer), was developed for the interactive analysis of phage genomes. The new R Shiny webserver, PhageGE, was designed for analysing phage whole-genome sequence (WGS) data. PhageGE integrates several existing R packages and combines them with several newly developed functions to perform phylogeny analysis and lifestyle prediction. The webserver offers several additional key functions, including interactive phylogenetic tree visualisation and annotation comparison. The output from PhageGE can be exported directly with publication-quality images.

Conclusions: PhageGE is a valuable tool for analysing phage genome data and may expedite the development and clinical translation of phage therapy. PhageGE is publicly available at http://phagege.com/.

Keywords: Phage genome, biological web application, genomic analysis, phylogeny, lifestyle

Introduction

The rapid emergence and spread of antimicrobial resistance (AMR) is one of the three greatest threats to human health globally [1]. It is estimated that by 2050, life-threatening infections caused by antimicrobial-resistant pathogens will kill more people than any other diseases [2]. Of particular concern is the increased prevalence of infections caused by Gram-negative pathogens, which are more difficult to treat than Gram-positive pathogens [3]. Given the sluggish global antibiotic pipeline [4], bacteriophages (phages) have attracted significant attention over the last decade as a potential alternative therapy for bacterial infections [5]. Phages are bacterial viruses and the advantages of phage therapy over antibiotics include a narrow spectrum of activity, the capacity to multiply at the infection site, and safety [6-8]. Optimising phage therapy in patients requires key pharmacological information, including infection cycle, gene content, and phage taxonomy [9, 10]. For example, temperate phages do not immediately lyse bacterial host cells and have an inherent capacity to mediate the transfer of genes between bacteria, potentially facilitating increased bacterial virulence and AMR. In contrast, lytic phages kill bacteria upon infection and are commonly used for the treatment of MDR bacterial infections in patients [11-14].

Multi-omics has the potential to expedite the clinical translation of phage therapy for the treatment of MDR bacterial infections [15]. For example, whole genome-based phylogenetic analysis offers significant advantages in understanding phage evolutionary dynamics and designing potential phage cocktails [16, 17]. Furthermore, combining whole-genome sequencing (WGS) with *in silico* prediction enables rapid prediction of phage lifestyle [18]. Several popular bioinformatic pipelines and tools are available for multiple sequence alignment (MAFFT) [19], phylogenetic reconstruction (RAxML and IQ-TREE) [20, 21], visualisation of phylogeny (ggtree) [22], and phage

lifestyle prediction (PHACTS and BACPHLIP) [18, 23]; however, utilising these tools requires proficient programming skills. Therefore, a biologist-friendly platform for phage genomic analyses is urgently needed to overcome the challenges associated with the requirement for advanced programming expertise.

Here, we developed an integrated webserver platform, PhageGE, that offers four key functionalities: phage phylogenetic analysis, tree visualisation, lifestyle prediction, and manipulation of phage genome annotation datasets. PhageGE differs from existing phage genomic analysis tools in that it facilitates the seamless export of all associated results in a publication-ready format without requiring complex procedures or long running times. Overall, PhageGE provides a biologist-friendly interface to streamline phage genomic analysis with WGS data.

Results

The PhageGE webserver (biotoolsID: biotools:phagege and RRID: SCR_025380) was designed to ensure biologist-friendliness and compatibility with major web browsers, including Google Chrome, Mozilla Firefox, Apple Safari, and Microsoft Edge (**Table 1**).

Webserver submission and case studies

To demonstrate the functions and the scope of application of PhageGE, we herein describe the results of a case study using PhageGE, including phage whole genome data (i.e., .fasta), a phylogenetic tree file (i.e., .tre), and genome annotation data (i.e., .xls, .txt and .gff), which are collectively referred to as “Example Data” (**Figure 1**). The complete set of Example Data used in the case studies can be accessed on the PhageGE GitHub repository (https://github.com/JinxinMonash/PhageGE).

Phage phylogenetic analysis and visualisation

To illustrate the phylogenetic analysis function in PhageGE and its application in clinical translation, we analysed our GitHub example dataset, which consists of 15 phage genomes. The hosts of the 15 phage genomes in the phylogenetic analysis are from 3 different bacterial species: *Citrobacter freundii, Escherichia coli, and Klebsiella pneumoniae* (**Figure 2A**). This dataset includes one anti-Klebsiella phage, pKp20, which was isolated in our lab and used in a clinical case [24]. In that case, a recurrent urinary tract infection [rUTI] was successfully treated with 4 weeks of adjunctive intravenous bacteriophage therapy, with no recurrence during a year of follow-up [24]. Both taxonomy information from phylogeny analysis and the lifestyle prediction played key roles in the selection of pKp20 over a wide range of phages [24]. The phage WGS data in the .fna or .fasta format can be obtained either from NCBI or prepared locally using standard genome assembly pipelines (e.g., SPAdes) based on the previous BLASTn result [24]. To compare the results obtained from PhageGE with the multiple sequence alignment-based approach, we also conducted a multiple sequence alignment-based phylogenetic analysis using MAFFT v7.47 and fasttree v2.1.10, alongside the phylogenetic analysis using PhageGE. We firstly uploaded the selected fasta files or a multi-fasta file which contains all phage genomes on the Phylogenetic Analysis page in PhageGE, then selected the layout of the tree (i.e., phylogram, cladogram, fan, radial, or tidy) and clicked the "Explore Tree" icon. The resulting phylogenetic tree, representing the relationships among the uploaded genomes, was generated using the built-in *k*-mer-based alignment-free phylogenetic approach, as detailed in the Methods section (**Figures 2A and 3A**). To enhance the clarity, we manually highlighted the 15 phages with distinct colours according to their genus. Comparison of the phylogenetic trees generated by PhageGE and MAFFT revealed

that both trees shared largely the same classification (e.g., positions of each phage and the related taxa) (**Figure 3**). Moreover, PhageGE demonstrates a significant improvement in runtime efficiency. For example, on a 2-GHz CPU with 64 GB RAM server, the runtimes of generating phylogenetics trees by PhageGE were 0.22 minutes for 15 phage genomes and 4.42 minutes for 146 phage genomes. In contract, the MSA-based approach (using tools like MAFFT along with FastTree) took 30 minutes and 296 minutes, respectively. This demonstrates that the performance of the phylogenetic analysis of PhageGE is accurate, fast and comparable to the multiple sequence alignment-based approach.

The phylogenetic visualisation function handles the phylogenetic tree along with diverse accompanying data. Its aim is to provide an interactive visualisation platform that enhances the accessibility of phylogenetic data and facilitates the phylogenetic analysis of phage comparative genomics studies. The phylogenetic tree and associated data can be extracted using a built-in function within PhageGE. This function is illustrated using a tree file "phage.tre" obtained from phage phylogenetic analysis (whether generated by PhageGE or other phylogenetic analysis pipeline) and a sample information file named "sample_info.csv" containing the taxonomy information for all 14 phages (**Figure 2B**). As shown in **Figure 4**, each dot in the dendrogram represents one phage with the colour indicating its taxonomic classification in the same genus. In addition, detailed information of each phage (e.g., name and taxonomy) can be easily accessed by hovering the cursor over the dot of interest (as indicated by the pink box in **Figure 4**). This interactive feature allows users to dynamically integrate and visualise the underlying information in a user-friendly manner.

Performance of phage lifestyle prediction

The lifestyle prediction function builds on a Random Forest classifier that incorporates up-to-date conserved protein domains with the ability to classify temperate and lytic phages using WGS data. To evaluate its performance, we compared the function with other published tools using the dataset of 1,057 phages in the literature [25]. The PhageGE lifestyle prediction function achieved the lowest error rates (0%, 1.2%, 0.3% and 2.5%, equivalent to 100%, 98.8%, 99.7% and 97.5% classification accuracy, respectively) across all tested datasets, substantially outperforming those existing tools for phage lifestyle classification (**Figure 5**). The prediction accuracy of PhageGE exceeded that of the most accurate existing tool, BACPHLIP, which had prediction accuracies of 99.8%, 98.3%, 99.2% and 96.5%, respectively (**Figure 5**). Similarly, WGS data for individual phages (e.g., *Klebsiella* phage KP36.fasta, vB8388.fasta, and FK1979.fasta from the example dataset described here) can be uploaded as input to generate the phage lifestyle probability table (**Figure 2C** and **Table 2**). The result presented in **Table 2** predicts that *Klebsiella* phages KP36 (a model phage in our laboratory), FK1979, and vB8388 [26] (two phages isolated from hospital sewage, The First Affiliated Hospital of Wenzhou Medical University, China), and pKp20 (used in the rUTI clinical case study) [24], are highly likely lytic phages, with the probability of 99.3%, 95.6% and 96.9%, respectively. Meanwhile, the four phages from the NCBI in **Table 2** NC_017985, NC_027339, NC_009815, and NC_019768 are highly likely temperate phages. This function empowers users to rapidly analyse the lifestyle of a phage of interest *in silico* with high prediction accuracy, providing key insights into the intricate phage ecosystems and enabling optimal design of phage therapy.

Comparison of phage genome annotation

Notably, PhageGE also provides a function to compare phage genome annotations obtained from different pipelines (i.e., Pharokka, Phaster and RAST). This analysis

involves the integration of R package flextable, which allows for the generation of downloadable comparison results in multiple formats (e.g., csv, Excel and PDF). The user interface offers the flexibility to rank the results based on multiple parameters (e.g., location and/or length of the coding sequence [CDS]). In the case study presented here, we used PhageGE to compare genome annotations of *Klebsiella* phages KP36, vB8838, and FK1979 generated from Phaster, RAST, and Pharokka (**Figure 2D**). By selecting “common_annotation”, a table with 75, 45, and 51 genes that were annotated in all three pipelines was generated for KP36, vB8838, and FK1979, respectively. We also identified 17, 7, and 12 unique genes from the Pharokka pipeline by selecting the “Pharokka_only” option. To gain a better understanding of those unique annotated genes, PhageGE allows users to directly copy and download both the nucleotide and amino acid sequences associated with the genes from the interactive table. This feature facilitates further investigation of these unique annotations.

Discussion

With the dramatic rise in MDR bacterial infections, phage therapy has emerged as a safe and potentially effective alternative treatment option to antibiotics [27]. However, the development of effective phage therapies is complex, involving the isolation, culturing, characterisation, and timely preparation of efficacious phages. Traditionally, this process is time-consuming and costly [28, 29]. Nevertheless, with the next-generation sequencing techniques, it has become possible to rapidly and cost-effectively characterise phages. Despite this advancement, there is a paucity of intuitive tools available for phage genomics, with the majority requiring operation in

command-line mode. The availability of large phage genomic datasets presents unique opportunities to develop bioinformatics tools that aid in phage biology and pharmacology research. The use of computational methods to study phages has shown promise in generating novel insights, such as phylogeny and lifestyle, through bioinformatic analysis [18, 25, 30]. However, there is currently no single tool available that encompasses all those functions (e.g., phylogenetic analysis, tree visualisation, lifestyle prediction, and genome annotation comparison) in the webserver platform. Herein, we describe the development of the PhageGE webserver GUI streamlined for user-friendly phage genomic analysis.

PhageGE is a novel, biologist-friendly GUI application for the interactive analysis of phage genomes. The overarching goal of PhageGE is to provide an interactive analysis and visualisation platform for the rapid exploration of phage genomic associations, thereby promoting efficient genomic data-driven discovery of phage therapy. PhageGE comprises a set of functions for phage genomic analysis, including phylogenetic analysis, tree visualisation, lifestyle prediction, and genome annotation comparison. While current tools like PhaGAA can provide lifestyle reorganisation analysis, their primary utility lies in analysing phage lifestyle for their prefered phage dataset (e.g., gut flora of human neonates) [31]. In contrast, PhageGE integrates a more comprehenstive dataset with a wide range of phage genomes, allowing for broader and deeper exploration of phage lifestyles. Moreover, the comparsion of annotations from different pipelines highlights the key role of PhageGE in advancing phage genomics through enhanced analysis and visualisation functions. To exemplify the utility of PhageGE, we investigated the phylogeny, lifestyle, and annotation comparison of *Klebsiella* phages KP36, vB8838, and FK1979, which were independently isolated in two different countries. Our findings demonstrate that the

various functions of PhageGE yield comparable or better results than existing state-of-the-art approaches. These results highlight the significant potential of PhageGE in analysing various phage genomic features using phage WGS data.

Notably, PhageGE requires only phage WGS data as the input for conducting the related analysis. The phage phylogenetic analysis function takes phage WGS in the fasta format as input and applies an alignment-free phylogenetic approach to infer phylogenetic relationships. Compared to current phylogenetic analysis pipelines (i.e., multiple sequence alignment-based phylogenetic analysis), analysis from PhageGE showed similar phage phylogeny information in a shorter computing time (approximately 13 seconds versus 30 minutes for 15 phage genomes). Moreover, the result from phylogenetic analysis can be easily exported in various graphical formats (e.g., SVG, PDF and JPEG) and textual formats (e.g., Newick and Nexus) and can be interactively managed and viewed through our designed user interface. In addition, PhageGE introduces an enhanced phage lifestyle prediction function, using a machine-learning approach with updated databases for conserved protein domains. The overall approaches applied for both phylogenetic analysis and lifestyle prediction demonstrate that analyses results from PhageGE are comparable to previously published tools (**Figures 3** and **5**), showing its effectiveness in accurately analysing phage phylogeny and predicting phage lifestyle**.** Notably, PhageGE incorporates a function of annotation comparison to facilitate the efficient organisation of genome annotation files derived from different annotation pipelines. This feature allows users to efficiently compare genome annotation data obtained with different tools. Overall, all fours functions from PhageGE serve as a guide for the exploration of phage genomic features and will expedite the clinical translation of phage therapy.

Conclusion

In conclusion, PhageGE is the first biologist-friendly tool for the analysis of phage genomes, offering improved functions compared to existing tools without the need for considerable programming skills. Uniquely incorporating features like phylogenetic analysis, interactive tree visualisation, lifestyle prediction, and genome annotation comparison, we anticipate that PhageGE will become an instrumental bioinformatic web server for phage genomic analysis, guiding experimental validations and advancing the development of phage therapy.

Methods

Implementation

PhageGE 1.0 was developed in R and is hosted on Shinyapps. This application seamlessly integrates various R packages, including Rshiny, seqinr, Biostrings, ape, textmineR, tidyverse, ggtree, ploty, ggplot, reticulate, and pyhmmer [22, 32-38]. Furthermore, it incorporates several key functions, including *k*-mer-based phylogeny estimation, phylogenetic tree visualisation, lifestyle prediction, and annotation comparison. To use PhageGE, input files in the standard WGS fasta format are required, along with textual tables in standard formats (e.g., csv or xlsx) containing sequence details and annotation information. The workflow is illustrated in **Figure 1**.

Phage genomic analysis pipeline

The functionalities offered in the web interface of PhageGE utilise WGS fasta files for phylogenetic analysis and lifestyle prediction. Users can input tree files (e.g., Newick or Nexus) and textual files (i.e., csv or xlsx) for phylogenetic tree visualisation and

genome annotation comparisons. Using these standard formats as input files facilitates effective use and simplifies data export for users.

Phylogenetic analysis and phylogenetic tree visualisation

The phylogenetic analysis function enables fast and efficient analysis of phage phylogeny. It includes phylogeny reconstruction based on the input WGS data and visualisation of phylogenetic information. This function incorporates a *k*-mer-based alignment-free phylogenetic approach [39]. Alignment-free phylogenetic approaches offer a scalable alternative for inferring phylogenetic relationships and computing local alignment boundaries from WGS data [40, 41]. This approach is particularly robust for genome sequences that exhibit genetic recombinations and rearrangements. It has demonstrated the ability to accurately reconstruct biologically relevant phylogenies with thousands of microbial genomes [42-44]. The description of this function is briefly outlined below.

Consider a sequence consisting of four characters (A, T, C, G) of length *k* (‘*k*-mer’), described by **Equation 1**. There are 4^k possible *k*-mers (**Equation 2**), which can serve as features of each genome. The value assigned to a specific *k*-mer feature will correspond to the number of occurrences of that *k*-mer in the genome. Using these *k*-mer features, a data matrix is generated with dimensions of the numbers of genomes of interest (*n* columns) by 4^k rows. To establish a representative probability distribution of the 4^k *k*-mers, each row of the data matrix is normslised by its row total. This normalisation results in a feature-frequency profile (F_k, described by **Equation 3**) for each *k*-mers sequence [39]. The Jensen-Shannon divergence (D_k, described by **Equation 4**) is then employed to estimate the genome pairwise distances [45]. Subsequently, the resulting distance matrix is used as an input for a clustering

algorithm (e.g., neighbor-joining algorithm) to summarise the relatedness of the phage genomes and construct a phylogenetic tree [35].

Equation 1: $C_k = < C_{k,1}, C_{k,2} \cdots C_{k,m} >$

Equation 2: $m = 4^k$

Equation 3: $F_{n_i,k} = \frac{C_{n_i,km}}{\sum_{n_i} C_{n_i,k}}$

Equation 4: $D_k = JS(F_{n_1,k}, F_{n_i,k})$

An interactive visualisation of a phylogenetic tree was generated either from the phylogenetic analysis function or a customised phylogenetic tree that includes additional information, such as species classification, duplication events, and bootstrap values. It is implemented using ggtree and ploty R packages [22], ensuring the ability to handle most common tree formats (e.g., Newick, Nexus, and tre).

Lifestyle prediction

The Lifestyle Prediction function in PhageGE generates a phage lifestyle probability table based on the input of phage WGS data. This function adapted previously reported approaches into our user-friendly interface [18, 23, 25]. By employing an improved search function (i.e. searching a sequence file against the build-in Hidden Markov Model [HMM] database), PhageGE provides an efficient way to predict phage lifestyle based on the phage genomic information.

In brief, we first conducted a search in the Conserved Domain Database (accessed: 11/2023) to collect protein domains from temperate phages [46]. The following key

words were used to identify relevant protein domains: ‘temperate’, ‘lysogen’, ‘integrase’, ‘excisionase’, ‘recombinase’, ‘transposase’, ‘parA|parB’, and ‘xerC|xerD’. We obtained a total of 477 protein domains from the initial collection, which were then subjected to a careful manual curation and filtration (e.g., minimal domain length >30 and validated in the existing experimental data), resulting in a refined set of 261 protein domains. Next, a lifestyle classification model was trained and tested using a published dataset consisting of 1,057 phages from 6 different families (*Inoviridae*, *Myoviridae*, *Plasmaviridae*, *Podoviridae*, *Siphoviridae*, and *Tectiviridae*) across 55 host genera, with known genome and lifestyle information [25]. The dataset was randomly split into training and testing sets, with a ratio of 60:40 (634 phages in the training set and 423 phages in the testing set). At this stage, the testing set was fully set aside for subsequent descriptions related to model training and development. For each genome sequence in the training set, we generated a list of all possible 6-frame translation sequences that were at least 40 amino acids long. HMMER3 was then used to search for the presence or absence of the various protein domains listed above, resulting in a vector for each phage describing the presence (1) or absence (0) of each domain [47]. This information allowed us to filter the initial set of 477 putatively useful protein domains down to the final set of 261. Subsequently, a Random Forest classifier was fitted to the training set of phage genomes, and cross-validation was employed to fine-tune the model hyper-parameters. The ‘best’ performing model was then selected by choosing the hyper-parameters that yielded the highest minimum accuracy across the independent validation set tests. The parameters of that model were then re-fitted to the entire training set data, resulting in the final model.

Annotation comparison

The Rapid Annotation using Subsystem Technology (RAST) server was developed in 2008 to annotate microbial genomes based on the manually curated SEED database [48]. The PHAge Search Tool – Enhanced Release (PHASTER) was specifically designed to identify and annotate prophage sequences within bacteria using prophage/virus databases [49]. More recently, another phage annotation tool, Pharokka, has been developed using PHROGS, CARD, and VFDB databases [50]. Since these pipelines employ different databases for phage genome annotation, it is possible to obtain different annotations from each pipeline. To provide more comprehensive annotation results, there is an urgent need for annotation comparison tables that incorporate all annotation information from RAST, PHASTER, and Pharokka. The Annotation Comparison function in PhageGE generates interactive tables that display comments and differing genome annotation information obtained from RAST, PHASTER, and Pharokka. This comparison includes checking the coding regions and related annotations from each pipeline. Moreover, it provides an overview of common and different annotation counts, facilitating the tracking of differences between the three pipelines. This function is implemented using the flextable, tidyselect, data.table, and tidyverse packages [37].

Code availability and requirements

- Project name: PhageGE (Phage Genome Exploration)
- Project homepage: https://github.com/JinxinMonash/PhageGE
- Operating system(s): Linux, Windows and MacOS (**Table 1**)
- Programming language: R
- License: MIT license

Data availability

In general, all data used in this work were from openly accessible public repositories and released with other publications under open-source licenses. The data used were solely for research purposes, and we confirm that they were not used for any other noncommercial or commercial purpose. The datasets supporting the results of this article are available in the Github repository, [https://github.com/JinxinMonash/PhageGE]. The data used as examples can be found in the release branch called “Example data” or “Example data.zip” within our repository.The GitHub repository also contains up-to-date tutorials.

Competing interests

The authors declare that they have no competing interests.

Funding

This work was supported by the National Institute of Allergy and Infectious Diseases of the National Institutes of Health [grant number R21 AI156766 to J.L.]. The content is solely the responsibility of the authors and does not necessarily represent the official views of the National Institute of Allergy and Infectious Diseases or the National Institutes of Health.

Author’s contributions

J.Z. collected all the data and participated in developing the webserver and writing the manuscript. J.H., Y.W.L., Y.Z., M.A. and D.G. and J.N.S. contributed to the

development of the web server. P.J.B., S.N., J.Z.Y., T.L.Z. and T.V. took part in the discussion of the data. J.Z., F.S. and J.L. conceived the study, coordinated the work and contributed to writing the manuscript. All authors are involved in the discussion and finalisation of the manuscript.

Acknowledgements

J.Z. is a recipient of the 2022 Faculty of Medicine, Nursing and Health Sciences Bridging Fellowship, Monash University. J.L. is an Australian National Health Medical Research Council (NHMRC) Investigator Research Fellow and T.V. is an Australian Research Council (ARC) Industrial Fellow. Y.W.L. is currently an employee of Certara, Australia and Co-Director of the Malaya Translational and Clinical Pharmacometrics Group, University of Malaya, Malaysia.

References

1. Murray CJ, Ikuta KS, Sharara F, Swetschinski L, Aguilar GR, Gray A, et al. Global burden of bacterial antimicrobial resistance in 2019: a systematic analysis. The Lancet. 2022;399 10325:629-55.
2. Luepke KH, Suda KJ, Boucher H, Russo RL, Bonney MW, Hunt TD, et al. Past, present, and future of antibacterial economics: increasing bacterial resistance, limited antibiotic pipeline, and societal implications. Pharmacotherapy: The Journal of Human Pharmacology and Drug Therapy. 2017;37 1:71-84.
3. Bassetti M and Garau J. Current and future perspectives in the treatment of multidrug-resistant Gram-negative infections. Journal of Antimicrobial Chemotherapy. 2021;76 (Suppl 4):iv23-iv37.
4. Böttcher L, Gersbach H and Wernli D. Restoring the antibiotic R&D market to combat the resistance crisis. Science and Public Policy. 2022;49 1:127-31.
5. Uyttebroek S, Chen B, Onsea J, Ruythooren F, Debaveye Y, Devolder D, et al. Safety and efficacy of phage therapy in difficult-to-treat infections: a systematic review. The Lancet Infectious Diseases. 2022; 22 8:E208-E220.
6. Kortright KE, Chan BK, Koff JL and Turner PE. Phage therapy: a renewed approach to combat antibiotic-resistant bacteria. Cell Host & Microbe. 2019;25 2:219-32.
7. Mousavi SM, Babakhani S, Moradi L, Karami S, Shahbandeh M, Mirshekar M, et al. Bacteriophage as a novel therapeutic weapon for killing colistin-resistant multi-drug-resistant and extensively drug-resistant gram-negative bacteria. Current Microbiology. 2021;78 12:4023-36.
8. Lin Y, Chang RY, Rao G, Jermain B, Han M-L, Zhao J, et al. Pharmacokinetics/pharmacodynamics of antipseudomonal bacteriophage

therapy in rats: a proof-of-concept study. Clinical Microbiology and Infection. 2020;26 9:1229-35.

9. Hyman P. Phages for phage therapy: isolation, characterization, and host range breadth. Pharmaceuticals. 2019;12 1:35.

10. Gordillo Altamirano FL and Barr JJ. Phage therapy in the postantibiotic era. Clinical Microbiology Reviews. 2019;32 2:e00066-18.

11. Roach DR, Leung CY, Henry M, Morello E, Singh D, Di Santo JP, et al. Synergy between the host immune system and bacteriophage is essential for successful phage therapy against an acute respiratory pathogen. Cell Host & Microbe. 2017;22 1:38-47. e4.

12. Harrison E and Brockhurst MA. Ecological and evolutionary benefits of temperate phage: what does or doesn't kill you makes you stronger. BioEssays. 2017;39 12:1700112.

13. Gill JJ and Hyman P. Phage choice, isolation, and preparation for phage therapy. Current Pharmaceutical Biotechnology. 2010;11 1:2-14.

14. Abedon ST, García P, Mullany P and Aminov R. Phage therapy: past, present and future. Frontiers Media SA, 2017, p. 981.

15. Debnath M, Prasad GB and Bisen PS. Omics technology. Molecular Diagnostics: Promises and Possibilities. Springer; 2010. p. 11-31.

16. Parmar KM, Dafale NA, Tikariha H and Purohit HJ. Genomic characterization of key bacteriophages to formulate the potential biocontrol agent to combat enteric pathogenic bacteria. Archives of Microbiology. 2018;200 4:611-22.

17. Philipson CW, Voegtly LJ, Lueder MR, Long KA, Rice GK, Frey KG, et al. Characterizing phage genomes for therapeutic applications. Viruses. 2018;10 4:188.

18. McNair K, Bailey BA and Edwards RA. PHACTS, a computational approach to classifying the lifestyle of phages. Bioinformatics. 2012;28 5:614-8.

19. Katoh K and Standley DM. MAFFT multiple sequence alignment software version 7: improvements in performance and usability. Molecular Biology and Evolution. 2013;30 4:772-80.

20. Stamatakis A. RAxML version 8: a tool for phylogenetic analysis and post-analysis of large phylogenies. Bioinformatics. 2014;30 9:1312-3.

21. Minh BQ, Schmidt HA, Chernomor O, Schrempf D, Woodhams MD, Von Haeseler A, et al. IQ-TREE 2: new models and efficient methods for phylogenetic inference in the genomic era. Molecular Biology and Evolution. 2020;37 5:1530-4.

22. Xu S, Li L, Luo X, Chen M, Tang W, Zhan L, et al. Ggtree: A serialized data object for visualization of a phylogenetic tree and annotation data. iMeta. 2022;3 1:e56.

23. Hockenberry AJ and Wilke CO. BACPHLIP: predicting bacteriophage lifestyle from conserved protein domains. PeerJ. 2021;9:e11396.

24. Le T, Nang SC, Zhao J, Yu HH, Li J, Gill JJ, et al. Therapeutic potential of intravenous phage as standalone therapy for recurrent drug-resistant urinary tract infections. Antimicrobial Agents and Chemotherapy. 2023;67 4:e00037-23.

25. Mavrich TN and Hatfull GF. Bacteriophage evolution differs by host, lifestyle and genome. Nature Microbiology. 2017;2 9:1-9.

26. Zhao Y, Feng L, Zhou B, Zhang X, Yao Z, Wang L, et al. A newly isolated bacteriophage vB8388 and its synergistic effect with aminoglycosides against

multi-drug resistant *Klebsiella oxytoca* strain FK-8388. Microbial Pathogenesis. 2023;174:105906.

27. Khan A, Rao TS and Joshi HM. Phage therapy in the Covid-19 era: Advantages over antibiotics. Current Research in Microbial Sciences. 2022;3:100115.

28. Camarillo-Guerrero LF, Almeida A, Rangel-Pineros G, Finn RD and Lawley TD. Massive expansion of human gut bacteriophage diversity. Cell. 2021;184 4:1098-109.

29. Carrigy NB, Larsen SE, Reese V, Pecor T, Harrison M, Kuehl PJ, et al. Prophylaxis of *Mycobacterium tuberculosis* H37Rv infection in a preclinical mouse model via inhalation of nebulized bacteriophage D29. Antimicrobial Agents and Chemotherapy. 2019;63 12:e00871-19.

30. Martinez-Vaz BM and Mickelson MM. In silico phage hunting: bioinformatics exercises to identify and explore bacteriophage genomes. Frontiers in Microbiology. 2020;11:577634.

31. Wu J, Liu Q, Li M, Xu J, Wang C, Zhang J, et al. PhaGAA: an integrated web server platform for phage genome annotation and analysis. Bioinformatics. 2023;39 3:btad120.

32. Sievert C. Interactive web-based data visualization with R, plotly, and shiny. Journal of the Royal Statistical Society Series A: Statistics in Society. 2020;184:1150.

33. Charif D and Lobry JR. SeqinR 1.0-2: a contributed package to the R project for statistical computing devoted to biological sequences retrieval and analysis. Structural approaches to sequence evolution. Springer; 2007. p. 207-32.

34. Pagès H, Aboyoun P, Gentleman R, DebRoy S. Biostrings: Efficient manipulation of biological strings. 2024. Biostrings (Version 2.70.2) https://bioconductor.org/packages/Biostrings.

35. Paradis E and Schliep K. ape 5.0: an environment for modern phylogenetics and evolutionary analyses in R. Bioinformatics. 2019;35 3:526-8.

36. Jones T, Doane W and Jones MT. Package ‘textmineR’. Functions for text mining and topic modeling. 2021. textmineR (Version 3.0.5) https://github.com/TommyJones/textmineR.

37. Wickham H and Wickham MH. Welcome to the tidyverse. Journal of Open Source Software. 2019. 4(43), 1686.

38. Wickham H and Wickham MH. ggplot2: Elegant Graphics for Data Analysis. Springer-Verlag New York . 2016. https://ggplot2.tidyverse.org/

39. Sims GE, Jun S-R, Wu GA and Kim S-H. Alignment-free genome comparison with feature frequency profiles (FFP) and optimal resolutions. Proceedings of the National Academy of Sciences. 2009;106 8:2677-82.

40. Mapleson D, Garcia Accinelli G, Kettleborough G, Wright J and Clavijo BJ. KAT: a K-mer analysis toolkit to quality control NGS datasets and genome assemblies. Bioinformatics. 2017;33 4:574-6.

41. Jain C, Koren S, Dilthey A, Phillippy AM and Aluru S. A fast adaptive algorithm for computing whole-genome homology maps. Bioinformatics. 2018;34 17:i748-i56.

42. Bernard G, Chan CX and Ragan MA. Alignment-free microbial phylogenomics under scenarios of sequence divergence, genome rearrangement and lateral genetic transfer. Scientific Reports. 2016;6 1:1-12.

43. Jacobus AP, Stephens TG, Youssef P, González-Pech R, Ciccotosto-Camp MM, Dougan KE, et al. Comparative genomics supports that Brazilian bioethanol Saccharomyces cerevisiae comprise a unified group of domesticated strains related to cachaça spirit yeasts. Frontiers in Microbiology. 2021;12:644089.
44. Bernard G, Greenfield P, Ragan MA and Chan CX. k-mer similarity, networks of microbial genomes, and taxonomic rank. Msystems. 2018;3 6:e00257-18.
45. Sims GE and Kim S-H. Whole-genome phylogeny of *Escherichia coli*/*Shigella* group by feature frequency profiles (FFPs). Proceedings of the National Academy of Sciences. 2011;108 20:8329-34.
46. Lu S, Wang J, Chitsaz F, Derbyshire MK, Geer RC, Gonzales NR, et al. CDD/SPARCLE: the conserved domain database in 2020. Nucleic Acids Research. 2020;48 D1:D265-D8.
47. Eddy SR. Profile hidden Markov models. Bioinformatics. 1998;14 9:755-63.
48. Overbeek R, Olson R, Pusch GD, Olsen GJ, Davis JJ, Disz T, et al. The SEED and the Rapid Annotation of microbial genomes using Subsystems Technology (RAST). Nucleic Acids Research. 2014;42 D1:D206-D14.
49. Arndt D, Grant JR, Marcu A, Sajed T, Pon A, Liang Y, et al. PHASTER: a better, faster version of the PHAST phage search tool. Nucleic Acids Research. 2016;44 W1:W16-W21.
50. Bouras G, Nepal R, Houtak G, Psaltis AJ, Wormald P-J and Vreugde S. Pharokka: a fast scalable bacteriophage annotation tool. Bioinformatics. 2023;39 1:btac776.

Table 1. Browsers and operating systems (OS) tested with PhageGE

OS	Chrome	Edge	Firefox	Safari
**Linux**	120.0	120.0	121.0	n/a
**MacOS**	107.0	108.0	107.0.1	15.6.1
**Windows**	105.0	108.0	107.0.1	n/a

n/a, not applicable

Table 2. Lifestyle prediction for 8 different phages

	Lytic	Temperate
**KP36**	0.993	0.007
**FK1979**	0.956	0.044
**vB8838**	0.969	0.031
**pKp20**	0.974	0.026
**NC_017985**	0	1
**NC_027339**	0.002	0.998
**NC_009815**	0.016	0.984
**NC_019768**	0.01	0.99

Figures legends:

Figure 1. The workflow and application of PhageGE.

Illustration of the workflow of PhageGE, highlighting its components and processes for phage genomic analysis. (1) **Phylogenetic analysis.** Input: Phage genome files in .fna or .fasta format are uploaded; Pre-processing: The uploaded genome files are processed to estimate parameters and the are hashed for further analysis. Distance Estimation: K-mers features are normolised and then used for Jaccard index computation. Distance estimation: Distances are estimated based on the computed Jaccard index. (2) **Visualisation**. The results are visualised using the ggtree package and sample information files in CSV format. (3) **Lifestyle Prediction**. Biosequence analysis (HMMER): Biosequence analysis is performed using HMMER. Prediction model: A prediction model based on a phage genome-lifestyle dataset is applied. Lifestyle prediction: The lifestyle of the phages is predicted with the uploaded phage genome. (4) **Annotation Comparison**. Data manipulation: Genome annotation files (phaster.txt, RAST.xls, Pharokka.gff) are manipulated with built-in functions. Annotation comparison table: An annotation comparison table is generated using built-in functions.

Figure 2. Overview of PhageGE and its related functions.

The main functions and item information in PhageGE are illustrated in the figure, highlighting the steps for phylogenetic analysis, tree visualisation, lifestyle prediction, and annotation comparison. **A.** Phylogenetic Analysis: Users can select the genomes of interest by uploading phage whole genome data files (.fasta or .fna), selecting the layout of the tree (i.e., phylogram, cladogram, fan, radial and tidy), and clicking the "Explore Tree" button to initiate the phylogenetic analysis. **B.** Phylogenetic Tree

Visualisation: Users can upload a tree file (Newick or .tre format) and related genome information file (.csv). The tree visualisation displays the phylogenetic relationships among the uploaded genomes, with detailed annotations. **C.** Lifestyle Prediction: Users can select a genome of interest for lifestyle prediction by uploading a fasta file (.fna or .fasta). By clicking the "Explore Lifestyle Prediction" button, the user can predict the lifestyle of the selected genome, displaying the results with relevant statistics. **D.** Annotation Comparison: Users can upload multiple annotation files (Phaster, RAST, and Pharokka) and select the type of comparison. The resulting comparison table displays the annotated features from each source, facilitating detailed comparative analysis.

Figure 3. Comparison of phylogeny estimations from PhageGE and MSA. **A**. Alignment-free phylogenetic trees of 15 phages inferred from WGS data, and **B.** the topology of the reference tree inferred from multiple sequence alignment of WGS. The trees illustrate the classification and related taxa positions, demonstrating the consistency and accuracy of PhageGE's alignment-free approach in relation to the traditional MSA-based method.

Figure 4. Interactive visualisation of the phylogenetic tree of 15 phages.

Each coloured dot represents one phage, with the colour indicating the associated taxa. The pink box illustrates the additional information that can be obtained by hovering the cursor over each dot.

Figure 5. Comparison of classification accuracy of PhageGE with previously published tools across all datasets analysed.

Incorrect classification involves misidentifying the phage lifestyle (temperate or lytic).

Manuscript_no_mark
Click here to access/download;Manuscript;GIGA-D-24-00040
PhageGE_main_withoutMark.docx

PhageGE: An interactive web platform for exploratory analysis and visualisation of bacteriophage genomes

Jinxin Zhao[1, 2*], Jiru Han[3], Yu-Wei Lin[1], Yan Zhu[1, 4], Michael Aichem[5], Dimitar Garkov[5], Phillip J. Bergen[1], Sue C. Nang[1], Jian-Zhong Ye[6, 7], Tieli Zhou[6, 7], Tony Velkov[8], Jiangning Song[2, 9], Falk Schreiber[5, 10], Jian Li[1, 2*]

[1] Infection Program and Department of Microbiology, Biomedicine Discovery Institute, Monash University, Clayton, VIC, Australia

[2] Monash Biomedicine Discovery Institute-Wenzhou Medical University Alliance in Clinical and Experimental Biomedicine, Monash University, Clayton, VIC, Australia

[3] Population Health and Immunity Division, The Walter and Eliza Hall Institute of Medical Research, Parkville, VIC, Australia

[4] Systems Biology Center, Tianjin Institute of Industrial Biotechnology, Chinese Academy of Sciences, Tianjin, China

[5] Department of Computer and Information Science, University of Konstanz, Konstanz, Germany

[6] Key Laboratory of Clinical Laboratory Diagnosis and Translational Research of Zhejiang Province, Department of Clinical Laboratory, The First Affiliated Hospital of Wenzhou Medical University, Zhejiang, China

[7] Wenzhou Medical University-Monash Biomedicine Discovery Institute Alliance in Clinical and Experimental Biomedicine, The First Affiliated Hospital of Wenzhou Medical University,Wenzhou, China

[8] Department of Pharmacology, Biomedicine Discovery Institute, Monash University, Melbourne, VIC, Australia

[9] Department of Biochemistry and Molecular Biology, Biomedicine Discovery Institute, Monash University, Melbourne, VIC, Australia

[10] Faculty of Information Technology, Monash University, Clayton, VIC, Australia

Email addresses: JZ, jinxin.zhao@monash.edu; JH, han.ji@wehi.edu.au; YL, yu-wei.lin@monash.edu; YZ, Yan.Zhu@monash.edu; MA, michael.aichem@uni-konstanz.de; DG, dimitar.garkov@uni-konstanz.de; PB, phillip.bergen@monash.edu; SN, sue.nang@monash.edu; JY, jzye89@163.com; TZ, wyztli@163.com; TV, tony.velkov@monash.edu; JS, jiangning.song@monash.edu; FS, falk.schreiber@uni-konstanz.de; JL, jian.li@monash.edu.

Running title: PhageGE for bacteriophage genomic analysis

*Corresponding authors:
Dr Jinxin Zhao, Tel: +61 3 99056288, Email: jinxin.zhao@monash.edu;
Professor Jian Li, Tel: +61 3 99039172 Fax: +61 0399056450; Email: jian.li@monash.edu.

Abstract

Background: Antimicrobial resistance is a serious threat to global health. Due to the stagnant antibiotic discovery pipeline, bacteriophages (phages) have been proposed as an alternative therapy for the treatment of infections caused by multidrug-resistant (MDR) pathogens. Genomic features play an important role in phage pharmacology. However, our knowledge of phage genomics is sparse and the use of existing bioinformatic pipelines and tools requires considerable bioinformatic expertise. These challenges have substantially limited the clinical translation of phage therapy. **Findings:** A user-friendly graphical interface application, PhageGE (Phage Genome Explorer), was developed for the interactive analysis of phage genomes. The new R Shiny webserver, PhageGE, was designed for analysing phage whole-genome sequence (WGS) data. PhageGE integrates several existing R packages and combines them with several newly developed functions to perform phylogeny analysis and lifestyle prediction. The webserver offers several additional key functions, including interactive phylogenetic tree visualisation and annotation comparison. The output from PhageGE can be exported directly with publication-quality images.

Conclusions: PhageGE is a valuable tool for analysing phage genome data and may expedite the development and clinical translation of phage therapy. PhageGE is publicly available at http://phagege.com/.

Keywords: Phage genome, biological web application, genomic analysis, phylogeny, lifestyle

Introduction

The rapid emergence and spread of antimicrobial resistance (AMR) is one of the three greatest threats to human health globally [1]. It is estimated that by 2050, life-threatening infections caused by antimicrobial-resistant pathogens will kill more people than any other diseases [2]. Of particular concern is the increased prevalence of infections caused by Gram-negative pathogens, which are more difficult to treat than Gram-positive pathogens [3]. Given the sluggish global antibiotic pipeline [4], bacteriophages (phages) have attracted significant attention over the last decade as a potential alternative therapy for bacterial infections [5]. Phages are bacterial viruses and the advantages of phage therapy over antibiotics include a narrow spectrum of activity, the capacity to multiply at the infection site, and safety [6-8]. Optimising phage therapy in patients requires key pharmacological information, including infection cycle, gene content, and phage taxonomy [9, 10]. For example, temperate phages do not immediately lyse bacterial host cells and have an inherent capacity to mediate the transfer of genes between bacteria, potentially facilitating increased bacterial virulence and AMR. In contrast, lytic phages kill bacteria upon infection and are commonly used for the treatment of MDR bacterial infections in patients [11-14].

Multi-omics has the potential to expedite the clinical translation of phage therapy for the treatment of MDR bacterial infections [15]. For example, whole genome-based phylogenetic analysis offers significant advantages in understanding phage evolutionary dynamics and designing potential phage cocktails [16, 17]. Furthermore, combining whole-genome sequencing (WGS) with *in silico* prediction enables rapid prediction of phage lifestyle [18]. Several popular bioinformatic pipelines and tools are available for multiple sequence alignment (MAFFT) [19], phylogenetic reconstruction (RAxML and IQ-TREE) [20, 21], visualisation of phylogeny (ggtree) [22], and phage

lifestyle prediction (PHACTS and BACPHLIP) [18, 23]; however, utilising these tools requires proficient programming skills. Therefore, a biologist-friendly platform for phage genomic analyses is urgently needed to overcome the challenges associated with the requirement for advanced programming expertise.

Here, we developed an integrated webserver platform, PhageGE, that offers four key functionalities: phage phylogenetic analysis, tree visualisation, lifestyle prediction, and manipulation of phage genome annotation datasets. PhageGE differs from existing phage genomic analysis tools in that it facilitates the seamless export of all associated results in a publication-ready format without requiring complex procedures or long running times. Overall, PhageGE provides a biologist-friendly interface to streamline phage genomic analysis with WGS data.

Results

The PhageGE webserver (biotoolsID: biotools:phagege and RRID: SCR_025380) was designed to ensure biologist-friendliness and compatibility with major web browsers, including Google Chrome, Mozilla Firefox, Apple Safari, and Microsoft Edge (**Table 1**).

Webserver submission and case studies

To demonstrate the functions and the scope of application of PhageGE, we herein describe the results of a case study using PhageGE, including phage whole genome data (i.e., .fasta), a phylogenetic tree file (i.e., .tre), and genome annotation data (i.e., .xls, .txt and .gff), which are collectively referred to as “Example Data” (**Figure 1**). The complete set of Example Data used in the case studies can be accessed on the PhageGE GitHub repository (https://github.com/JinxinMonash/PhageGE).

Phage phylogenetic analysis and visualisation

To illustrate the phylogenetic analysis function in PhageGE and its application in clinical translation, we analysed our GitHub example dataset, which consists of 15 phage genomes. The hosts of the 15 phage genomes in the phylogenetic analysis are from 3 different bacterial species: *Citrobacter freundii, Escherichia coli, and Klebsiella pneumoniae* (**Figure 2A**). This dataset includes one anti-Klebsiella phage, pKp20, which was isolated in our lab and used in a clinical case [24]. In that case, a recurrent urinary tract infection [rUTI] was successfully treated with 4 weeks of adjunctive intravenous bacteriophage therapy, with no recurrence during a year of follow-up [24]. Both taxonomy information from phylogeny analysis and the lifestyle prediction played key roles in the selection of pKp20 over a wide range of phages [24]. The phage WGS data in the .fna or .fasta format can be obtained either from NCBI or prepared locally using standard genome assembly pipelines (e.g., SPAdes) based on the previous BLASTn result [24]. To compare the results obtained from PhageGE with the multiple sequence alignment-based approach, we also conducted a multiple sequence alignment-based phylogenetic analysis using MAFFT v7.47 and fasttree v2.1.10, alongside the phylogenetic analysis using PhageGE. We firstly uploaded the selected fasta files or a multi-fasta file which contains all phage genomes on the Phylogenetic Analysis page in PhageGE, then selected the layout of the tree (i.e., phylogram, cladogram, fan, radial, or tidy) and clicked the “Explore Tree” icon. The resulting phylogenetic tree, representing the relationships among the uploaded genomes, was generated using the built-in *k*-mer-based alignment-free phylogenetic approach, as detailed in the Methods section (**Figures 2A and 3A**). To enhance the clarity, we manually highlighted the 15 phages with distinct colours according to their genus. Comparison of the phylogenetic trees generated by PhageGE and MAFFT revealed

that both trees shared largely the same classification (e.g., positions of each phage and the related taxa) (**Figure 3**). Moreover, PhageGE demonstrates a significant improvement in runtime efficiency. For example, on a 2-GHz CPU with 64 GB RAM server, the runtimes of generating phylogenetics trees by PhageGE were 0.22 minutes for 15 phage genomes and 4.42 minutes for 146 phage genomes. In contract, the MSA-based approach (using tools like MAFFT along with FastTree) took 30 minutes and 296 minutes, respectively. This demonstrates that the performance of the phylogenetic analysis of PhageGE is accurate, fast and comparable to the multiple sequence alignment-based approach.

The phylogenetic visualisation function handles the phylogenetic tree along with diverse accompanying data. Its aim is to provide an interactive visualisation platform that enhances the accessibility of phylogenetic data and facilitates the phylogenetic analysis of phage comparative genomics studies. The phylogenetic tree and associated data can be extracted using a built-in function within PhageGE. This function is illustrated using a tree file “phage.tre” obtained from phage phylogenetic analysis (whether generated by PhageGE or other phylogenetic analysis pipeline) and a sample information file named “sample_info.csv” containing the taxonomy information for all 14 phages (**Figure 2B**). As shown in **Figure 4**, each dot in the dendrogram represents one phage with the colour indicating its taxonomic classification in the same genus. In addition, detailed information of each phage (e.g., name and taxonomy) can be easily accessed by hovering the cursor over the dot of interest (as indicated by the pink box in **Figure 4**). This interactive feature allows users to dynamically integrate and visualise the underlying information in a user-friendly manner.

Performance of phage lifestyle prediction

The lifestyle prediction function builds on a Random Forest classifier that incorporates up-to-date conserved protein domains with the ability to classify temperate and lytic phages using WGS data. To evaluate its performance, we compared the function with other published tools using the dataset of 1,057 phages in the literature [25]. The PhageGE lifestyle prediction function achieved the lowest error rates (0%, 1.2%, 0.3% and 2.5%, equivalent to 100%, 98.8%, 99.7% and 97.5% classification accuracy, respectively) across all tested datasets, substantially outperforming those existing tools for phage lifestyle classification (**Figure 5**). The prediction accuracy of PhageGE exceeded that of the most accurate existing tool, BACPHLIP, which had prediction accuracies of 99.8%, 98.3%, 99.2% and 96.5%, respectively (**Figure 5**). Similarly, WGS data for individual phages (e.g., *Klebsiella* phage KP36.fasta, vB8388.fasta, and FK1979.fasta from the example dataset described here) can be uploaded as input to generate the phage lifestyle probability table (**Figure 2C** and **Table 2**). The result presented in **Table 2** predicts that *Klebsiella* phages KP36 (a model phage in our laboratory), FK1979, and vB8388 [26] (two phages isolated from hospital sewage, The First Affiliated Hospital of Wenzhou Medical University, China), and pKp20 (used in the rUTI clinical case study) [24], are highly likely lytic phages, with the probability of 99.3%, 95.6% and 96.9%, respectively. Meanwhile, the four phages from the NCBI in **Table 2** NC_017985, NC_027339, NC_009815, and NC_019768 are highly likely temperate phages. This function empowers users to rapidly analyse the lifestyle of a phage of interest *in silico* with high prediction accuracy, providing key insights into the intricate phage ecosystems and enabling optimal design of phage therapy.

Comparison of phage genome annotation

Notably, PhageGE also provides a function to compare phage genome annotations obtained from different pipelines (i.e., Pharokka, Phaster and RAST). This analysis

involves the integration of R package flextable, which allows for the generation of downloadable comparison results in multiple formats (e.g., csv, Excel and PDF). The user interface offers the flexibility to rank the results based on multiple parameters (e.g., location and/or length of the coding sequence [CDS]). In the case study presented here, we used PhageGE to compare genome annotations of *Klebsiella* phages KP36, vB8838, and FK1979 generated from Phaster, RAST, and Pharokka (**Figure 2D**). By selecting “common_annotation”, a table with 75, 45, and 51 genes that were annotated in all three pipelines was generated for KP36, vB8838, and FK1979, respectively. We also identified 17, 7, and 12 unique genes from the Pharokka pipeline by selecting the “Pharokka_only” option. To gain a better understanding of those unique annotated genes, PhageGE allows users to directly copy and download both the nucleotide and amino acid sequences associated with the genes from the interactive table. This feature facilitates further investigation of these unique annotations.

Discussion

With the dramatic rise in MDR bacterial infections, phage therapy has emerged as a safe and potentially effective alternative treatment option to antibiotics [27]. However, the development of effective phage therapies is complex, involving the isolation, culturing, characterisation, and timely preparation of efficacious phages. Traditionally, this process is time-consuming and costly [28, 29]. Nevertheless, with the next-generation sequencing techniques, it has become possible to rapidly and cost-effectively characterise phages. Despite this advancement, there is a paucity of intuitive tools available for phage genomics, with the majority requiring operation in

command-line mode. The availability of large phage genomic datasets presents unique opportunities to develop bioinformatics tools that aid in phage biology and pharmacology research. The use of computational methods to study phages has shown promise in generating novel insights, such as phylogeny and lifestyle, through bioinformatic analysis [18, 25, 30]. However, there is currently no single tool available that encompasses all those functions (e.g., phylogenetic analysis, tree visualisation, lifestyle prediction, and genome annotation comparison) in the webserver platform. Herein, we describe the development of the PhageGE webserver GUI streamlined for user-friendly phage genomic analysis.

PhageGE is a novel, biologist-friendly GUI application for the interactive analysis of phage genomes. The overarching goal of PhageGE is to provide an interactive analysis and visualisation platform for the rapid exploration of phage genomic associations, thereby promoting efficient genomic data-driven discovery of phage therapy. PhageGE comprises a set of functions for phage genomic analysis, including phylogenetic analysis, tree visualisation, lifestyle prediction, and genome annotation comparison. While current tools like PhaGAA can provide lifestyle reorganisation analysis, their primary utility lies in analysing phage lifestyle for their prefered phage dataset (e.g., gut flora of human neonates) [31]. In contrast, PhageGE integrates a more comprehenstive dataset with a wide range of phage genomes, allowing for broader and deeper exploration of phage lifestyles. Moreover, the comparsion of annotations from different pipelines highlights the key role of PhageGE in advancing phage genomics through enhanced analysis and visualisation functions. To exemplify the utility of PhageGE, we investigated the phylogeny, lifestyle, and annotation comparison of *Klebsiella* phages KP36, vB8838, and FK1979, which were independently isolated in two different countries. Our findings demonstrate that the

various functions of PhageGE yield comparable or better results than existing state-of-the-art approaches. These results highlight the significant potential of PhageGE in analysing various phage genomic features using phage WGS data.

Notably, PhageGE requires only phage WGS data as the input for conducting the related analysis. The phage phylogenetic analysis function takes phage WGS in the fasta format as input and applies an alignment-free phylogenetic approach to infer phylogenetic relationships. Compared to current phylogenetic analysis pipelines (i.e., multiple sequence alignment-based phylogenetic analysis), analysis from PhageGE showed similar phage phylogeny information in a shorter computing time (approximately 13 seconds versus 30 minutes for 15 phage genomes). Moreover, the result from phylogenetic analysis can be easily exported in various graphical formats (e.g., SVG, PDF and JPEG) and textual formats (e.g., Newick and Nexus) and can be interactively managed and viewed through our designed user interface. In addition, PhageGE introduces an enhanced phage lifestyle prediction function, using a machine-learning approach with updated databases for conserved protein domains. The overall approaches applied for both phylogenetic analysis and lifestyle prediction demonstrate that analyses results from PhageGE are comparable to previously published tools (**Figures 3** and **5**), showing its effectiveness in accurately analysing phage phylogeny and predicting phage lifestyle**.** Notably, PhageGE incorporates a function of annotation comparison to facilitate the efficient organisation of genome annotation files derived from different annotation pipelines. This feature allows users to efficiently compare genome annotation data obtained with different tools. Overall, all fours functions from PhageGE serve as a guide for the exploration of phage genomic features and will expedite the clinical translation of phage therapy.

Conclusion

In conclusion, PhageGE is the first biologist-friendly tool for the analysis of phage genomes, offering improved functions compared to existing tools without the need for considerable programming skills. Uniquely incorporating features like phylogenetic analysis, interactive tree visualisation, lifestyle prediction, and genome annotation comparison, we anticipate that PhageGE will become an instrumental bioinformatic web server for phage genomic analysis, guiding experimental validations and advancing the development of phage therapy.

Methods

Implementation

PhageGE 1.0 was developed in R and is hosted on Shinyapps. This application seamlessly integrates various R packages, including Rshiny, seqinr, Biostrings, ape, textmineR, tidyverse, ggtree, ploty, ggplot, reticulate, and pyhmmer [22, 32-38]. Furthermore, it incorporates several key functions, including *k*-mer-based phylogeny estimation, phylogenetic tree visualisation, lifestyle prediction, and annotation comparison. To use PhageGE, input files in the standard WGS fasta format are required, along with textual tables in standard formats (e.g., csv or xlsx) containing sequence details and annotation information. The workflow is illustrated in **Figure 1**.

Phage genomic analysis pipeline

The functionalities offered in the web interface of PhageGE utilise WGS fasta files for phylogenetic analysis and lifestyle prediction. Users can input tree files (e.g., Newick or Nexus) and textual files (i.e., csv or xlsx) for phylogenetic tree visualisation and

genome annotation comparisons. Using these standard formats as input files facilitates effective use and simplifies data export for users.

Phylogenetic analysis and phylogenetic tree visualisation

The phylogenetic analysis function enables fast and efficient analysis of phage phylogeny. It includes phylogeny reconstruction based on the input WGS data and visualisation of phylogenetic information. This function incorporates a *k*-mer-based alignment-free phylogenetic approach [39]. Alignment-free phylogenetic approaches offer a scalable alternative for inferring phylogenetic relationships and computing local alignment boundaries from WGS data [40, 41]. This approach is particularly robust for genome sequences that exhibit genetic recombinations and rearrangements. It has demonstrated the ability to accurately reconstruct biologically relevant phylogenies with thousands of microbial genomes [42-44]. The description of this function is briefly outlined below.

Consider a sequence consisting of four characters (A, T, C, G) of length *k* (‘*k*-mer’), described by **Equation 1**. There are 4^k possible *k*-mers (**Equation 2**), which can serve as features of each genome. The value assigned to a specific *k*-mer feature will correspond to the number of occurrences of that *k*-mer in the genome. Using these *k*-mer features, a data matrix is generated with dimensions of the numbers of genomes of interest (*n* columns) by 4^k rows. To establish a representative probability distribution of the 4^k *k*-mers, each row of the data matrix is normslised by its row total. This normalisation results in a feature-frequency profile (F_k, described by **Equation 3**) for each *k*-mers sequence [39]. The Jensen-Shannon divergence (D_k, described by **Equation 4**) is then employed to estimate the genome pairwise distances [45]. Subsequently, the resulting distance matrix is used as an input for a clustering

algorithm (e.g., neighbor-joining algorithm) to summarise the relatedness of the phage genomes and construct a phylogenetic tree [35].

Equation 1: $C_k = < C_{k,1}, C_{k,2} \cdots C_{k,m} >$

Equation 2: $m = 4^k$

Equation 3: $F_{n_i,k} = \frac{c_{n_i,k_m}}{\sum_{n_i} c_{n_i,k}}$

Equation 4: $D_k = JS(F_{n_1,k}, F_{n_i,k})$

An interactive visualisation of a phylogenetic tree was generated either from the phylogenetic analysis function or a customised phylogenetic tree that includes additional information, such as species classification, duplication events, and bootstrap values. It is implemented using ggtree and ploty R packages [22], ensuring the ability to handle most common tree formats (e.g., Newick, Nexus, and tre).

Lifestyle prediction

The Lifestyle Prediction function in PhageGE generates a phage lifestyle probability table based on the input of phage WGS data. This function adapted previously reported approaches into our user-friendly interface [18, 23, 25]. By employing an improved search function (i.e. searching a sequence file against the build-in Hidden Markov Model [HMM] database), PhageGE provides an efficient way to predict phage lifestyle based on the phage genomic information.

In brief, we first conducted a search in the Conserved Domain Database (accessed: 11/2023) to collect protein domains from temperate phages [46]. The following key

words were used to identify relevant protein domains: ‘temperate’, ‘lysogen’, ‘integrase’, ‘excisionase’, ‘recombinase’, ‘transposase’, ‘parA|parB’, and ‘xerC|xerD’. We obtained a total of 477 protein domains from the initial collection, which were then subjected to a careful manual curation and filtration (e.g., minimal domain length >30 and validated in the existing experimental data), resulting in a refined set of 261 protein domains. Next, a lifestyle classification model was trained and tested using a published dataset consisting of 1,057 phages from 6 different families (*Inoviridae*, *Myoviridae*, *Plasmaviridae*, *Podoviridae*, *Siphoviridae*, and *Tectiviridae*) across 55 host genera, with known genome and lifestyle information [25]. The dataset was randomly split into training and testing sets, with a ratio of 60:40 (634 phages in the training set and 423 phages in the testing set). At this stage, the testing set was fully set aside for subsequent descriptions related to model training and development. For each genome sequence in the training set, we generated a list of all possible 6-frame translation sequences that were at least 40 amino acids long. HMMER3 was then used to search for the presence or absence of the various protein domains listed above, resulting in a vector for each phage describing the presence (1) or absence (0) of each domain [47]. This information allowed us to filter the initial set of 477 putatively useful protein domains down to the final set of 261. Subsequently, a Random Forest classifier was fitted to the training set of phage genomes, and cross-validation was employed to fine-tune the model hyper-parameters. The ‘best’ performing model was then selected by choosing the hyper-parameters that yielded the highest minimum accuracy across the independent validation set tests. The parameters of that model were then re-fitted to the entire training set data, resulting in the final model.

Annotation comparison

The Rapid Annotation using Subsystem Technology (RAST) server was developed in 2008 to annotate microbial genomes based on the manually curated SEED database [48]. The PHAge Search Tool – Enhanced Release (PHASTER) was specifically designed to identify and annotate prophage sequences within bacteria using prophage/virus databases [49]. More recently, another phage annotation tool, Pharokka, has been developed using PHROGS, CARD, and VFDB databases [50]. Since these pipelines employ different databases for phage genome annotation, it is possible to obtain different annotations from each pipeline. To provide more comprehensive annotation results, there is an urgent need for annotation comparison tables that incorporate all annotation information from RAST, PHASTER, and Pharokka. The Annotation Comparison function in PhageGE generates interactive tables that display comments and differing genome annotation information obtained from RAST, PHASTER, and Pharokka. This comparison includes checking the coding regions and related annotations from each pipeline. Moreover, it provides an overview of common and different annotation counts, facilitating the tracking of differences between the three pipelines. This function is implemented using the flextable, tidyselect, data.table, and tidyverse packages [37].

Code availability and requirements

- Project name: PhageGE (Phage Genome Exploration)
- Project homepage: https://github.com/JinxinMonash/PhageGE
- Operating system(s): Linux, Windows and MacOS (**Table 1**)
- Programming language: R
- License: MIT license

Data availability

In general, all data used in this work were from openly accessible public repositories and released with other publications under open-source licenses. The data used were solely for research purposes, and we confirm that they were not used for any other noncommercial or commercial purpose. The datasets supporting the results of this article are available in the Github repository, [https://github.com/JinxinMonash/PhageGE]. The data used as examples can be found in the release branch called “Example data” or “Example data.zip” within our repository.The GitHub repository also contains up-to-date tutorials.

Competing interests

The authors declare that they have no competing interests.

Funding

This work was supported by the National Institute of Allergy and Infectious Diseases of the National Institutes of Health [grant number R21 AI156766 to J.L.]. The content is solely the responsibility of the authors and does not necessarily represent the official views of the National Institute of Allergy and Infectious Diseases or the National Institutes of Health.

Author’s contributions

J.Z. collected all the data and participated in developing the webserver and writing the manuscript. J.H., Y.W.L., Y.Z., M.A. and D.G. and J.N.S. contributed to the

development of the web server. P.J.B., S.N., J.Z.Y., T.L.Z. and T.V. took part in the discussion of the data. J.Z., F.S. and J.L. conceived the study, coordinated the work and contributed to writing the manuscript. All authors are involved in the discussion and finalisation of the manuscript.

Acknowledgements

J.Z. is a recipient of the 2022 Faculty of Medicine, Nursing and Health Sciences Bridging Fellowship, Monash University. J.L. is an Australian National Health Medical Research Council (NHMRC) Investigator Research Fellow and T.V. is an Australian Research Council (ARC) Industrial Fellow. Y.W.L. is currently an employee of Certara, Australia and Co-Director of the Malaya Translational and Clinical Pharmacometrics Group, University of Malaya, Malaysia.

References

1. Murray CJ, Ikuta KS, Sharara F, Swetschinski L, Aguilar GR, Gray A, et al. Global burden of bacterial antimicrobial resistance in 2019: a systematic analysis. The Lancet. 2022;399 10325:629-55.
2. Luepke KH, Suda KJ, Boucher H, Russo RL, Bonney MW, Hunt TD, et al. Past, present, and future of antibacterial economics: increasing bacterial resistance, limited antibiotic pipeline, and societal implications. Pharmacotherapy: The Journal of Human Pharmacology and Drug Therapy. 2017;37 1:71-84.
3. Bassetti M and Garau J. Current and future perspectives in the treatment of multidrug-resistant Gram-negative infections. Journal of Antimicrobial Chemotherapy. 2021;76 (Suppl 4):iv23-iv37.
4. Böttcher L, Gersbach H and Wernli D. Restoring the antibiotic R&D market to combat the resistance crisis. Science and Public Policy. 2022;49 1:127-31.
5. Uyttebroek S, Chen B, Onsea J, Ruythooren F, Debaveye Y, Devolder D, et al. Safety and efficacy of phage therapy in difficult-to-treat infections: a systematic review. The Lancet Infectious Diseases. 2022; 22 8:E208-E220.
6. Kortright KE, Chan BK, Koff JL and Turner PE. Phage therapy: a renewed approach to combat antibiotic-resistant bacteria. Cell Host & Microbe. 2019;25 2:219-32.
7. Mousavi SM, Babakhani S, Moradi L, Karami S, Shahbandeh M, Mirshekar M, et al. Bacteriophage as a novel therapeutic weapon for killing colistin-resistant multi-drug-resistant and extensively drug-resistant gram-negative bacteria. Current Microbiology. 2021;78 12:4023-36.
8. Lin Y, Chang RY, Rao G, Jermain B, Han M-L, Zhao J, et al. Pharmacokinetics/pharmacodynamics of antipseudomonal bacteriophage

therapy in rats: a proof-of-concept study. Clinical Microbiology and Infection. 2020;26 9:1229-35.

9. Hyman P. Phages for phage therapy: isolation, characterization, and host range breadth. Pharmaceuticals. 2019;12 1:35.

10. Gordillo Altamirano FL and Barr JJ. Phage therapy in the postantibiotic era. Clinical Microbiology Reviews. 2019;32 2:e00066-18.

11. Roach DR, Leung CY, Henry M, Morello E, Singh D, Di Santo JP, et al. Synergy between the host immune system and bacteriophage is essential for successful phage therapy against an acute respiratory pathogen. Cell Host & Microbe. 2017;22 1:38-47. e4.

12. Harrison E and Brockhurst MA. Ecological and evolutionary benefits of temperate phage: what does or doesn't kill you makes you stronger. BioEssays. 2017;39 12:1700112.

13. Gill JJ and Hyman P. Phage choice, isolation, and preparation for phage therapy. Current Pharmaceutical Biotechnology. 2010;11 1:2-14.

14. Abedon ST, García P, Mullany P and Aminov R. Phage therapy: past, present and future. Frontiers Media SA, 2017, p. 981.

15. Debnath M, Prasad GB and Bisen PS. Omics technology. Molecular Diagnostics: Promises and Possibilities. Springer; 2010. p. 11-31.

16. Parmar KM, Dafale NA, Tikariha H and Purohit HJ. Genomic characterization of key bacteriophages to formulate the potential biocontrol agent to combat enteric pathogenic bacteria. Archives of Microbiology. 2018;200 4:611-22.

17. Philipson CW, Voegtly LJ, Lueder MR, Long KA, Rice GK, Frey KG, et al. Characterizing phage genomes for therapeutic applications. Viruses. 2018;10 4:188.

18. McNair K, Bailey BA and Edwards RA. PHACTS, a computational approach to classifying the lifestyle of phages. Bioinformatics. 2012;28 5:614-8.
19. Katoh K and Standley DM. MAFFT multiple sequence alignment software version 7: improvements in performance and usability. Molecular Biology and Evolution. 2013;30 4:772-80.
20. Stamatakis A. RAxML version 8: a tool for phylogenetic analysis and post-analysis of large phylogenies. Bioinformatics. 2014;30 9:1312-3.
21. Minh BQ, Schmidt HA, Chernomor O, Schrempf D, Woodhams MD, Von Haeseler A, et al. IQ-TREE 2: new models and efficient methods for phylogenetic inference in the genomic era. Molecular Biology and Evolution. 2020;37 5:1530-4.
22. Xu S, Li L, Luo X, Chen M, Tang W, Zhan L, et al. Ggtree: A serialized data object for visualization of a phylogenetic tree and annotation data. iMeta. 2022;3 1:e56.
23. Hockenberry AJ and Wilke CO. BACPHLIP: predicting bacteriophage lifestyle from conserved protein domains. PeerJ. 2021;9:e11396.
24. Le T, Nang SC, Zhao J, Yu HH, Li J, Gill JJ, et al. Therapeutic potential of intravenous phage as standalone therapy for recurrent drug-resistant urinary tract infections. Antimicrobial Agents and Chemotherapy. 2023;67 4:e00037-23.
25. Mavrich TN and Hatfull GF. Bacteriophage evolution differs by host, lifestyle and genome. Nature Microbiology. 2017;2 9:1-9.
26. Zhao Y, Feng L, Zhou B, Zhang X, Yao Z, Wang L, et al. A newly isolated bacteriophage vB8388 and its synergistic effect with aminoglycosides against

multi-drug resistant *Klebsiella oxytoca* strain FK-8388. Microbial Pathogenesis. 2023;174:105906.

27. Khan A, Rao TS and Joshi HM. Phage therapy in the Covid-19 era: Advantages over antibiotics. Current Research in Microbial Sciences. 2022;3:100115.

28. Camarillo-Guerrero LF, Almeida A, Rangel-Pineros G, Finn RD and Lawley TD. Massive expansion of human gut bacteriophage diversity. Cell. 2021;184 4:1098-109.

29. Carrigy NB, Larsen SE, Reese V, Pecor T, Harrison M, Kuehl PJ, et al. Prophylaxis of *Mycobacterium tuberculosis* H37Rv infection in a preclinical mouse model via inhalation of nebulized bacteriophage D29. Antimicrobial Agents and Chemotherapy. 2019;63 12:e00871-19.

30. Martinez-Vaz BM and Mickelson MM. In silico phage hunting: bioinformatics exercises to identify and explore bacteriophage genomes. Frontiers in Microbiology. 2020;11:577634.

31. Wu J, Liu Q, Li M, Xu J, Wang C, Zhang J, et al. PhaGAA: an integrated web server platform for phage genome annotation and analysis. Bioinformatics. 2023;39 3:btad120.

32. Sievert C. Interactive web-based data visualization with R, plotly, and shiny. Journal of the Royal Statistical Society Series A: Statistics in Society. 2020;184:1150.

33. Charif D and Lobry JR. SeqinR 1.0-2: a contributed package to the R project for statistical computing devoted to biological sequences retrieval and analysis. Structural approaches to sequence evolution. Springer; 2007. p. 207-32.

34. Pagès H, Aboyoun P, Gentleman R, DebRoy S. Biostrings: Efficient manipulation of biological strings. 2024. Biostrings (Version 2.70.2) https://bioconductor.org/packages/Biostrings.

35. Paradis E and Schliep K. ape 5.0: an environment for modern phylogenetics and evolutionary analyses in R. Bioinformatics. 2019;35 3:526-8.

36. Jones T, Doane W and Jones MT. Package 'textmineR'. Functions for text mining and topic modeling. 2021. textmineR (Version 3.0.5) https://github.com/TommyJones/textmineR.

37. Wickham H and Wickham MH. Welcome to the tidyverse. Journal of Open Source Software. 2019. 4(43), 1686.

38. Wickham H and Wickham MH. ggplot2: Elegant Graphics for Data Analysis. Springer-Verlag New York . 2016. https://ggplot2.tidyverse.org/

39. Sims GE, Jun S-R, Wu GA and Kim S-H. Alignment-free genome comparison with feature frequency profiles (FFP) and optimal resolutions. Proceedings of the National Academy of Sciences. 2009;106 8:2677-82.

40. Mapleson D, Garcia Accinelli G, Kettleborough G, Wright J and Clavijo BJ. KAT: a K-mer analysis toolkit to quality control NGS datasets and genome assemblies. Bioinformatics. 2017;33 4:574-6.

41. Jain C, Koren S, Dilthey A, Phillippy AM and Aluru S. A fast adaptive algorithm for computing whole-genome homology maps. Bioinformatics. 2018;34 17:i748-i56.

42. Bernard G, Chan CX and Ragan MA. Alignment-free microbial phylogenomics under scenarios of sequence divergence, genome rearrangement and lateral genetic transfer. Scientific Reports. 2016;6 1:1-12.

43. Jacobus AP, Stephens TG, Youssef P, González-Pech R, Ciccotosto-Camp MM, Dougan KE, et al. Comparative genomics supports that Brazilian bioethanol Saccharomyces cerevisiae comprise a unified group of domesticated strains related to cachaça spirit yeasts. Frontiers in Microbiology. 2021;12:644089.
44. Bernard G, Greenfield P, Ragan MA and Chan CX. k-mer similarity, networks of microbial genomes, and taxonomic rank. Msystems. 2018;3 6:e00257-18.
45. Sims GE and Kim S-H. Whole-genome phylogeny of *Escherichia coli*/*Shigella* group by feature frequency profiles (FFPs). Proceedings of the National Academy of Sciences. 2011;108 20:8329-34.
46. Lu S, Wang J, Chitsaz F, Derbyshire MK, Geer RC, Gonzales NR, et al. CDD/SPARCLE: the conserved domain database in 2020. Nucleic Acids Research. 2020;48 D1:D265-D8.
47. Eddy SR. Profile hidden Markov models. Bioinformatics. 1998;14 9:755-63.
48. Overbeek R, Olson R, Pusch GD, Olsen GJ, Davis JJ, Disz T, et al. The SEED and the Rapid Annotation of microbial genomes using Subsystems Technology (RAST). Nucleic Acids Research. 2014;42 D1:D206-D14.
49. Arndt D, Grant JR, Marcu A, Sajed T, Pon A, Liang Y, et al. PHASTER: a better, faster version of the PHAST phage search tool. Nucleic Acids Research. 2016;44 W1:W16-W21.
50. Bouras G, Nepal R, Houtak G, Psaltis AJ, Wormald P-J and Vreugde S. Pharokka: a fast scalable bacteriophage annotation tool. Bioinformatics. 2023;39 1:btac776.

Table 1. Browsers and operating systems (OS) tested with PhageGE

OS	Chrome	Edge	Firefox	Safari
**Linux**	120.0	120.0	121.0	n/a
**MacOS**	107.0	108.0	107.0.1	15.6.1
**Windows**	105.0	108.0	107.0.1	n/a

n/a, not applicable

Table 2. Lifestyle prediction for 8 different phages

	Lytic	Temperate
**KP36**	0.993	0.007
**FK1979**	0.956	0.044
**vB8838**	0.969	0.031
**pKp20**	0.974	0.026
**NC_017985**	0	1
**NC_027339**	0.002	0.998
**NC_009815**	0.016	0.984
**NC_019768**	0.01	0.99

Figures legends:

Figure 1. The workflow and application of PhageGE.

Illustration of the workflow of PhageGE, highlighting its components and processes for phage genomic analysis. (1) **Phylogenetic analysis.** Input: Phage genome files in .fna or .fasta format are uploaded; Pre-processing: The uploaded genome files are processed to estimate parameters and the are hashed for further analysis. Distance Estimation: K-mers features are normolised and then used for Jaccard index computation. Distance estimation: Distances are estimated based on the computed Jaccard index. (2) **Visualisation**. The results are visualised using the ggtree package and sample information files in CSV format. (3) **Lifestyle Prediction**. Biosequence analysis (HMMER): Biosequence analysis is performed using HMMER. Prediction model: A prediction model based on a phage genome-lifestyle dataset is applied. Lifestyle prediction: The lifestyle of the phages is predicted with the uploaded phage genome. (4) **Annotation Comparison**. Data manipulation: Genome annotation files (phaster.txt, RAST.xls, Pharokka.gff) are manipulated with built-in functions. Annotation comparison table: An annotation comparison table is generated using built-in functions.

Figure 2. Overview of PhageGE and its related functions.

The main functions and item information in PhageGE are illustrated in the figure, highlighting the steps for phylogenetic analysis, tree visualisation, lifestyle prediction, and annotation comparison. **A.** Phylogenetic Analysis: Users can select the genomes of interest by uploading phage whole genome data files (.fasta or .fna), selecting the layout of the tree (i.e., phylogram, cladogram, fan, radial and tidy), and clicking the "Explore Tree" button to initiate the phylogenetic analysis. **B.** Phylogenetic Tree

Visualisation: Users can upload a tree file (Newick or .tre format) and related genome information file (.csv). The tree visualisation displays the phylogenetic relationships among the uploaded genomes, with detailed annotations. **C.** Lifestyle Prediction: Users can select a genome of interest for lifestyle prediction by uploading a fasta file (.fna or .fasta). By clicking the "Explore Lifestyle Prediction" button, the user can predict the lifestyle of the selected genome, displaying the results with relevant statistics. **D.** Annotation Comparison: Users can upload multiple annotation files (Phaster, RAST, and Pharokka) and select the type of comparison. The resulting comparison table displays the annotated features from each source, facilitating detailed comparative analysis.

Figure 3. Comparison of phylogeny estimations from PhageGE and MSA. A. Alignment-free phylogenetic trees of 15 phages inferred from WGS data, and **B.** the topology of the reference tree inferred from multiple sequence alignment of WGS. The trees illustrate the classification and related taxa positions, demonstrating the consistency and accuracy of PhageGE's alignment-free approach in relation to the traditional MSA-based method.

Figure 4. Interactive visualisation of the phylogenetic tree of 15 phages.

Each coloured dot represents one phage, with the colour indicating the associated taxa. The pink box illustrates the additional information that can be obtained by hovering the cursor over each dot.

Figure 5. Comparison of classification accuracy of PhageGE with previously published tools across all datasets analysed.

Incorrect classification involves misidentifying the phage lifestyle (temperate or lytic).

Figure 1 Click here to access/download;Figure;Figure 1.jpg

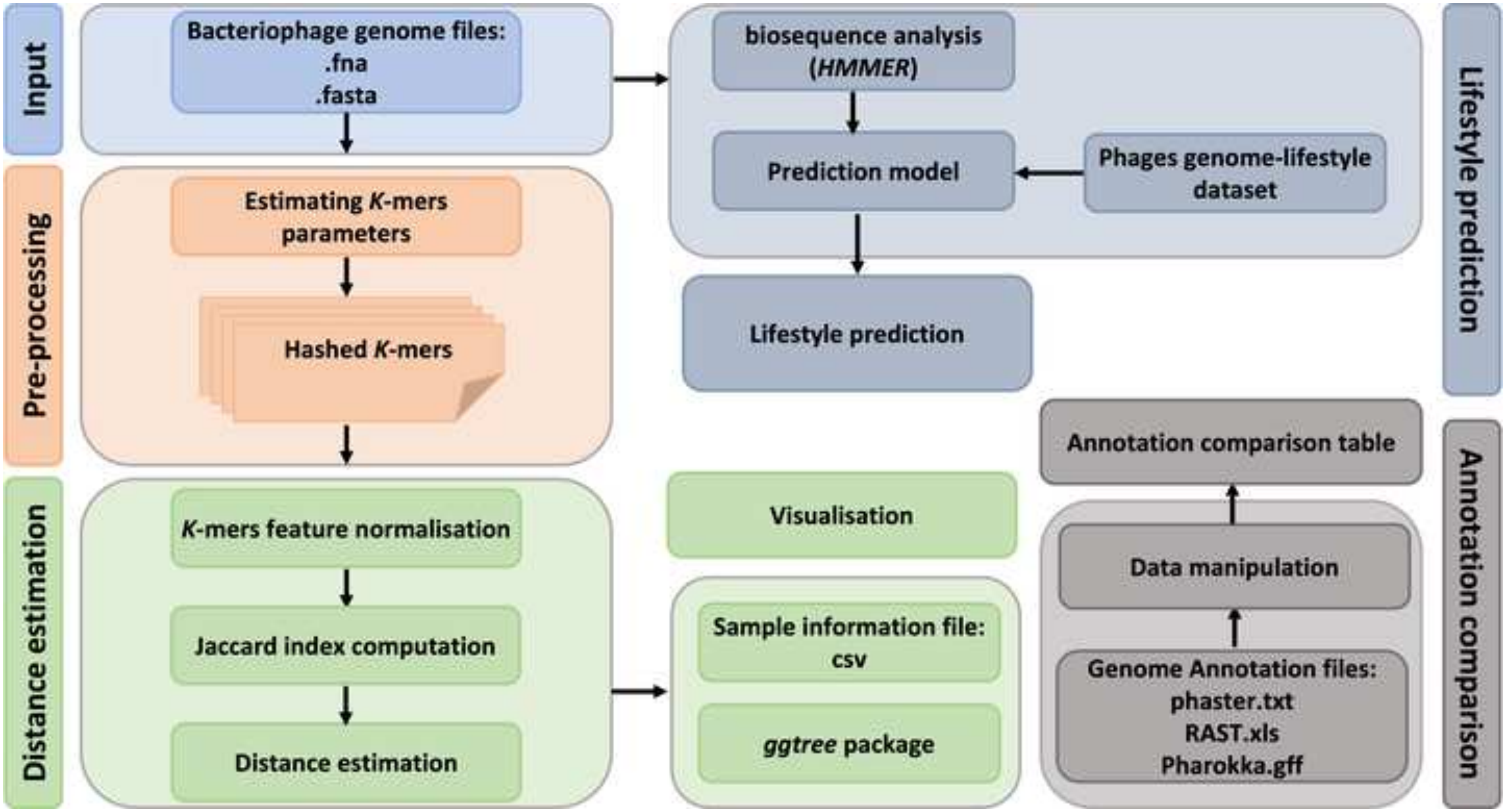

Figure 2

Click here to access/download;Figure;Figure 2.jpg

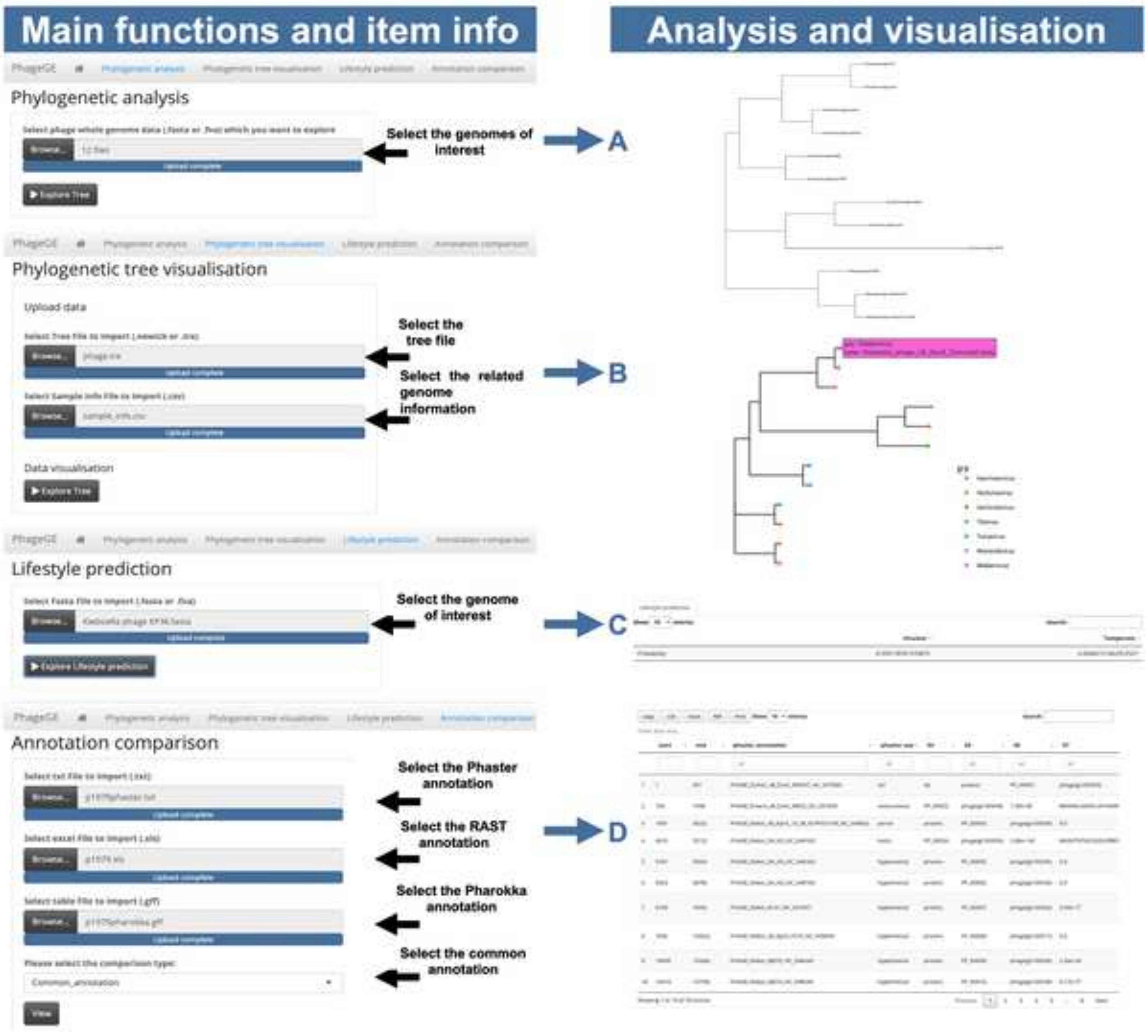

Figure 3 Click here to access/download;Figure;Figure 3.jpg

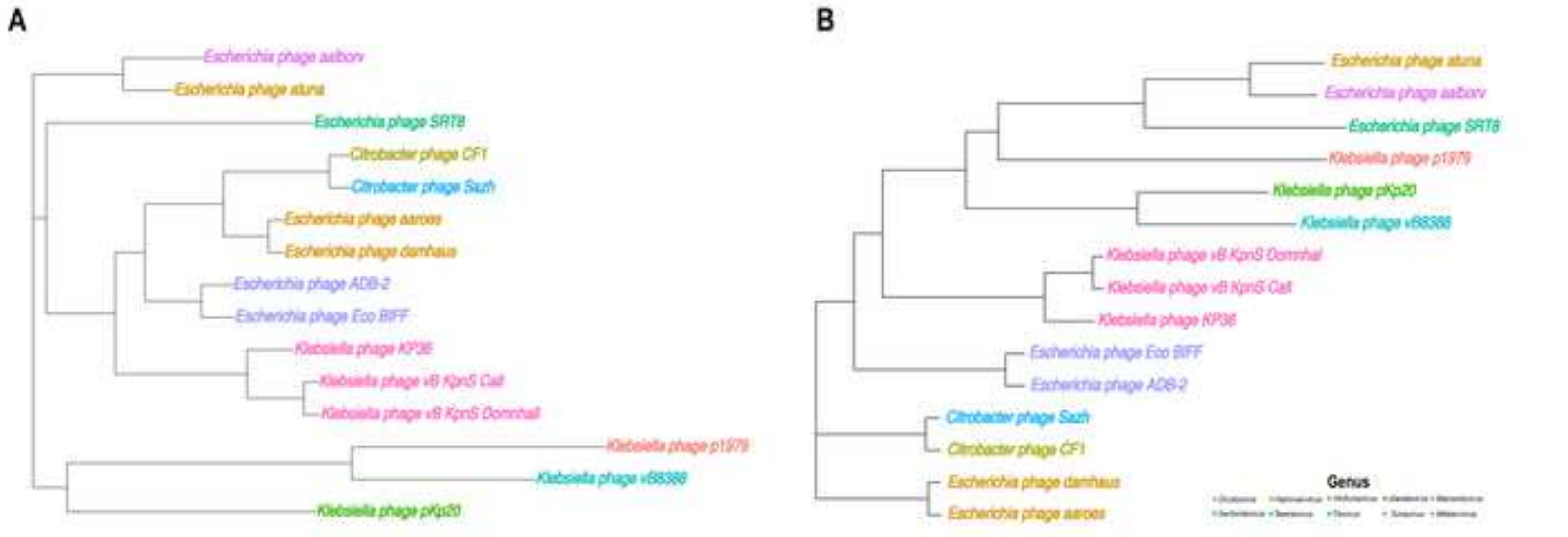

Figure 4

Click here to access/download;Figure;Figure 4.png

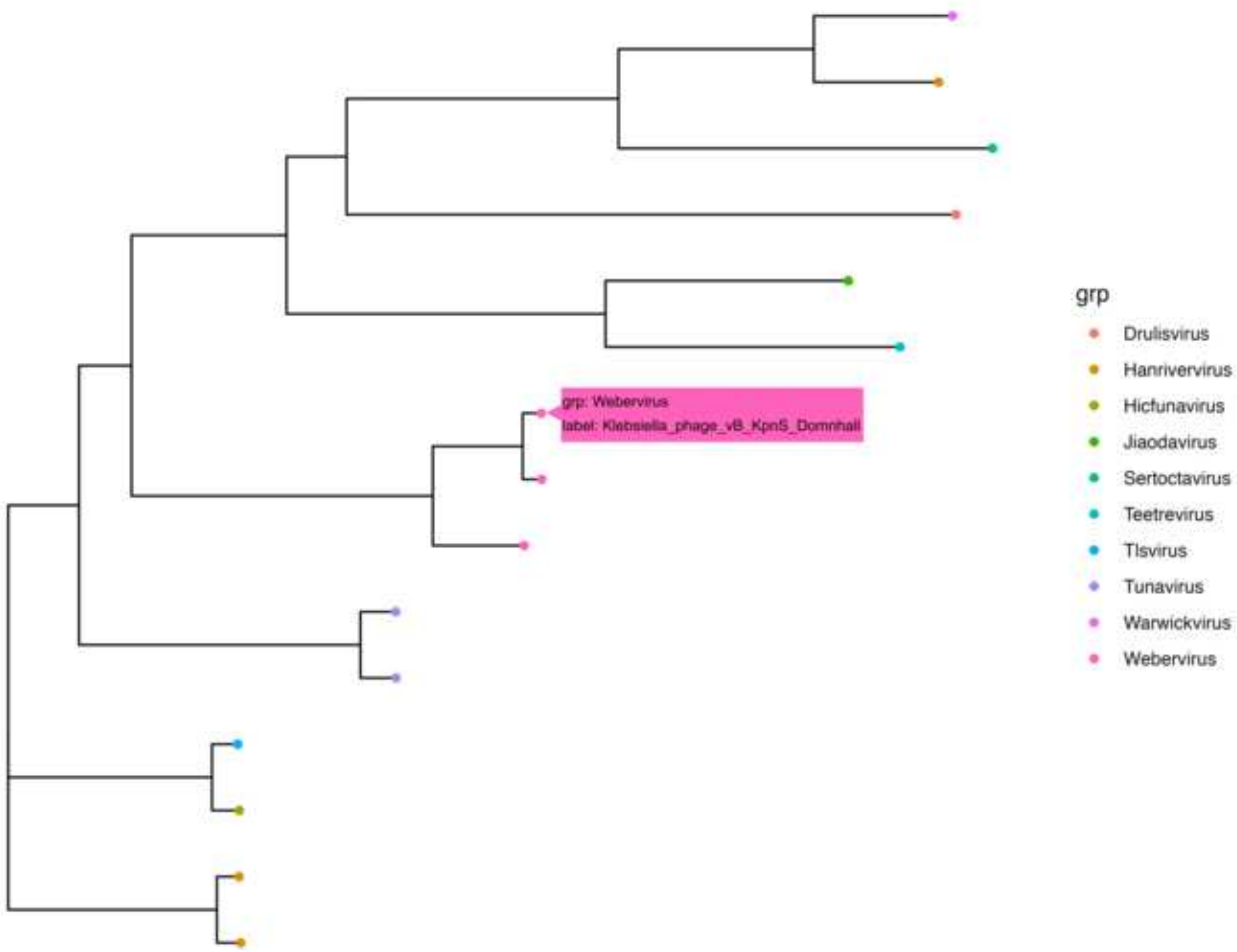

Figure 5

Click here to access/download;Figure;Figure 5.jpeg

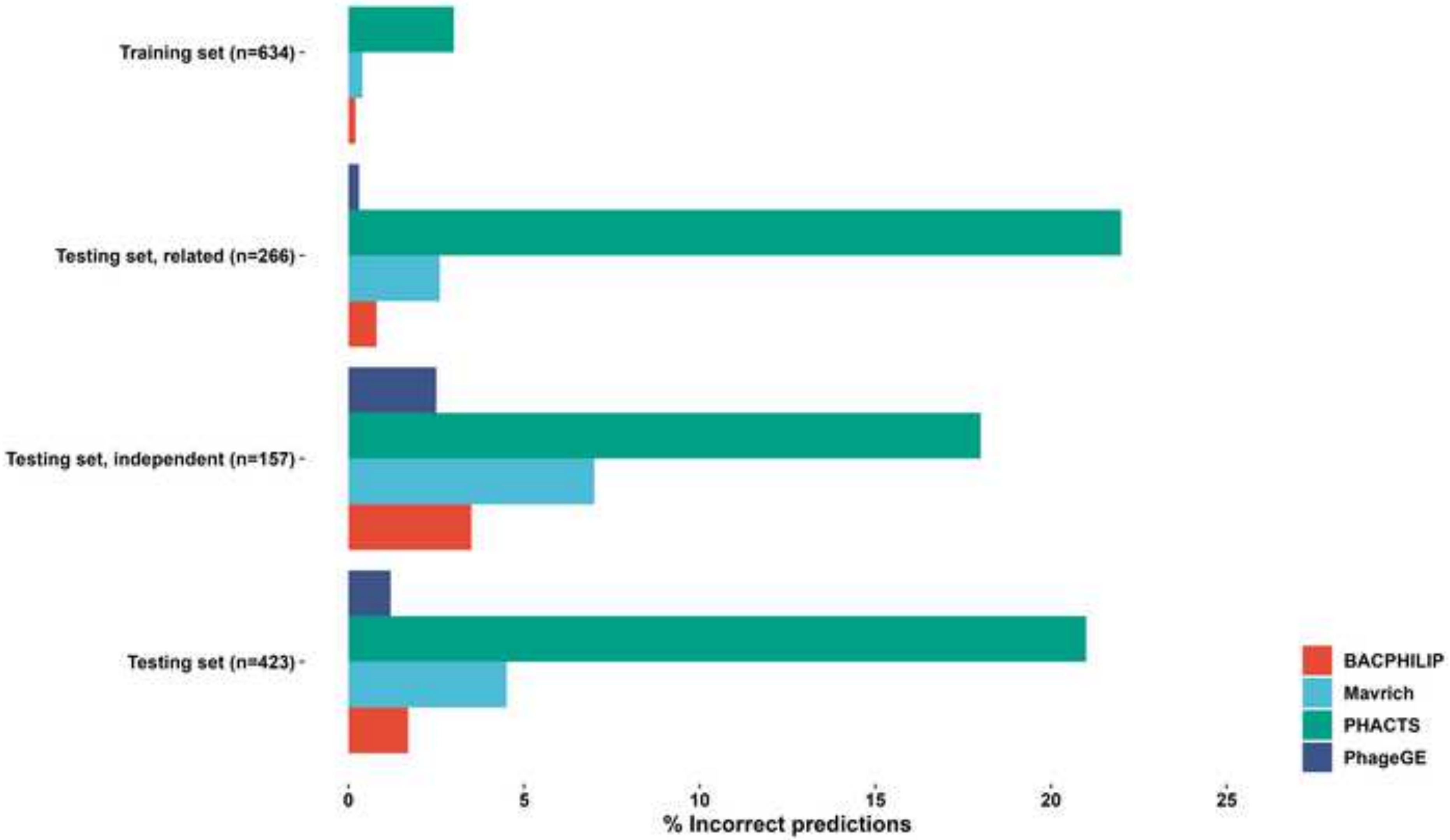

Cover letter and responses to reviewer
Click here to access/download;Personal Cover;GIGA-D-24-00040 Response to reviewer.docx

MONASH University

Professor Jian Li
Fellow of the American Academy of Microbiology
Head, Laboratory of Antimicrobial Systems Pharmacology
Monash Biomedicine Discovery Institute

Dr Scott Edmunds

Editor-in-Chief

GigaScience

June 29th, 2024

Re: Manuscript GIGA-D-24-00040

Dear Dr Edmunds,

Thank you for providing the reviewers' comments on our manuscript "*PhageGE: An interactive web platform for exploratory analysis and visualisation of bacteriophage genomes*" and for the opportunity to revise it. Please find below our point-by-point responses to the editor and reviewers' comments. For your convenience, all major changes have been highlighted in yellow. Line numbers mentioned in our responses below refer to the marked-up version of the manuscript.

Thank you and we are looking forward to your final decision.

Yours sincerely,

Jian Li PhD

19 Innovation Walk
Monash University
VIC 3800, Australia
Telephone: (+61 3) 990 39702 Facsimile: (+61 3) 9905 6450 Email: Jian.Li@monash.edu
Web: https://research.monash.edu/en/persons/jian-li

Unintended recipient: please notify as soon as possible and destroy all pages received

Professor Jian Li
Fellow of the American Academy of Microbiology
Head, Laboratory of Antimicrobial Systems Pharmacology
Monash Biomedicine Discovery Institute

Point-by-point responses

Editor comments:

1. Please register any new software application in the bio.tools and SciCrunch.org databases to receive RRID (Research Resource Identification Initiative ID) and biotoolsID identifiers, and include these in your manuscript. Computational workflows should be registered in workflowhub.eu and the DOIs cited in the relevant places in the manuscript. These will facilitate tracking, reproducibility and re-use of your tool.

Response: We have registered our application in bio.tools and SciCrunch.org databases and included the biotoolsID (biotools:phagege) and RRID (SCR_025380) in the revised manuscript (line 101).

Reviewers' comments:

Reviewer1:

The authors report here a new web-based tool called Phage Genome Explorer (PhageGE) for the interactive analysis of phage genomic data, which facilitates phylogenetic analysis and visualisation, the prediction of lytic vs., lysogenic lifestyles, and the interrogation of data generated by genome annotation tools (e.g., Pharokka). I commend the authors for developing this user-friendly tool that allows for greater access to non-experts. I believe this tool will have utility across clinical research and basic phage biology. I've tested the tool using both author supplied test data and data I've generated, and I have no major comments about the results and usability of PhageGE. However, I believe additional revisions are needed to strengthen the overall manuscript.

1. I would like to see the option to upload multi-fasta files implemented as a means to streamline usability. I think this can be implemented for both "phylogenetic analysis" and "lifestyle prediction" sections.

Response: We thank the reviewer for the suggestion and especially for providing the code for implementing multi-fasta format in our tools. We have incorporated the multi-fasta format into the "Phylogenetic analysis" function and revised the related description in the manuscript (lines 128-130). We have updated the previous "Lifestyle prediction" function for predicting multiple phage genomes simultaneously.

19 Innovation Walk
Monash University
VIC 3800, Australia
Telephone: (+61 3) 990 39702 Facsimile: (+61 3) 9905 6450 Email: Jian.Li@monash.edu
Web: https://research.monash.edu/en/persons/jian-li

Unintended recipient: please notify as soon as possible and destroy all pages received

Professor Jian Li
Fellow of the American Academy of Microbiology
Head, Laboratory of Antimicrobial Systems Pharmacology
Monash Biomedicine Discovery Institute

2. How does PhageGE scale to large metagenomic datasets? Unfortunately, I was unable to test this without the multi-fasta input option. However, I think it could scale nicely, especially with a circular tree format.

Response: We thank the reviewer for the suggestion. We have updated phageGE with a multi-fasta format input option and also provided an option for the final tree format (e.g., rectangular and circular format) (lines 128-132). We would like to clarify that the primary aim of PhageGE is to analyse phage genomic data, assuming that users already have assembled phage genomes rather than detecting them directly from large metagenomic datasets. This focus allows us to provide a robust and efficient tool specifically tailored for phage genome analysis. We apologise for any confusion this may have caused. The detection of phage sequences directly from large metagenomic datasets is beyond the current scope of PhageGE. Nevertheless, we acknowledge its importance and will consider developing this functionality in the next version of PhageGE.

3. Viral clusters have been shown to be important in determining viral diversity, and I think it would be a useful addition to the phylogenetic-based analyses. c.f., Camarillo-Guerrero et al., 2021. PMID: 33606979 and rBlast https://github.com/mhahsler/rBLAST

Response: We agree that viral clusters play a crucial role in determining viral diversity, as highlighted by Camarillo-Guerrero et al., and we appreciate the reference to rBlast as a valuable tool in this context. However, the primary aim of PhageGE is to serve as a user-friendly web tool for rapid phylogenetic analysis and lifestyle prediction, particularly catering to users with limited programming experience. Additionally, PhageGE is designed to accelerate the translation of phage therapy into the clinic by providing phage phylogenetic and lifestyle information. As such, we have focused on providing an accessible and efficient platform for these specific purposes. While the inclusion of viral cluster analysis is beyond the current scope of PhageGE, we recognise its importance and potential benefits and will consider incorporating this feature in the next version of PhageGE.

4. On the "Phylogenetic analysis" landing page, I think "select phage whole genome data" should read "select phage genome data" as whole genome data would imply that phage particles were isolated and sequenced.

19 Innovation Walk
Monash University
VIC 3800, Australia
Telephone: (+61 3) 990 39702 Facsimile: (+61 3) 9905 6450 Email: Jian.Li@monash.edu
Web: https://research.monash.edu/en/persons/jian-li

Unintended recipient: please notify as soon as possible and destroy all pages received

Professor Jian Li
Fellow of the American Academy of Microbiology
Head, Laboratory of Antimicrobial Systems Pharmacology
Monash Biomedicine Discovery Institute

Response: We apologise for any confusion caused by the terminology on the "Phylogenetic analysis" landing page. We understand that "whole genome data" implies that phages were isolated and sequenced. To clarify, the primary function of PhageGE is to analyse assembled phage genomic data, which should use “phage whole-genome data” in the landing page as well as the usage description. To prevent any further misunderstanding, we have updated the description for PhageGE: "To demonstrate the functions and the scope of application of PhageGE, we herein describe the results of a case study using PhageGE, including phage whole-genome data (i.e., .fasta), a phylogenetic tree file (i.e., .tre), and genome annotation data (i.e., .xls, .txt and .gff), collectively referred to as “Example Data” (Figure 1)." (lines 105-108).

5. "This demonstrates that the phylogenetic analysis performance of PhageGE is accurate and comparable to the multiple sequence alignment-based approach." And "It has demonstrated the ability to accurately reconstruct biologically relevant phylogenies with thousands of microbial genomes [40-42]. The description of this function is briefly outlined below." How do phylogenies obtained using whole phage genomes (k-mer, ANI, or otherwise) compare to those reconstructed using the large terminase gene?

Response: We thank the reviewer for the insightful question regarding the comparison between phylogenies obtained from PhageGE and those reconstructed using the large terminase gene. Although both phylogeny analyses from whole phage genomes (k-mer based) and the large terminase gene can provide insights into phage diversity and evolution, there is a distinction. Whole-genome based analysis utilises the entire genomic content, capturing the full extent of genetic variation across the genome; while phylogeny reconstructed using a single gene (i.e. the large terminase gene) provides a narrower view of the phage's evolutionary history and potentially misses some genetic variations present. Furthermore, phages have the capability to lose or duplicate genes, including the large terminase gene, potentially leading to inaccuracies in phylogenetic inference (*Nat. Microbiol.*, 2017, 2(9), 1-9; *Nat. Rev. Microbiol.*, 2021, 15(3), 161-168). In contrast, k-mer based whole-genome phylogenies offer a comprehensive and high-resolution view of phage relationships, particularly valuable in distinguishing closely related phages and providing a more holistic view of their evolutionary relationships (*mBio*, 2017, 8(4), 10-1128). Therefore, we integrated a k-mer based whole phage genome phylogenetic analysis function into PhageGE to provide a high-resolution view of phage phylogeny for clinical translation.

19 Innovation Walk
Monash University
VIC 3800, Australia
Telephone: (+61 3) 990 39702 Facsimile: (+61 3) 9905 6450 Email: Jian.Li@monash.edu
Web: https://research.monash.edu/en/persons/jian-li

Unintended recipient: please notify as soon as possible and destroy all pages received

Professor Jian Li
Fellow of the American Academy of Microbiology
Head, Laboratory of Antimicrobial Systems Pharmacology
Monash Biomedicine Discovery Institute

6. "Furthermore, combining whole-genome sequencing (WGS) with in silico prediction enables rapid prediction of phage lifestyle [18]. Several popular bioinformatic pipelines and tools are available for such analyses, including MAFFT, RAxML and IQ-TREE (for multiple sequence alignment and phylogenetic analysis) [19-21], ggtree (for the visualisation of phylogeny data) [22], PHACTS and BACPHLIP (for phage lifestyle prediction) [18, 23]." What do each of the programs do? Perhaps restructure writing to reflect programs at higher-order groups. e.g., Several popular bioinformatic pipelines and tools are available for multiple sequence alignment (MAFFT), phylogenetic reconstruction (RAxML, IQ-TREE), visualisation of phylogeny (ggtree), and for phage lifestyle prediction (PHACTS, BACPHLIP).

Response: We thank the reviewer for the suggestion. The sentence has been restructured accordingly (lines 85-91).

7. "However, utilising these tools requires proficient programming skills, therefore, a biologist-friendly pipeline for phage genomic analyses is urgently needed to address the aforementioned limitations in phage genomic analysis." Its not entirely clear what the aforementioned limitations are. Are you referring to: "Optimising phage therapy in patients requires key pharmacological information, including infection cycle, gene content and phage taxonomy"

Response: The limitations refer to proficient programming skills required for phage genomic analysis when using these tools. We have clarified this point in the revised manuscript (lines 88-91).

General editorial revisions are required, some examples are given below:

Response: We thank the reviewer for the suggestions. In addition to the general editorial revisions suggested by the reviewer below, we have substantially revised the manuscript to improve grammar. Minor changes were not highlighted.

8. "To demonstrate the functions and application scope of PhageGE"
   To demonstrate the functions and the scope of application of PhageGE

Response: The sentence has been revised accordingly (line 105).

19 Innovation Walk
Monash University
VIC 3800, Australia
Telephone: (+61 3) 990 39702 Facsimile: (+61 3) 9905 6450 Email: Jian.Li@monash.edu
Web: https://research.monash.edu/en/persons/jian-li

Unintended recipient: please notify as soon as possible and destroy all pages received

Professor Jian Li
Fellow of the American Academy of Microbiology
Head, Laboratory of Antimicrobial Systems Pharmacology
Monash Biomedicine Discovery Institute

9. "This demonstrates that the phylogenetic analysis performance of PhageGE is accurate and comparable to the multiple sequence alignment-based approach."
   This demonstrates that the performance of the phylogenetic analysis of PhageGE is accurate and comparable to the multiple sequence alignment-based approach.

Response: The sentence has been revised accordingly (lines 142-144).

10. "Respectively" is used too frequently and creates confusing sentence constructions.
    e.g., "By selecting "common_annotation", a table with 75, 45, 51 genes that were annotated in all three pipelines were generated for KP36, vB8838 and FK1979, respectively. We also identified 17, 7 and 12 unique genes, respectively, from the Pharokka pipeline by selecting "Pharokka_only" option."

Response: We thank the reviewer for the suggestion. The second sentence above has been rewritten (lines 194-195).

11. "By employing an improved searching function (i.e. searching a sequence file against the build-in HMM [Hidden Markov Model] database)"
    By employing an improved search function (i.e. searching a sequence file against the built-in HMM [Hidden Markov Model] database)"

Response: The manuscript has been revised accordingly (line 323).

12. "To illustrate the phylogenetic analysis function in PhageGE, we employed our GitHub example dataset which consists of 14 phage genomes (Citrobacter, Escherichia, and Klebsiella) from 9 different genera (Figure 2A)."
    Need to make clear what the link between the 14 phage genomes to Citrobacter, Escherichia, and Klebsiella are. Are they 14 genomes of lytic phages that target Citrobacter, Escherichia, and Klebsiella? Or are they 14 phage sequences/genomes detected from bacterial isolate genomes of Citrobacter, Escherichia, and Klebsiella? I think a section describing the origin of data used would be helpful for readers.

Response: We thank the reviewer for the suggestion and have revised the manuscript accordingly (lines 112-121). All 15 phages are lytic phages that target *Citrobacter freundiifreundii* (2 phages), *Escherichia coli* (7 phages), and *Klebsiella pneumoniae* (6 phages).

19 Innovation Walk
Monash University
VIC 3800, Australia
Telephone: (+61 3) 990 39702 Facsimile: (+61 3) 9905 6450 Email: Jian.Li@monash.edu
Web: https://research.monash.edu/en/persons/jian-li

Unintended recipient: please notify as soon as possible and destroy all pages received

These 15 phage genomes were selected to demonstrate the application of PhageGE to a wide range of phages targeting clinically relevant pathogens. We included a *K. pneumoniae* phage, pKp20, and performed the phylogenetic analysis for this phage along with the other 14 phages. Notably, the taxonomic and lifestyle results of pKp20 contributed to a recent successful clinical case (*Antimicrob. Agents Chemother*., 2023, 67(4), e00037-23).

13. "To compare the results obtained from PhageGE with the multiple sequence alignment-based approach, we also conducted a multiple sequence alignment-based phylogenetic analysis using MAFFT v7.47 alongside the phylogenetic analysis conducted in PhageGE"
    What is the first MSA-based approached referring to here? I think the results section requires a brief overview of the steps executed within PhageGE to orientate the readers. This would provide a baseline understanding in an effort to facilitate the comparative narrative.

Response: We have revised the manuscript to clarify this point (lines 126-133). The MSA-based approach here refers to the phylogenetic analysis using MAFFT v7.47 and fasttree v2.1.10. We have also included a brief discussion on the performance of PhageGE in phylogenetic analysis with uploaded phage genomes.

14. "Its aim is to provide an interactive visualisation platform that improves the reusability of phylogenetic data and facilitates the phylogenetic analysis of phage comparative genomics studies." Reusability = reproducibility?

Response: This sentence has been changed to "...interactive visualisation platform that enhances the accessibility of phylogenetic data..." (line 147).

15. "Overall, all four functions from PhageGE serve as a guide for the exploration of phage genomic features and will expedite the clinical translation of phage therapy."
    The test data set requires more phage genomes that serve as positive and negative controls, including eukaryotic viruses. Table 2 phage lifecycle prediction needs controls for temperate phages, and non-phage viruses.

Response: We thank the reviewer for the suggestion and have included more phages (e.g. temperate phages) in the lifestyle prediction table (**Table 2**) to serve as positive (e.g. KP36 and pkp20) and negative (e.g. NC_017985 and NC_027339) controls (lines 176-180). Regarding the

19 Innovation Walk
Monash University
VIC 3800, Australia
Telephone: (+61 3) 990 39702 Facsimile: (+61 3) 9905 6450 Email: Jian.Li@monash.edu
Web: https://research.monash.edu/en/persons/jian-li

Unintended recipient: please notify as soon as possible and destroy all pages received

Professor Jian Li
Fellow of the American Academy of Microbiology
Head, Laboratory of Antimicrobial Systems Pharmacology
Monash Biomedicine Discovery Institute

inclusion of eukaryotic viruses, PhageGE is for genomic analyses of phages specifically, not non-phage viruses. We have also updated our current function to pop up an error message when non-phage viruses are detected: “The input is not from phage viruses”.

16. Figure legends require more descriptive text in order to assess.

Response: We thank the reviewer for the suggestion and have improved the figure legends accordingly.

17. Image quality of figures needs improvement, especially figure 5.

Response: All figures have been updated with a resolution of 300 dpi or higher.

18. Last sentence of first paragraph - upton = upon; Second paragraph - multi-omics has* the

Response: We apologise for the typographic errors and the manuscript has been revised accordingly (lines 78 and 80).

Reviewer2:

Major points:

1. It was seen that various annotation tools have been developed for phage genomes, and there are several works developed as integrated tools or pipelines for phage genome annotation and visualization. For example, Prophage Hunter (Song et al. 2019), Galaxy and Apollo (Ramsey et al. 2020), PhaGAA (Wu et al. 2023), … et al. However, the authors did not mention and discuss those works. Compared with those published works, PhageGE was designed with its functions some different from them, but still limited for the research community.

Response: We thank the reviewer for the comments regarding the comparison of PhageGE with other phage genome annotation and visualisation tools. In the revised manuscript we have clarified that PhageGE serves as a biologist-friendly interactive platform for phage genome analysis with a particular emphasis on phylogeny, lifestyle prediction, interactive phylogenetic tree visualisation, and annotation comparison (lines 92-98). The interactive visualisation capabilities of PhageGE are tailored to improve the accessibility and usability of phylogenetic data, facilitating comparative genomics studies and clinical translation within the phage research community.

19 Innovation Walk
Monash University
VIC 3800, Australia
Telephone: (+61 3) 990 39702 Facsimile: (+61 3) 9905 6450 Email: Jian.Li@monash.edu
Web: https://research.monash.edu/en/persons/jian-li

Unintended recipient: please notify as soon as possible and destroy all pages received

Professor Jian Li
Fellow of the American Academy of Microbiology
Head, Laboratory of Antimicrobial Systems Pharmacology
Monash Biomedicine Discovery Institute

Prophage Hunter is for studying active phages from whole genome assemblies of bacteria. The functionalities of PhageGE are designed to complement, rather than replicate, the capabilities of tools like Prophage Hunter.

The main annotation pipeline used in **Galaxy and Apollo** is PHANOTATE, which has been adapted into the Pharokka pipeline (*Bioinformatics*, 2023, 39(1), p.btac776). PhageGE focuses on integrating annotations into an interactive environment for comparative genome analysis and visualisation. Our approach enhances the utility of the annotations by providing a platform for deeper exploration and interpretation of phylogenetic relationships.

PhaGAA is an excellent online integrated platform for phage genome annotation and analysis, focusing on DNA/protein-based annotation, host prediction, and lifestyle reorganisation. The lifestyle reorganisation method in PhaGAA directly integrates PhaTYP (*Brief. Bioinform.*, 2023, 24(1), p.bbac487). The primary utility of PhaTYP is analysing phage lifestyle in human neonates' gut data, showcasing its value in studying phages in metagenomic contexts and enhancing our understanding of microbial communities.

In summary, PhageGE offers unique functionalities that complement existing tools, focusing on providing a biologist-friendly and specialised environment for phage genome analysis.

2. As pointed out above, PhageGE's functions were not comprehensive enough, especially did not address the characteristics of the host of bacteriophage or phage-host interaction which are important for phage genome studies. In addition, currently a tool like PhageGE would be expected to analyze metagenomic data with a large of short reads. Moreover, identification of resistance genes, analyzing potentially encoded resistance genes within the phage genome is crucial in phage genome analysis. So, adding analysis function of antibiotic resistance gene dissemination, examining genes related to antibiotic resistance in the phage genome, especially those that might affect host bacterial resistance through horizontal gene transfer, could greatly enhance the understanding of bacteriophages, their evolution, and host interactions if these analytical functions were integrated into the PhageGE pipeline.

Response: We appreciate the reviewer's valuable suggestions for enhancing PhageGE. We agree that understanding host characteristics and phage-host interactions are crucial; however, they are beyond the current scope of PhageGE. As mentioned in our response to Comment #1 above, PhageGE focuses on phylogenetic analysis and lifestyle prediction, aiming to expedite clinical

Professor Jian Li
Fellow of the American Academy of Microbiology
Head, Laboratory of Antimicrobial Systems Pharmacology
Monash Biomedicine Discovery Institute

translation of phage therapy (lines 116-121 and 176-177). This focus has led to a successful clinical case study (*Antimicrob. Agents Chemother*., 2023, 67(4), e00037-23).

Regarding antibiotic resistance gene (ARG) analysis, we recognise its critical role in understanding phage biology and their potential impact on bacterial resistance through horizontal gene transfer. Notably, recent studies have demonstrated that phages and prophages rarely carry ARGs, and bona fide ARGs attributed to phages in human- or mouse-associated viromes were previously overestimated due to bacterial DNA contamination and relaxed detection thresholds, leading to high false-positive rates (*ISME*, 2017, 11(1), 237-247; *ISME Commun.*, 2021, 1(1), 55). Nonetheless, we will consider incorporating this function in future versions of PhageGE.

3. As a presentation of an application, the authors provided limited cases with example datasets, and limited analysis.

Response: We thank the reviewer for the suggestion. In the revised manuscript we have included more example datasets to demonstrate each function (e.g., phylogenetic analysis and lifestyle prediction) (lines 112-121, 137-144, and 176-180). Moreover, we have demonstrated the application of functions from PhageGE using a clinical case study (lines 116-121 and 177-180).

Minor points:

4. The authors highlight in the background section the role of phage genome analysis in developing phage therapies. Therefore, it would be beneficial to demonstrate the application of this tool in case studies.

Response: We thank the reviewer for the suggestion. The manuscript has been revised to include a clinical case study (*Antimicrob. Agents Chemother*., 2023, 67(4), e00037-23) which demonstrates the application of phageGE (lines 112-121 and 176-180). This case study involved a recurrent urinary tract infection, and both taxonomy information from phylogeny analysis and the lifestyle prediction had played key roles in the phage selection.

5. While many offline tools for constructing phage evolutionary trees have been developed, a major disadvantage of a web tool is its lengthy runtime. The capacity of the tool to process a significant number of sequence data and the need for a runtime comparison should be addressed.

Response: We thank the reviewer for the suggestion. In the revised version we have included a

19 Innovation Walk
Monash University
VIC 3800, Australia
Telephone: (+61 3) 990 39702 Facsimile: (+61 3) 9905 6450 Email: Jian.Li@monash.edu
Web: https://research.monash.edu/en/persons/jian-li

Unintended recipient: please notify as soon as possible and destroy all pages received

Professor Jian Li
Fellow of the American Academy of Microbiology
Head, Laboratory of Antimicrobial Systems Pharmacology
Monash Biomedicine Discovery Institute

comparison of the PhageGE runtime with the MSA-based approach (lines 138-144). On a 2-GHz CPU with 64 GB RAM, PhageGE performed phylogenetic analysis for 15 and 146 phage genomes in 0.22 minutes and 4.42 minutes, respectively. In comparison, the MAS-based approach required more than 30 minutes and 296 minutes accordingly. Therefore, PhageGE offers superior computational and analysis efficiency.

6. The image resolution is too low, at only 144 dpi, insufficient for the required 300 dpi. Many characters in Figure 2A are unclear, suggesting a need for improved resolution.

Response: As per Reviewer 1, Point 17, all figures have been updated with a resolution of 300 dpi or higher.

7. The website http://phagege.com/ is not functioning and cannot be accessed.

Response: We have retested our current version and the url works properly.

19 Innovation Walk
Monash University
VIC 3800, Australia
Telephone: (+61 3) 990 39702 Facsimile: (+61 3) 9905 6450 Email: Jian.Li@monash.edu
Web: https://research.monash.edu/en/persons/jian-li

Unintended recipient: please notify as soon as possible and destroy all pages received
