# Supplementary material for: PhageGE: an interactive web platform for exploratory analysis and visualization of bacteriophage genomes
Source: Gigascience. 2024 Sep 25;13:giae074. doi: 10.1093/gigascience/giae074 (PMC11423353; doi:10.1093/gigascience/giae074)
Supplement: giae074_GIGA-D-24-00040_Revision_2 [file giae074_giga-d-24-00040_revision_2.pdf]

GigaScience

PhageGE: An interactive web platform for exploratory analysis and visualisation of bacteriophage genomes

--Manuscript Draft--

Manuscript Number:	GIGA-D-24-00040R2
Full Title:	PhageGE: An interactive web platform for exploratory analysis and visualisation of bacteriophage genomes
Article Type:	Technical Note
Funding Information:	Division of Microbiology and Infectious Diseases, National Institute of Allergy and Infectious Diseases (R21 AI156766) — Prof Jian Li
Abstract:	Background: Antimicrobial resistance is a serious threat to global health. Due to the stagnant antibiotic discovery pipeline, bacteriophages (phages) have been proposed as an alternative therapy for the treatment of infections caused by multidrug-resistant (MDR) pathogens. Genomic features play an important role in phage pharmacology. However, our knowledge of phage genomics is sparse and the use of existing bioinformatic pipelines and tools requires considerable bioinformatic expertise. These challenges have substantially limited the clinical translation of phage therapy.<br>Findings: A user-friendly graphical interface application, PhageGE (Phage Genome Explorer), was developed for the interactive analysis of phage genomes. The new R Shiny webserver, PhageGE, was designed for analysing phage whole-genome sequence (WGS) data. PhageGE integrates several existing R packages and combines them with several newly developed functions to perform phylogeny analysis and lifestyle prediction. The webserver offers several additional key functions, including interactive phylogenetic tree visualisation and annotation comparison. The output from PhageGE can be exported directly with publication-quality images.<br>Conclusions: PhageGE is a valuable tool for analysing phage genome data and may expedite the development and clinical translation of phage therapy. PhageGE is publicly available at http://phagege.com/.
Corresponding Author:	Jian Li<br>Monash Biomedicine Discovery Institute<br>AUSTRALIA
Corresponding Author Secondary Information:	
Corresponding Author's Institution:	Monash Biomedicine Discovery Institute
Corresponding Author's Secondary Institution:	
First Author:	Jinxin Zhao, Ph.D.
First Author Secondary Information:	
Order of Authors:	Jinxin Zhao, Ph.D.
	Jiru Han, Ph.D.
	Yu-Wei Lin, Ph.D.
	Yan Zhu, Ph.D.
	Michael Aichem
	Dimitar Garkov
	Phillip J. Bergen, Ph.D.
	Sue C. Nang, Ph.D.
	Jian-Zhong Ye, Ph.D.

Powered by Editorial Manager® and ProduXion Manager® from Aries Systems Corporation

	Tieli Zhou, Ph.D.
	Tony Velkov, Ph.D.
	Jiangning Song, Ph.D.
	Falk Schreiber, Ph.D.
	Jian Li, Ph.D.
**Order of Authors Secondary Information:**	
**Response to Reviewers:**	Reviewers' comments:<br>Reviewer1:<br>1.I think it's important to state upfront in the abstract what phageGE can do (i.e., phylogenetic analysis, visualisation of phylo tree, predict phage lifecycle, and comparative analysis of phage genome annotations).<br>Response: We thank the reviewer for the suggestion. We have revised the abstract accordingly (lines 49-59).<br>2.Please include a link to the phageGE webpage in the abstract.<br>Response: We thank the reviewer for the suggestion and have provided the webpage in the abstract (line 59).<br>3.Spelling mistakes throughout the manuscript - as an example L288: comprehensive<br>Response: We thank the reviewer for the suggestion and have carefully checked our manuscript (line 228).<br>4..fna and .fasta are the same format. i.e., fna is a type of FASTA file. fna specifies that the fasta file specifically contains nucleotides (instead of amino acids).<br>Response: We thank the reviewer for the suggestion and have revised our manuscript accordingly (lines 122, 590).<br>5.change "biologist friendly" to "User friendly" - this is more a philosophical/psychological change. I think bioinformatics on the command-line should be accessible and something biologists can develop. I think calling GUI programs "biologist-friendly" will deter more biologists from learning bioinformatics.<br>Response: We thank the reviewer for the suggestion. We have revised our manuscript accordingly, with changes made on lines 49, 89, 97, 219, and 261.
**Additional Information:**	
**Question**	**Response**
Are you submitting this manuscript to a special series or article collection?	No
**Experimental design and statistics**<br><br>Full details of the experimental design and statistical methods used should be given in the Methods section, as detailed in our Minimum Standards Reporting Checklist. Information essential to interpreting the data presented should be made available in the figure legends.<br><br>Have you included all the information requested in your manuscript?	Yes
**Resources**<br><br>A description of all resources used,	Yes

Powered by Editorial Manager® and ProduXion Manager® from Aries Systems Corporation

including antibodies, cell lines, animals and software tools, with enough information to allow them to be uniquely identified, should be included in the Methods section. Authors are strongly encouraged to cite Research Resource Identifiers (RRIDs) for antibodies, model organisms and tools, where possible.<br><br>Have you included the information requested as detailed in our Minimum Standards Reporting Checklist?	
**Availability of data and materials**<br><br>All datasets and code on which the conclusions of the paper rely must be either included in your submission or deposited in publicly available repositories (where available and ethically appropriate), referencing such data using a unique identifier in the references and in the "Availability of Data and Materials" section of your manuscript.<br><br>Have you have met the above requirement as detailed in our Minimum Standards Reporting Checklist?	Yes

Manuscript

Click here to access/download;Manuscript;GIGA-D-24-00040R1 PhageGE_main.docx

PhageGE: An interactive web platform for exploratory analysis and visualisation of bacteriophage genomes

Jinxin Zhao[1, 2*], Jiru Han[3], Yu-Wei Lin[1], Yan Zhu[1, 4], Michael Aichem[5], Dimitar Garkov[5], Phillip J. Bergen[1], Sue C. Nang[1], Jian-Zhong Ye[6, 7], Tieli Zhou[6, 7], Tony Velkov[8], Jiangning Song[2, 9], Falk Schreiber[5, 10], Jian Li[1, 2*]

[1] Infection Program and Department of Microbiology, Biomedicine Discovery Institute, Monash University, Clayton, VIC, Australia

[2] Monash Biomedicine Discovery Institute-Wenzhou Medical University Alliance in Clinical and Experimental Biomedicine, Monash University, Clayton, VIC, Australia

[3] Population Health and Immunity Division, The Walter and Eliza Hall Institute of Medical Research, Parkville, VIC, Australia

[4] Systems Biology Center, Tianjin Institute of Industrial Biotechnology, Chinese Academy of Sciences, Tianjin, China

[5] Department of Computer and Information Science, University of Konstanz, Konstanz, Germany

[6] Key Laboratory of Clinical Laboratory Diagnosis and Translational Research of Zhejiang Province, Department of Clinical Laboratory, The First Affiliated Hospital of Wenzhou Medical University, Zhejiang, China

[7] Wenzhou Medical University-Monash Biomedicine Discovery Institute Alliance in Clinical and Experimental Biomedicine, The First Affiliated Hospital of Wenzhou Medical University,Wenzhou, China

[8] Department of Pharmacology, Biomedicine Discovery Institute, Monash University, Melbourne, VIC, Australia

[9] Department of Biochemistry and Molecular Biology, Biomedicine Discovery Institute, Monash University, Melbourne, VIC, Australia

[10] Faculty of Information Technology, Monash University, Clayton, VIC, Australia

Email addresses: JZ, jinxin.zhao@monash.edu; JH, han.ji@wehi.edu.au; YL, yu-wei.lin@monash.edu; YZ, Yan.Zhu@monash.edu; MA, michael.aichem@uni-konstanz.de; DG, dimitar.garkov@uni-konstanz.de; PB, phillip.bergen@monash.edu; SN, sue.nang@monash.edu; JY, jzye89@163.com; TZ, wyztli@163.com; TV, tony.velkov@monash.edu; JS, jiangning.song@monash.edu; FS, falk.schreiber@uni-konstanz.de; JL, jian.li@monash.edu.

Running title: PhageGE for bacteriophage genomic analysis

*Corresponding authors:

Dr Jinxin Zhao, Tel: +61 3 99056288, Email: jinxin.zhao@monash.edu;

Professor Jian Li, Tel: +61 3 99039172 Fax: +61 0399056450; Email: jian.li@monash.edu.

Abstract

Background: Antimicrobial resistance is a serious threat to global health. Due to the stagnant antibiotic discovery pipeline, bacteriophages (phages) have been proposed as an alternative therapy for the treatment of infections caused by multidrug-resistant (MDR) pathogens. Genomic features play an important role in phage pharmacology. However, our knowledge of phage genomics is sparse and the use of existing bioinformatic pipelines and tools requires considerable bioinformatic expertise. These challenges have substantially limited the clinical translation of phage therapy. **Findings:** We have developed PhageGE (Phage Genome Explorer), a user-friendly graphical interface application for the interactive analysis of phage genomes. PhageGE enables users to perform key analyses, including phylogenetic analysis, visualisation of phylogenetic trees, prediction of phage lifecycle, and comparative analysis of phage genome annotations. The new R Shiny webserver, PhageGE, integrates existing R packages and combines them with several newly developed functions to facilitate these analyses. Additionally, the webserver provides interactive visualisation capabilities and allows users to directly export publication-quality images.

Conclusions: PhageGE is a valuable tool that simplifies the analysis of phage genome data and may expedite the development and clinical translation of phage therapy. PhageGE is publicly available at http://www.phagege.com/.

Keywords: Phage genome, biological web application, genomic analysis, phylogeny, lifestyle

Introduction

The rapid emergence and spread of antimicrobial resistance (AMR) is one of the three greatest threats to human health globally [1]. It is estimated that by 2050, life-threatening infections caused by antimicrobial-resistant pathogens will kill more people than any other diseases [2]. Of particular concern is the increased prevalence of infections caused by Gram-negative pathogens, which are more difficult to treat than Gram-positive pathogens [3]. Given the sluggish global antibiotic pipeline [4], bacteriophages (phages) have attracted significant attention over the last decade as a potential alternative therapy for bacterial infections [5]. Phages are bacterial viruses and the advantages of phage therapy over antibiotics include a narrow spectrum of activity, the capacity to multiply at the infection site, and safety [6-8]. Optimising phage therapy in patients requires key pharmacological information, including infection cycle, gene content, and phage taxonomy [9, 10]. For example, temperate phages do not immediately lyse bacterial host cells and have an inherent capacity to mediate the transfer of genes between bacteria, potentially facilitating increased bacterial virulence and AMR. In contrast, lytic phages kill bacteria upon infection and are commonly used for the treatment of MDR bacterial infections in patients [11-14].

Multi-omics has the potential to expedite the clinical translation of phage therapy for the treatment of MDR bacterial infections [15]. For example, whole genome-based phylogenetic analysis offers significant advantages in understanding phage evolutionary dynamics and designing potential phage cocktails [16, 17]. Furthermore, combining whole-genome sequencing (WGS) with *in silico* prediction enables rapid prediction of phage lifestyle [18]. Several popular bioinformatic pipelines and tools are available for multiple sequence alignment (MAFFT) [19], phylogenetic reconstruction (RAxML and IQ-TREE) [20, 21], visualisation of phylogeny (ggtree) [22], and phage

lifestyle prediction (PHACTS and BACPHLIP) [18, 23]; however, utilising these tools requires proficient programming skills. Therefore, a user-friendly platform for phage genomic analyses is urgently needed to overcome the challenges associated with the requirement for advanced programming expertise.

Here, we developed an integrated webserver platform, PhageGE, that offers four key functionalities: phage phylogenetic analysis, tree visualisation, lifestyle prediction, and manipulation of phage genome annotation datasets. PhageGE differs from existing phage genomic analysis tools in that it facilitates the seamless export of all associated results in a publication-ready format without requiring complex procedures or long running times. Overall, PhageGE provides a user-friendly interface to streamline phage genomic analysis with WGS data.

Results

The PhageGE webserver (biotoolsID: biotools:phagege and RRID: SCR_025380) was designed to ensure user-friendliness and compatibility with major web browsers, including Google Chrome, Mozilla Firefox, Apple Safari, and Microsoft Edge (**Table 1**).

Webserver submission and case studies

To demonstrate the functions and the scope of application of PhageGE, we herein describe the results of a case study using PhageGE, including phage whole genome data (i.e., .fasta), a phylogenetic tree file (i.e., .tre), and genome annotation data (i.e., .xls, .txt and .gff), which are collectively referred to as “Example Data” (**Figure 1**). The complete set of Example Data used in the case studies can be accessed on the PhageGE GitHub repository (https://github.com/JinxinMonash/PhageGE).

Phage phylogenetic analysis and visualisation

To illustrate the phylogenetic analysis function in PhageGE and its application in clinical translation, we analysed our GitHub example dataset, which consists of 15 phage genomes. The hosts of the 15 phage genomes in the phylogenetic analysis are from 3 different bacterial species: *Citrobacter freundii, Escherichia coli, and Klebsiella pneumoniae* (**Figure 2A**). This dataset includes one anti-Klebsiella phage, pKp20, which was isolated in our lab and used in a clinical case [24]. In that case, a recurrent urinary tract infection [rUTI] was successfully treated with 4 weeks of adjunctive intravenous bacteriophage therapy, with no recurrence during a year of follow-up [24]. Both taxonomy information from phylogeny analysis and the lifestyle prediction played key roles in the selection of pKp20 over a wide range of phages [24]. The phage WGS data in the fasta format can be obtained either from NCBI or prepared locally using standard genome assembly pipelines (e.g., SPAdes) based on the previous BLASTn result [24]. To compare the results obtained from PhageGE with the multiple sequence alignment-based approach, we also conducted a multiple sequence alignment-based phylogenetic analysis using MAFFT v7.47 and fasttree v2.1.10, alongside the phylogenetic analysis using PhageGE. We firstly uploaded the selected fasta files or a multi-fasta file which contains all phage genomes on the Phylogenetic Analysis page in PhageGE, then selected the layout of the tree (i.e., phylogram, cladogram, fan, radial, or tidy) and clicked the “Explore Tree” icon. The resulting phylogenetic tree, representing the relationships among the uploaded genomes, was generated using the built-in *k*-mer-based alignment-free phylogenetic approach, as detailed in the Methods section (**Figures 2A and 3A**). To enhance the clarity, we manually highlighted the 15 phages with distinct colours according to their genus. Comparison of the phylogenetic trees generated by PhageGE and MAFFT revealed that both trees

shared largely the same classification (e.g., positions of each phage and the related taxa) (**Figure 3**). Moreover, PhageGE demonstrates a significant improvement in runtime efficiency. For example, on a 2-GHz CPU with 64 GB RAM server, the runtimes of generating phylogenetics trees by PhageGE were 0.22 minutes for 15 phage genomes and 4.42 minutes for 146 phage genomes. In contrast, the MSA-based approach (using tools like MAFFT along with FastTree) took 30 minutes and 296 minutes, respectively. This demonstrates that the performance of the phylogenetic analysis of PhageGE is accurate, fast and comparable to the multiple sequence alignment-based approach.

The phylogenetic visualisation function handles the phylogenetic tree along with diverse accompanying data. Its aim is to provide an interactive visualisation platform that enhances the accessibility of phylogenetic data and facilitates the phylogenetic analysis of phage comparative genomics studies. The phylogenetic tree and associated data can be extracted using a built-in function within PhageGE. This function is illustrated using a tree file "phage.tre" obtained from phage phylogenetic analysis (whether generated by PhageGE or other phylogenetic analysis pipeline) and a sample information file named "sample_info.csv" containing the taxonomy information for all 14 phages (**Figure 2B**). As shown in **Figure 4**, each dot in the dendrogram represents one phage with the colour indicating its taxonomic classification in the same genus. In addition, detailed information of each phage (e.g., name and taxonomy) can be easily accessed by hovering the cursor over the dot of interest (as indicated by the pink box in **Figure 4**). This interactive feature allows users to dynamically integrate and visualise the underlying information in a user-friendly manner.

Performance of phage lifestyle prediction

The lifestyle prediction function builds on a Random Forest classifier that incorporates up-to-date conserved protein domains with the ability to classify temperate and lytic phages using WGS data. To evaluate its performance, we compared the function with other published tools using the dataset of 1,057 phages in the literature [25]. The PhageGE lifestyle prediction function achieved the lowest error rates (0%, 1.2%, 0.3% and 2.5%, equivalent to 100%, 98.8%, 99.7% and 97.5% classification accuracy, respectively) across all tested datasets, substantially outperforming those existing tools for phage lifestyle classification (**Figure 5**). The prediction accuracy of PhageGE exceeded that of the most accurate existing tool, BACPHLIP, which had prediction accuracies of 99.8%, 98.3%, 99.2% and 96.5%, respectively (**Figure 5**). Similarly, WGS data for individual phages (e.g., *Klebsiella* phage KP36.fasta, vB8388.fasta, and FK1979.fasta from the example dataset described here) can be uploaded as input to generate the phage lifestyle probability table (**Figure 2C** and **Table 2**). The result presented in **Table 2** predicts that *Klebsiella* phages KP36 (a model phage in our laboratory), FK1979, and vB8388 [26] (two phages isolated from hospital sewage, The First Affiliated Hospital of Wenzhou Medical University, China), and pKp20 (used in the rUTI clinical case study) [24], are highly likely lytic phages, with the probability of 99.3%, 95.6% and 96.9%, respectively. Meanwhile, the four phages from the NCBI in **Table 2** NC_017985, NC_027339, NC_009815, and NC_019768 are highly likely temperate phages. This function empowers users to rapidly analyse the lifestyle of a phage of interest *in silico* with high prediction accuracy, providing key insights into the intricate phage ecosystems and enabling optimal design of phage therapy.

Comparison of phage genome annotation

Notably, PhageGE also provides a function to compare phage genome annotations obtained from different pipelines (i.e., Pharokka, Phaster and RAST). This analysis

involves the integration of R package flextable, which allows for the generation of downloadable comparison results in multiple formats (e.g., csv, Excel and PDF). The user interface offers the flexibility to rank the results based on multiple parameters (e.g., location and/or length of the coding sequence [CDS]). In the case study presented here, we used PhageGE to compare genome annotations of *Klebsiella* phages KP36, vB8838, and FK1979 generated from Phaster, RAST, and Pharokka (**Figure 2D**). By selecting “common_annotation”, a table with 75, 45, and 51 genes that were annotated in all three pipelines was generated for KP36, vB8838, and FK1979, respectively. We also identified 17, 7, and 12 unique genes from the Pharokka pipeline by selecting the “Pharokka_only” option. To gain a better understanding of those unique annotated genes, PhageGE allows users to directly copy and download both the nucleotide and amino acid sequences associated with the genes from the interactive table. This feature facilitates further investigation of these unique annotations.

Discussion

With the dramatic rise in MDR bacterial infections, phage therapy has emerged as a safe and potentially effective alternative treatment option to antibiotics [27]. However, the development of effective phage therapies is complex, involving the isolation, culturing, characterisation, and timely preparation of efficacious phages. Traditionally, this process is time-consuming and costly [28, 29]. Nevertheless, with the next-generation sequencing techniques, it has become possible to rapidly and cost-effectively characterise phages. Despite this advancement, there is a paucity of intuitive tools available for phage genomics, with the majority requiring operation in

command-line mode. The availability of large phage genomic datasets presents unique opportunities to develop bioinformatics tools that aid in phage biology and pharmacology research. The use of computational methods to study phages has shown promise in generating novel insights, such as phylogeny and lifestyle, through bioinformatic analysis [18, 25, 30]. However, there is currently no single tool available that encompasses all those functions (e.g., phylogenetic analysis, tree visualisation, lifestyle prediction, and genome annotation comparison) in the webserver platform. Herein, we describe the development of the PhageGE webserver GUI streamlined for user-friendly phage genomic analysis.

PhageGE is a novel, user-friendly GUI application for the interactive analysis of phage genomes. The overarching goal of PhageGE is to provide an interactive analysis and visualisation platform for the rapid exploration of phage genomic associations, thereby promoting efficient genomic data-driven discovery of phage therapy. PhageGE comprises a set of functions for phage genomic analysis, including phylogenetic analysis, tree visualisation, lifestyle prediction, and genome annotation comparison. While current tools like PhaGAA can provide lifestyle reorganisation analysis, their primary utility lies in analysing phage lifestyle for their prefered phage dataset (e.g., gut flora of human neonates) [31]. In contrast, PhageGE integrates a more comprehensive dataset with a wide range of phage genomes, allowing for broader and deeper exploration of phage lifestyles. Moreover, the comparsion of annotations from different pipelines highlights the key role of PhageGE in advancing phage genomics through enhanced analysis and visualisation functions. To exemplify the utility of PhageGE, we investigated the phylogeny, lifestyle, and annotation comparison of *Klebsiella* phages KP36, vB8838, and FK1979, which were independently isolated in two different countries. Our findings demonstrate that the various functions of

PhageGE yield comparable or better results than existing state-of-the-art approaches. These results highlight the significant potential of PhageGE in analysing various phage genomic features using phage WGS data.

Notably, PhageGE requires only phage WGS data as the input for conducting the related analysis. The phage phylogenetic analysis function takes phage WGS in the fasta format as input and applies an alignment-free phylogenetic approach to infer phylogenetic relationships. Compared to current phylogenetic analysis pipelines (i.e., multiple sequence alignment-based phylogenetic analysis), analysis from PhageGE showed similar phage phylogeny information in a shorter computing time (approximately 13 seconds versus 30 minutes for 15 phage genomes). Moreover, the result from phylogenetic analysis can be easily exported in various graphical formats (e.g., SVG, PDF and JPEG) and textual formats (e.g., Newick and Nexus) and can be interactively managed and viewed through our designed user interface. In addition, PhageGE introduces an enhanced phage lifestyle prediction function, using a machine-learning approach with updated databases for conserved protein domains. The overall approaches applied for both phylogenetic analysis and lifestyle prediction demonstrate that analyses results from PhageGE are comparable to previously published tools (**Figures 3** and **5**), showing its effectiveness in accurately analysing phage phylogeny and predicting phage lifestyle**.** Notably, PhageGE incorporates a function of annotation comparison to facilitate the efficient organisation of genome annotation files derived from different annotation pipelines. This feature allows users to efficiently compare genome annotation data obtained with different tools. Overall, all fours functions from PhageGE serve as a guide for the exploration of phage genomic features and will expedite the clinical translation of phage therapy.

Conclusion

In conclusion, PhageGE is the first user-friendly tool for the analysis of phage genomes, offering improved functions compared to existing tools without the need for considerable programming skills. Uniquely incorporating features like phylogenetic analysis, interactive tree visualisation, lifestyle prediction, and genome annotation comparison, we anticipate that PhageGE will become an instrumental bioinformatic web server for phage genomic analysis, guiding experimental validations and advancing the development of phage therapy.

Methods

Implementation

PhageGE 1.0 was developed in R and is hosted on Shinyapps. This application seamlessly integrates various R packages, including Rshiny, seqinr, Biostrings, ape, textmineR, tidyverse, ggtree, ploty, ggplot, reticulate, and pyhmmer [22, 32-38]. Furthermore, it incorporates several key functions, including *k*-mer-based phylogeny estimation, phylogenetic tree visualisation, lifestyle prediction, and annotation comparison. To use PhageGE, input files in the standard WGS fasta format are required, along with textual tables in standard formats (e.g., csv or xlsx) containing sequence details and annotation information. The workflow is illustrated in **Figure 1**.

Phage genomic analysis pipeline

The functionalities offered in the web interface of PhageGE utilise WGS fasta files for phylogenetic analysis and lifestyle prediction. Users can input tree files (e.g., Newick or Nexus) and textual files (i.e., csv or xlsx) for phylogenetic tree visualisation and

genome annotation comparisons. Using these standard formats as input files facilitates effective use and simplifies data export for users.

Phylogenetic analysis and phylogenetic tree visualisation

The phylogenetic analysis function enables fast and efficient analysis of phage phylogeny. It includes phylogeny reconstruction based on the input WGS data and visualisation of phylogenetic information. This function incorporates a *k*-mer-based alignment-free phylogenetic approach [39]. Alignment-free phylogenetic approaches offer a scalable alternative for inferring phylogenetic relationships and computing local alignment boundaries from WGS data [40, 41]. This approach is particularly robust for genome sequences that exhibit genetic recombinations and rearrangements. It has demonstrated the ability to accurately reconstruct biologically relevant phylogenies with thousands of microbial genomes [42-44]. The description of this function is briefly outlined below.

Consider a sequence consisting of four characters (A, T, C, G) of length k (‘*k*-mer’), described by **Equation 1**. There are 4^k possible *k*-mers (**Equation 2**), which can serve as features of each genome. The value assigned to a specific *k*-mer feature will correspond to the number of occurrences of that *k*-mer in the genome. Using these *k*-mer features, a data matrix is generated with dimensions of the numbers of genomes of interest (n columns) by 4^k rows. To establish a representative probability distribution of the 4^k *k*-mers, each row of the data matrix is normslised by its row total. This normalisation results in a feature-frequency profile (F_k, described by **Equation 3**) for each *k*-mers sequence [39]. The Jensen-Shannon divergence (D_k, described by **Equation 4**) is then employed to estimate the genome pairwise distances [45]. Subsequently, the resulting distance matrix is used as an input for a clustering

algorithm (e.g., neighbor-joining algorithm) to summarise the relatedness of the phage genomes and construct a phylogenetic tree [35].

Equation 1: $C_k = < C_{k,1}, C_{k,2} \cdots C_{k,m} >$

Equation 2: $m = 4^k$

Equation 3: $F_{n_i,k} = \frac{C_{n_i,k_m}}{\sum_{n_i} C_{n_i,k}}$

Equation 4: $D_k = JS(F_{n_1,k}, F_{n_i,k})$

An interactive visualisation of a phylogenetic tree was generated either from the phylogenetic analysis function or a customised phylogenetic tree that includes additional information, such as species classification, duplication events, and bootstrap values. It is implemented using ggtree and ploty R packages [22], ensuring the ability to handle most common tree formats (e.g., Newick, Nexus, and tre).

Lifestyle prediction

The Lifestyle Prediction function in PhageGE generates a phage lifestyle probability table based on the input of phage WGS data. This function adapted previously reported approaches into our user-friendly interface [18, 23, 25]. By employing an improved search function (i.e. searching a sequence file against the build-in Hidden Markov Model [HMM] database), PhageGE provides an efficient way to predict phage lifestyle based on the phage genomic information.

In brief, we first conducted a search in the Conserved Domain Database (accessed: 11/2023) to collect protein domains from temperate phages [46]. The following key

words were used to identify relevant protein domains: ‘temperate’, ‘lysogen’, ‘integrase’, ‘excisionase’, ‘recombinase’, ‘transposase’, ‘parA|parB’, and ‘xerC|xerD’. We obtained a total of 477 protein domains from the initial collection, which were then subjected to a careful manual curation and filtration (e.g., minimal domain length >30 and validated in the existing experimental data), resulting in a refined set of 261 protein domains. Next, a lifestyle classification model was trained and tested using a published dataset consisting of 1,057 phages from 6 different families (*Inoviridae*, *Myoviridae*, *Plasmaviridae*, *Podoviridae*, *Siphoviridae*, and *Tectiviridae*) across 55 host genera, with known genome and lifestyle information [25]. The dataset was randomly split into training and testing sets, with a ratio of 60:40 (634 phages in the training set and 423 phages in the testing set). At this stage, the testing set was fully set aside for subsequent descriptions related to model training and development. For each genome sequence in the training set, we generated a list of all possible 6-frame translation sequences that were at least 40 amino acids long. HMMER3 was then used to search for the presence or absence of the various protein domains listed above, resulting in a vector for each phage describing the presence (1) or absence (0) of each domain [47]. This information allowed us to filter the initial set of 477 putatively useful protein domains down to the final set of 261. Subsequently, a Random Forest classifier was fitted to the training set of phage genomes, and cross-validation was employed to fine-tune the model hyper-parameters. The ‘best’ performing model was then selected by choosing the hyper-parameters that yielded the highest minimum accuracy across the independent validation set tests. The parameters of that model were then re-fitted to the entire training set data, resulting in the final model.

Annotation comparison

The Rapid Annotation using Subsystem Technology (RAST) server was developed in 2008 to annotate microbial genomes based on the manually curated SEED database [48]. The PHAge Search Tool – Enhanced Release (PHASTER) was specifically designed to identify and annotate prophage sequences within bacteria using prophage/virus databases [49]. More recently, another phage annotation tool, Pharokka, has been developed using PHROGS, CARD, and VFDB databases [50]. Since these pipelines employ different databases for phage genome annotation, it is possible to obtain different annotations from each pipeline. To provide more comprehensive annotation results, there is an urgent need for annotation comparison tables that incorporate all annotation information from RAST, PHASTER, and Pharokka. The Annotation Comparison function in PhageGE generates interactive tables that display comments and differing genome annotation information obtained from RAST, PHASTER, and Pharokka. This comparison includes checking the coding regions and related annotations from each pipeline. Moreover, it provides an overview of common and different annotation counts, facilitating the tracking of differences between the three pipelines. This function is implemented using the flextable, tidyselect, data.table, and tidyverse packages [37].

Code availability and requirements

- Project name: PhageGE (Phage Genome Exploration)
- Project homepage: https://github.com/JinxinMonash/PhageGE
- Operating system(s): Linux, Windows and MacOS (**Table 1**)
- Programming language: R
- License: MIT license

Data availability

In general, all data used in this work were from openly accessible public repositories and released with other publications under open-source licenses. The data used were solely for research purposes, and we confirm that they were not used for any other noncommercial or commercial purpose. The datasets supporting the results of this article are available in the Github repository, [https://github.com/JinxinMonash/PhageGE]. The data used as examples can be found in the release branch called “Example data” or “Example data.zip” within our repository.The GitHub repository also contains up-to-date tutorials.

Competing interests

The authors declare that they have no competing interests.

Funding

This work was supported by the National Institute of Allergy and Infectious Diseases of the National Institutes of Health [grant number R21 AI156766 to J.L.]. The content is solely the responsibility of the authors and does not necessarily represent the official views of the National Institute of Allergy and Infectious Diseases or the National Institutes of Health.

Author’s contributions

J.Z. collected all the data and participated in developing the webserver and writing the manuscript. J.H., Y.W.L., Y.Z., M.A. and D.G. and J.N.S. contributed to the

development of the web server. P.J.B., S.N., J.Z.Y., T.L.Z. and T.V. took part in the discussion of the data. J.Z., F.S. and J.L. conceived the study, coordinated the work and contributed to writing the manuscript. All authors are involved in the discussion and finalisation of the manuscript.

Acknowledgements

J.Z. is a recipient of the 2022 Faculty of Medicine, Nursing and Health Sciences Bridging Fellowship, Monash University. J.L. is an Australian National Health Medical Research Council (NHMRC) Investigator Research Fellow and T.V. is an Australian Research Council (ARC) Industrial Fellow. Y.W.L. is currently an employee of Certara, Australia and Co-Director of the Malaya Translational and Clinical Pharmacometrics Group, University of Malaya, Malaysia.

References

1. Murray CJ, Ikuta KS, Sharara F, Swetschinski L, Aguilar GR, Gray A, et al. Global burden of bacterial antimicrobial resistance in 2019: a systematic analysis. The Lancet. 2022;399 10325:629-55.
2. Luepke KH, Suda KJ, Boucher H, Russo RL, Bonney MW, Hunt TD, et al. Past, present, and future of antibacterial economics: increasing bacterial resistance, limited antibiotic pipeline, and societal implications. Pharmacotherapy: The Journal of Human Pharmacology and Drug Therapy. 2017;37 1:71-84.
3. Bassetti M and Garau J. Current and future perspectives in the treatment of multidrug-resistant Gram-negative infections. Journal of Antimicrobial Chemotherapy. 2021;76 (Suppl 4):iv23-iv37.
4. Böttcher L, Gersbach H and Wernli D. Restoring the antibiotic R&D market to combat the resistance crisis. Science and Public Policy. 2022;49 1:127-31.
5. Uyttebroek S, Chen B, Onsea J, Ruythooren F, Debaveye Y, Devolder D, et al. Safety and efficacy of phage therapy in difficult-to-treat infections: a systematic review. The Lancet Infectious Diseases. 2022; 22 8:E208-E220.
6. Kortright KE, Chan BK, Koff JL and Turner PE. Phage therapy: a renewed approach to combat antibiotic-resistant bacteria. Cell Host & Microbe. 2019;25 2:219-32.
7. Mousavi SM, Babakhani S, Moradi L, Karami S, Shahbandeh M, Mirshekar M, et al. Bacteriophage as a novel therapeutic weapon for killing colistin-resistant multi-drug-resistant and extensively drug-resistant gram-negative bacteria. Current Microbiology. 2021;78 12:4023-36.
8. Lin Y, Chang RY, Rao G, Jermain B, Han M-L, Zhao J, et al. Pharmacokinetics/pharmacodynamics of antipseudomonal bacteriophage

therapy in rats: a proof-of-concept study. Clinical Microbiology and Infection. 2020;26 9:1229-35.

9. Hyman P. Phages for phage therapy: isolation, characterization, and host range breadth. Pharmaceuticals. 2019;12 1:35.

10. Gordillo Altamirano FL and Barr JJ. Phage therapy in the postantibiotic era. Clinical Microbiology Reviews. 2019;32 2:e00066-18.

11. Roach DR, Leung CY, Henry M, Morello E, Singh D, Di Santo JP, et al. Synergy between the host immune system and bacteriophage is essential for successful phage therapy against an acute respiratory pathogen. Cell Host & Microbe. 2017;22 1:38-47. e4.

12. Harrison E and Brockhurst MA. Ecological and evolutionary benefits of temperate phage: what does or doesn't kill you makes you stronger. BioEssays. 2017;39 12:1700112.

13. Gill JJ and Hyman P. Phage choice, isolation, and preparation for phage therapy. Current Pharmaceutical Biotechnology. 2010;11 1:2-14.

14. Abedon ST, García P, Mullany P and Aminov R. Phage therapy: past, present and future. Frontiers Media SA, 2017, p. 981.

15. Debnath M, Prasad GB and Bisen PS. Omics technology. Molecular Diagnostics: Promises and Possibilities. Springer; 2010. p. 11-31.

16. Parmar KM, Dafale NA, Tikariha H and Purohit HJ. Genomic characterization of key bacteriophages to formulate the potential biocontrol agent to combat enteric pathogenic bacteria. Archives of Microbiology. 2018;200 4:611-22.

17. Philipson CW, Voegtly LJ, Lueder MR, Long KA, Rice GK, Frey KG, et al. Characterizing phage genomes for therapeutic applications. Viruses. 2018;10 4:188.

18. McNair K, Bailey BA and Edwards RA. PHACTS, a computational approach to classifying the lifestyle of phages. Bioinformatics. 2012;28 5:614-8.
19. Katoh K and Standley DM. MAFFT multiple sequence alignment software version 7: improvements in performance and usability. Molecular Biology and Evolution. 2013;30 4:772-80.
20. Stamatakis A. RAxML version 8: a tool for phylogenetic analysis and post-analysis of large phylogenies. Bioinformatics. 2014;30 9:1312-3.
21. Minh BQ, Schmidt HA, Chernomor O, Schrempf D, Woodhams MD, Von Haeseler A, et al. IQ-TREE 2: new models and efficient methods for phylogenetic inference in the genomic era. Molecular Biology and Evolution. 2020;37 5:1530-4.
22. Xu S, Li L, Luo X, Chen M, Tang W, Zhan L, et al. Ggtree: A serialized data object for visualization of a phylogenetic tree and annotation data. iMeta. 2022;3 1:e56.
23. Hockenberry AJ and Wilke CO. BACPHLIP: predicting bacteriophage lifestyle from conserved protein domains. PeerJ. 2021;9:e11396.
24. Le T, Nang SC, Zhao J, Yu HH, Li J, Gill JJ, et al. Therapeutic potential of intravenous phage as standalone therapy for recurrent drug-resistant urinary tract infections. Antimicrobial Agents and Chemotherapy. 2023;67 4:e00037-23.
25. Mavrich TN and Hatfull GF. Bacteriophage evolution differs by host, lifestyle and genome. Nature Microbiology. 2017;2 9:1-9.
26. Zhao Y, Feng L, Zhou B, Zhang X, Yao Z, Wang L, et al. A newly isolated bacteriophage vB8388 and its synergistic effect with aminoglycosides against

multi-drug resistant *Klebsiella oxytoca* strain FK-8388. Microbial Pathogenesis. 2023;174:105906.

27. Khan A, Rao TS and Joshi HM. Phage therapy in the Covid-19 era: Advantages over antibiotics. Current Research in Microbial Sciences. 2022;3:100115.

28. Camarillo-Guerrero LF, Almeida A, Rangel-Pineros G, Finn RD and Lawley TD. Massive expansion of human gut bacteriophage diversity. Cell. 2021;184 4:1098-109.

29. Carrigy NB, Larsen SE, Reese V, Pecor T, Harrison M, Kuehl PJ, et al. Prophylaxis of *Mycobacterium tuberculosis* H37Rv infection in a preclinical mouse model via inhalation of nebulized bacteriophage D29. Antimicrobial Agents and Chemotherapy. 2019;63 12:e00871-19.

30. Martinez-Vaz BM and Mickelson MM. In silico phage hunting: bioinformatics exercises to identify and explore bacteriophage genomes. Frontiers in Microbiology. 2020;11:577634.

31. Wu J, Liu Q, Li M, Xu J, Wang C, Zhang J, et al. PhaGAA: an integrated web server platform for phage genome annotation and analysis. Bioinformatics. 2023;39 3:btad120.

32. Sievert C. Interactive web-based data visualization with R, plotly, and shiny. Journal of the Royal Statistical Society Series A: Statistics in Society. 2020;184:1150.

33. Charif D and Lobry JR. SeqinR 1.0-2: a contributed package to the R project for statistical computing devoted to biological sequences retrieval and analysis. Structural approaches to sequence evolution. Springer; 2007. p. 207-32.

34. Pagès H, Aboyoun P, Gentleman R, DebRoy S. Biostrings: Efficient manipulation of biological strings. 2024. Biostrings (Version 2.70.2) https://bioconductor.org/packages/Biostrings.

35. Paradis E and Schliep K. ape 5.0: an environment for modern phylogenetics and evolutionary analyses in R. Bioinformatics. 2019;35 3:526-8.

36. Jones T, Doane W and Jones MT. Package 'textmineR'. Functions for text mining and topic modeling. 2021. textmineR (Version 3.0.5) https://github.com/TommyJones/textmineR.

37. Wickham H and Wickham MH. Welcome to the tidyverse. Journal of Open Source Software. 2019. 4(43), 1686.

38. Wickham H and Wickham MH. ggplot2: Elegant Graphics for Data Analysis. Springer-Verlag New York . 2016. https://ggplot2.tidyverse.org/

39. Sims GE, Jun S-R, Wu GA and Kim S-H. Alignment-free genome comparison with feature frequency profiles (FFP) and optimal resolutions. Proceedings of the National Academy of Sciences. 2009;106 8:2677-82.

40. Mapleson D, Garcia Accinelli G, Kettleborough G, Wright J and Clavijo BJ. KAT: a K-mer analysis toolkit to quality control NGS datasets and genome assemblies. Bioinformatics. 2017;33 4:574-6.

41. Jain C, Koren S, Dilthey A, Phillippy AM and Aluru S. A fast adaptive algorithm for computing whole-genome homology maps. Bioinformatics. 2018;34 17:i748-i56.

42. Bernard G, Chan CX and Ragan MA. Alignment-free microbial phylogenomics under scenarios of sequence divergence, genome rearrangement and lateral genetic transfer. Scientific Reports. 2016;6 1:1-12.

43. Jacobus AP, Stephens TG, Youssef P, González-Pech R, Ciccotosto-Camp MM, Dougan KE, et al. Comparative genomics supports that Brazilian bioethanol Saccharomyces cerevisiae comprise a unified group of domesticated strains related to cachaça spirit yeasts. Frontiers in Microbiology. 2021;12:644089.
44. Bernard G, Greenfield P, Ragan MA and Chan CX. k-mer similarity, networks of microbial genomes, and taxonomic rank. Msystems. 2018;3 6:e00257-18.
45. Sims GE and Kim S-H. Whole-genome phylogeny of *Escherichia coli*/*Shigella* group by feature frequency profiles (FFPs). Proceedings of the National Academy of Sciences. 2011;108 20:8329-34.
46. Lu S, Wang J, Chitsaz F, Derbyshire MK, Geer RC, Gonzales NR, et al. CDD/SPARCLE: the conserved domain database in 2020. Nucleic Acids Research. 2020;48 D1:D265-D8.
47. Eddy SR. Profile hidden Markov models. Bioinformatics. 1998;14 9:755-63.
48. Overbeek R, Olson R, Pusch GD, Olsen GJ, Davis JJ, Disz T, et al. The SEED and the Rapid Annotation of microbial genomes using Subsystems Technology (RAST). Nucleic Acids Research. 2014;42 D1:D206-D14.
49. Arndt D, Grant JR, Marcu A, Sajed T, Pon A, Liang Y, et al. PHASTER: a better, faster version of the PHAST phage search tool. Nucleic Acids Research. 2016;44 W1:W16-W21.
50. Bouras G, Nepal R, Houtak G, Psaltis AJ, Wormald P-J and Vreugde S. Pharokka: a fast scalable bacteriophage annotation tool. Bioinformatics. 2023;39 1:btac776.

Table 1. Browsers and operating systems (OS) tested with PhageGE

OS	Chrome	Edge	Firefox	Safari
**Linux**	120.0	120.0	121.0	n/a
**MacOS**	107.0	108.0	107.0.1	15.6.1
**Windows**	105.0	108.0	107.0.1	n/a

n/a, not applicable

Table 2. Lifestyle prediction for 8 different phages

	Lytic	Temperate
**KP36**	0.993	0.007
**FK1979**	0.956	0.044
**vB8838**	0.969	0.031
**pKp20**	0.974	0.026
**NC_017985**	0	1
**NC_027339**	0.002	0.998
**NC_009815**	0.016	0.984
**NC_019768**	0.01	0.99

Figures legends:

Figure 1. The workflow and application of PhageGE.

Illustration of the workflow of PhageGE, highlighting its components and processes for phage genomic analysis. (1) **Phylogenetic analysis.** Input: Phage genome files in fasta format are uploaded; Pre-processing: The uploaded genome files are processed to estimate parameters and the are hashed for further analysis. Distance Estimation: K-mers features are normolised and then used for Jaccard index computation. Distance estimation: Distances are estimated based on the computed Jaccard index. (2) **Visualisation**. The results are visualised using the ggtree package and sample information files in CSV format. (3) **Lifestyle Prediction**. Biosequence analysis (HMMER): Biosequence analysis is performed using HMMER. Prediction model: A prediction model based on a phage genome-lifestyle dataset is applied. Lifestyle prediction: The lifestyle of the phages is predicted with the uploaded phage genome. (4) **Annotation Comparison**. Data manipulation: Genome annotation files (phaster.txt, RAST.xls, Pharokka.gff) are manipulated with built-in functions. Annotation comparison table: An annotation comparison table is generated using built-in functions.

Figure 2. Overview of PhageGE and its related functions.

The main functions and item information in PhageGE are illustrated in the figure, highlighting the steps for phylogenetic analysis, tree visualisation, lifestyle prediction, and annotation comparison. **A.** Phylogenetic Analysis: Users can select the genomes of interest by uploading phage whole genome data files (.fasta), selecting the layout of the tree (i.e., phylogram, cladogram, fan, radial and tidy), and clicking the "Explore Tree" button to initiate the phylogenetic analysis. **B.** Phylogenetic Tree Visualisation:

Users can upload a tree file (Newick or .tre format) and related genome information file (.csv). The tree visualisation displays the phylogenetic relationships among the uploaded genomes, with detailed annotations. **C.** Lifestyle Prediction: Users can select a genome of interest for lifestyle prediction by uploading a fasta file (.fasta). By clicking the "Explore Lifestyle Prediction" button, the user can predict the lifestyle of the selected genome, displaying the results with relevant statistics. **D.** Annotation Comparison: Users can upload multiple annotation files (Phaster, RAST, and Pharokka) and select the type of comparison. The resulting comparison table displays the annotated features from each source, facilitating detailed comparative analysis.

Figure 3. Comparison of phylogeny estimations from PhageGE and MSA. A. Alignment-free phylogenetic trees of 15 phages inferred from WGS data, and **B.** the topology of the reference tree inferred from multiple sequence alignment of WGS. The trees illustrate the classification and related taxa positions, demonstrating the consistency and accuracy of PhageGE's alignment-free approach in relation to the traditional MSA-based method.

Figure 4. Interactive visualisation of the phylogenetic tree of 15 phages.

Each coloured dot represents one phage, with the colour indicating the associated taxa. The pink box illustrates the additional information that can be obtained by hovering the cursor over each dot.

Figure 5. Comparison of classification accuracy of PhageGE with previously published tools across all datasets analysed.

Incorrect classification involves misidentifying the phage lifestyle (temperate or lytic).

Manuscript with mark

Click here to access/download;Manuscript;GIGA-D-24-00040R1 PhageGE_main_withMark.docx

PhageGE: An interactive web platform for exploratory analysis and visualisation of bacteriophage genomes

Jinxin Zhao[1,2*], Jiru Han[3], Yu-Wei Lin[1], Yan Zhu[1,4], Michael Aichem[5], Dimitar Garkov[5], Phillip J. Bergen[1], Sue C. Nang[1], Jian-Zhong Ye[6,7], Tieli Zhou[6,7], Tony Velkov[8], Jiangning Song[2,9], Falk Schreiber[5,10], Jian Li[1,2*]

[1] Infection Program and Department of Microbiology, Biomedicine Discovery Institute, Monash University, Clayton, VIC, Australia

[2] Monash Biomedicine Discovery Institute-Wenzhou Medical University Alliance in Clinical and Experimental Biomedicine, Monash University, Clayton, VIC, Australia

[3] Population Health and Immunity Division, The Walter and Eliza Hall Institute of Medical Research, Parkville, VIC, Australia

[4] Systems Biology Center, Tianjin Institute of Industrial Biotechnology, Chinese Academy of Sciences, Tianjin, China

[5] Department of Computer and Information Science, University of Konstanz, Konstanz, Germany

[6] Key Laboratory of Clinical Laboratory Diagnosis and Translational Research of Zhejiang Province, Department of Clinical Laboratory, The First Affiliated Hospital of Wenzhou Medical University, Zhejiang, China

[7] Wenzhou Medical University-Monash Biomedicine Discovery Institute Alliance in Clinical and Experimental Biomedicine, The First Affiliated Hospital of Wenzhou Medical University,Wenzhou, China

[8] Department of Pharmacology, Biomedicine Discovery Institute, Monash University, Melbourne, VIC, Australia

[9] Department of Biochemistry and Molecular Biology, Biomedicine Discovery Institute, Monash University, Melbourne, VIC, Australia

[10] Faculty of Information Technology, Monash University, Clayton, VIC, Australia

Email addresses: JZ, jinxin.zhao@monash.edu; JH, han.ji@wehi.edu.au; YL, yu-wei.lin@monash.edu; YZ, Yan.Zhu@monash.edu; MA, michael.aichem@uni-konstanz.de; DG, dimitar.garkov@uni-konstanz.de; PB, phillip.bergen@monash.edu; SN, sue.nang@monash.edu; JY, jzye89@163.com; TZ, wyztli@163.com; TV, tony.velkov@monash.edu; JS, jiangning.song@monash.edu; FS, falk.schreiber@uni-konstanz.de; JL, jian.li@monash.edu.

Running title: PhageGE for bacteriophage genomic analysis

*Corresponding authors:
Dr Jinxin Zhao, Tel: +61 3 99056288, Email: jinxin.zhao@monash.edu;
Professor Jian Li, Tel: +61 3 99039172 Fax: +61 0399056450; Email: jian.li@monash.edu.

Abstract

Background: Antimicrobial resistance is a serious threat to global health. Due to the stagnant antibiotic discovery pipeline, bacteriophages (phages) have been proposed as an alternative therapy for the treatment of infections caused by multidrug-resistant (MDR) pathogens. Genomic features play an important role in phage pharmacology. However, our knowledge of phage genomics is sparse and the use of existing bioinformatic pipelines and tools requires considerable bioinformatic expertise. These challenges have substantially limited the clinical translation of phage therapy.

Findings: We have developed PhageGE (Phage Genome Explorer), a user-friendly graphical interface application for the interactive analysis of phage genomes. PhageGE enables users to perform key analyses, including phylogenetic analysis, visualisation of phylogenetic trees, prediction of phage lifecycle, and comparative analysis of phage genome annotations. The new R Shiny webserver, PhageGE, integrates existing R packages and combines them with several newly developed functions to facilitate these analyses. Additionally, the webserver provides interactive visualisation capabilities and allows users to directly export publication-quality images.

Conclusions: PhageGE is a valuable tool that simplifies the analysis of phage genome data and may expedite the development and clinical translation of phage therapy. PhageGE is publicly available at http://www.phagege.com/.

Keywords: Phage genome, biological web application, genomic analysis, phylogeny, lifestyle

Introduction

The rapid emergence and spread of antimicrobial resistance (AMR) is one of the three greatest threats to human health globally [1]. It is estimated that by 2050, life-threatening infections caused by antimicrobial-resistant pathogens will kill more people than any other diseases [2]. Of particular concern is the increased prevalence of infections caused by Gram-negative pathogens, which are more difficult to treat than Gram-positive pathogens [3]. Given the sluggish global antibiotic pipeline [4], bacteriophages (phages) have attracted significant attention over the last decade as a potential alternative therapy for bacterial infections [5]. Phages are bacterial viruses and the advantages of phage therapy over antibiotics include a narrow spectrum of activity, the capacity to multiply at the infection site, and safety [6-8]. Optimising phage therapy in patients requires key pharmacological information, including infection cycle, gene content, and phage taxonomy [9, 10]. For example, temperate phages do not immediately lyse bacterial host cells and have an inherent capacity to mediate the transfer of genes between bacteria, potentially facilitating increased bacterial virulence and AMR. In contrast, lytic phages kill bacteria upon infection and are commonly used for the treatment of MDR bacterial infections in patients [11-14].

Multi-omics has the potential to expedite the clinical translation of phage therapy for the treatment of MDR bacterial infections [15]. For example, whole genome-based phylogenetic analysis offers significant advantages in understanding phage evolutionary dynamics and designing potential phage cocktails [16, 17]. Furthermore, combining whole-genome sequencing (WGS) with *in silico* prediction enables rapid prediction of phage lifestyle [18]. Several popular bioinformatic pipelines and tools are available for multiple sequence alignment (MAFFT) [19], phylogenetic reconstruction (RAxML and IQ-TREE) [20, 21], visualisation of phylogeny (ggtree) [22], and phage

lifestyle prediction (PHACTS and BACPHLIP) [18, 23]; however, utilising these tools requires proficient programming skills. Therefore, a user-friendly platform for phage genomic analyses is urgently needed to overcome the challenges associated with the requirement for advanced programming expertise.

Here, we developed an integrated webserver platform, PhageGE, that offers four key functionalities: phage phylogenetic analysis, tree visualisation, lifestyle prediction, and manipulation of phage genome annotation datasets. PhageGE differs from existing phage genomic analysis tools in that it facilitates the seamless export of all associated results in a publication-ready format without requiring complex procedures or long running times. Overall, PhageGE provides a user-friendly interface to streamline phage genomic analysis with WGS data.

Results

The PhageGE webserver (biotoolsID: biotools:phagege and RRID: SCR_025380) was designed to ensure user-friendliness and compatibility with major web browsers, including Google Chrome, Mozilla Firefox, Apple Safari, and Microsoft Edge (**Table 1**).

Webserver submission and case studies

To demonstrate the functions and the scope of application of PhageGE, we herein describe the results of a case study using PhageGE, including phage whole genome data (i.e., .fasta), a phylogenetic tree file (i.e., .tre), and genome annotation data (i.e., .xls, .txt and .gff), which are collectively referred to as "Example Data" (**Figure 1**). The complete set of Example Data used in the case studies can be accessed on the PhageGE GitHub repository (https://github.com/JinxinMonash/PhageGE).

Phage phylogenetic analysis and visualisation

To illustrate the phylogenetic analysis function in PhageGE and its application in clinical translation, we analysed our GitHub example dataset, which consists of 15 phage genomes. The hosts of the 15 phage genomes in the phylogenetic analysis are from 3 different bacterial species: *Citrobacter freundii, Escherichia coli, and Klebsiella pneumoniae* (**Figure 2A**). This dataset includes one anti-Klebsiella phage, pKp20, which was isolated in our lab and used in a clinical case [24]. In that case, a recurrent urinary tract infection [rUTI] was successfully treated with 4 weeks of adjunctive intravenous bacteriophage therapy, with no recurrence during a year of follow-up [24]. Both taxonomy information from phylogeny analysis and the lifestyle prediction played key roles in the selection of pKp20 over a wide range of phages [24]. The phage WGS data in the fasta format can be obtained either from NCBI or prepared locally using standard genome assembly pipelines (e.g., SPAdes) based on the previous BLASTn result [24]. To compare the results obtained from PhageGE with the multiple sequence alignment-based approach, we also conducted a multiple sequence alignment-based phylogenetic analysis using MAFFT v7.47 and fasttree v2.1.10, alongside the phylogenetic analysis using PhageGE. We firstly uploaded the selected fasta files or a multi-fasta file which contains all phage genomes on the Phylogenetic Analysis page in PhageGE, then selected the layout of the tree (i.e., phylogram, cladogram, fan, radial, or tidy) and clicked the “Explore Tree” icon. The resulting phylogenetic tree, representing the relationships among the uploaded genomes, was generated using the built-in *k*-mer-based alignment-free phylogenetic approach, as detailed in the Methods section (**Figures 2A and 3A**). To enhance the clarity, we manually highlighted the 15 phages with distinct colours according to their genus. Comparison of the phylogenetic trees generated by PhageGE and MAFFT revealed that both trees

shared largely the same classification (e.g., positions of each phage and the related taxa) (**Figure 3**). Moreover, PhageGE demonstrates a significant improvement in runtime efficiency. For example, on a 2-GHz CPU with 64 GB RAM server, the runtimes of generating phylogenetics trees by PhageGE were 0.22 minutes for 15 phage genomes and 4.42 minutes for 146 phage genomes. In contrast, the MSA-based approach (using tools like MAFFT along with FastTree) took 30 minutes and 296 minutes, respectively. This demonstrates that the performance of the phylogenetic analysis of PhageGE is accurate, fast and comparable to the multiple sequence alignment-based approach.

The phylogenetic visualisation function handles the phylogenetic tree along with diverse accompanying data. Its aim is to provide an interactive visualisation platform that enhances the accessibility of phylogenetic data and facilitates the phylogenetic analysis of phage comparative genomics studies. The phylogenetic tree and associated data can be extracted using a built-in function within PhageGE. This function is illustrated using a tree file “phage.tre” obtained from phage phylogenetic analysis (whether generated by PhageGE or other phylogenetic analysis pipeline) and a sample information file named “sample_info.csv” containing the taxonomy information for all 14 phages (**Figure 2B**). As shown in **Figure 4**, each dot in the dendrogram represents one phage with the colour indicating its taxonomic classification in the same genus. In addition, detailed information of each phage (e.g., name and taxonomy) can be easily accessed by hovering the cursor over the dot of interest (as indicated by the pink box in **Figure 4**). This interactive feature allows users to dynamically integrate and visualise the underlying information in a user-friendly manner.

Performance of phage lifestyle prediction

The lifestyle prediction function builds on a Random Forest classifier that incorporates up-to-date conserved protein domains with the ability to classify temperate and lytic phages using WGS data. To evaluate its performance, we compared the function with other published tools using the dataset of 1,057 phages in the literature [25]. The PhageGE lifestyle prediction function achieved the lowest error rates (0%, 1.2%, 0.3% and 2.5%, equivalent to 100%, 98.8%, 99.7% and 97.5% classification accuracy, respectively) across all tested datasets, substantially outperforming those existing tools for phage lifestyle classification (**Figure 5**). The prediction accuracy of PhageGE exceeded that of the most accurate existing tool, BACPHLIP, which had prediction accuracies of 99.8%, 98.3%, 99.2% and 96.5%, respectively (**Figure 5**). Similarly, WGS data for individual phages (e.g., *Klebsiella* phage KP36.fasta, vB8388.fasta, and FK1979.fasta from the example dataset described here) can be uploaded as input to generate the phage lifestyle probability table (**Figure 2C** and **Table 2**). The result presented in **Table 2** predicts that *Klebsiella* phages KP36 (a model phage in our laboratory), FK1979, and vB8388 [26] (two phages isolated from hospital sewage, The First Affiliated Hospital of Wenzhou Medical University, China), and pKp20 (used in the rUTI clinical case study) [24], are highly likely lytic phages, with the probability of 99.3%, 95.6% and 96.9%, respectively. Meanwhile, the four phages from the NCBI in **Table 2** NC_017985, NC_027339, NC_009815, and NC_019768 are highly likely temperate phages. This function empowers users to rapidly analyse the lifestyle of a phage of interest *in silico* with high prediction accuracy, providing key insights into the intricate phage ecosystems and enabling optimal design of phage therapy.

Comparison of phage genome annotation

Notably, PhageGE also provides a function to compare phage genome annotations obtained from different pipelines (i.e., Pharokka, Phaster and RAST). This analysis

involves the integration of R package flextable, which allows for the generation of downloadable comparison results in multiple formats (e.g., csv, Excel and PDF). The user interface offers the flexibility to rank the results based on multiple parameters (e.g., location and/or length of the coding sequence [CDS]). In the case study presented here, we used PhageGE to compare genome annotations of *Klebsiella* phages KP36, vB8838, and FK1979 generated from Phaster, RAST, and Pharokka (**Figure 2D**). By selecting "common_annotation", a table with 75, 45, and 51 genes that were annotated in all three pipelines was generated for KP36, vB8838, and FK1979, respectively. We also identified 17, 7, and 12 unique genes from the Pharokka pipeline by selecting the "Pharokka_only" option. To gain a better understanding of those unique annotated genes, PhageGE allows users to directly copy and download both the nucleotide and amino acid sequences associated with the genes from the interactive table. This feature facilitates further investigation of these unique annotations.

Discussion

With the dramatic rise in MDR bacterial infections, phage therapy has emerged as a safe and potentially effective alternative treatment option to antibiotics [27]. However, the development of effective phage therapies is complex, involving the isolation, culturing, characterisation, and timely preparation of efficacious phages. Traditionally, this process is time-consuming and costly [28, 29]. Nevertheless, with the next-generation sequencing techniques, it has become possible to rapidly and cost-effectively characterise phages. Despite this advancement, there is a paucity of intuitive tools available for phage genomics, with the majority requiring operation in

command-line mode. The availability of large phage genomic datasets presents unique opportunities to develop bioinformatics tools that aid in phage biology and pharmacology research. The use of computational methods to study phages has shown promise in generating novel insights, such as phylogeny and lifestyle, through bioinformatic analysis [18, 25, 30]. However, there is currently no single tool available that encompasses all those functions (e.g., phylogenetic analysis, tree visualisation, lifestyle prediction, and genome annotation comparison) in the webserver platform. Herein, we describe the development of the PhageGE webserver GUI streamlined for user-friendly phage genomic analysis.

PhageGE is a novel, user-friendly GUI application for the interactive analysis of phage genomes. The overarching goal of PhageGE is to provide an interactive analysis and visualisation platform for the rapid exploration of phage genomic associations, thereby promoting efficient genomic data-driven discovery of phage therapy. PhageGE comprises a set of functions for phage genomic analysis, including phylogenetic analysis, tree visualisation, lifestyle prediction, and genome annotation comparison. While current tools like PhaGAA can provide lifestyle reorganisation analysis, their primary utility lies in analysing phage lifestyle for their prefered phage dataset (e.g., gut flora of human neonates) [31]. In contrast, PhageGE integrates a more comprehensive dataset with a wide range of phage genomes, allowing for broader and deeper exploration of phage lifestyles. Moreover, the comparsion of annotations from different pipelines highlights the key role of PhageGE in advancing phage genomics through enhanced analysis and visualisation functions. To exemplify the utility of PhageGE, we investigated the phylogeny, lifestyle, and annotation comparison of *Klebsiella* phages KP36, vB8838, and FK1979, which were independently isolated in two different countries. Our findings demonstrate that the various functions of

PhageGE yield comparable or better results than existing state-of-the-art approaches. These results highlight the significant potential of PhageGE in analysing various phage genomic features using phage WGS data.

Notably, PhageGE requires only phage WGS data as the input for conducting the related analysis. The phage phylogenetic analysis function takes phage WGS in the fasta format as input and applies an alignment-free phylogenetic approach to infer phylogenetic relationships. Compared to current phylogenetic analysis pipelines (i.e., multiple sequence alignment-based phylogenetic analysis), analysis from PhageGE showed similar phage phylogeny information in a shorter computing time (approximately 13 seconds versus 30 minutes for 15 phage genomes). Moreover, the result from phylogenetic analysis can be easily exported in various graphical formats (e.g., SVG, PDF and JPEG) and textual formats (e.g., Newick and Nexus) and can be interactively managed and viewed through our designed user interface. In addition, PhageGE introduces an enhanced phage lifestyle prediction function, using a machine-learning approach with updated databases for conserved protein domains. The overall approaches applied for both phylogenetic analysis and lifestyle prediction demonstrate that analyses results from PhageGE are comparable to previously published tools (**Figures 3** and **5**), showing its effectiveness in accurately analysing phage phylogeny and predicting phage lifestyle**.** Notably, PhageGE incorporates a function of annotation comparison to facilitate the efficient organisation of genome annotation files derived from different annotation pipelines. This feature allows users to efficiently compare genome annotation data obtained with different tools. Overall, all fours functions from PhageGE serve as a guide for the exploration of phage genomic features and will expedite the clinical translation of phage therapy.

Conclusion

In conclusion, PhageGE is the first user-friendly tool for the analysis of phage genomes, offering improved functions compared to existing tools without the need for considerable programming skills. Uniquely incorporating features like phylogenetic analysis, interactive tree visualisation, lifestyle prediction, and genome annotation comparison, we anticipate that PhageGE will become an instrumental bioinformatic web server for phage genomic analysis, guiding experimental validations and advancing the development of phage therapy.

Methods

Implementation

PhageGE 1.0 was developed in R and is hosted on Shinyapps. This application seamlessly integrates various R packages, including Rshiny, seqinr, Biostrings, ape, textmineR, tidyverse, ggtree, ploty, ggplot, reticulate, and pyhmmer [22, 32-38]. Furthermore, it incorporates several key functions, including *k*-mer-based phylogeny estimation, phylogenetic tree visualisation, lifestyle prediction, and annotation comparison. To use PhageGE, input files in the standard WGS fasta format are required, along with textual tables in standard formats (e.g., csv or xlsx) containing sequence details and annotation information. The workflow is illustrated in **Figure 1**.

Phage genomic analysis pipeline

The functionalities offered in the web interface of PhageGE utilise WGS fasta files for phylogenetic analysis and lifestyle prediction. Users can input tree files (e.g., Newick or Nexus) and textual files (i.e., csv or xlsx) for phylogenetic tree visualisation and

genome annotation comparisons. Using these standard formats as input files facilitates effective use and simplifies data export for users.

Phylogenetic analysis and phylogenetic tree visualisation

The phylogenetic analysis function enables fast and efficient analysis of phage phylogeny. It includes phylogeny reconstruction based on the input WGS data and visualisation of phylogenetic information. This function incorporates a *k*-mer-based alignment-free phylogenetic approach [39]. Alignment-free phylogenetic approaches offer a scalable alternative for inferring phylogenetic relationships and computing local alignment boundaries from WGS data [40, 41]. This approach is particularly robust for genome sequences that exhibit genetic recombinations and rearrangements. It has demonstrated the ability to accurately reconstruct biologically relevant phylogenies with thousands of microbial genomes [42-44]. The description of this function is briefly outlined below.

Consider a sequence consisting of four characters (A, T, C, G) of length *k* (‘*k*-mer’), described by **Equation 1**. There are 4^k possible *k*-mers (**Equation 2**), which can serve as features of each genome. The value assigned to a specific *k*-mer feature will correspond to the number of occurrences of that *k*-mer in the genome. Using these *k*-mer features, a data matrix is generated with dimensions of the numbers of genomes of interest (*n* columns) by 4^k rows. To establish a representative probability distribution of the 4^k *k*-mers, each row of the data matrix is normslised by its row total. This normalisation results in a feature-frequency profile (F_k, described by **Equation 3**) for each *k*-mers sequence [39]. The Jensen-Shannon divergence (D_k, described by **Equation 4**) is then employed to estimate the genome pairwise distances [45]. Subsequently, the resulting distance matrix is used as an input for a clustering

algorithm (e.g., neighbor-joining algorithm) to summarise the relatedness of the phage genomes and construct a phylogenetic tree [35].

Equation 1: $C_k = < C_{k,1}, C_{k,2} \cdots C_{k,m} >$

Equation 2: $m = 4^k$

Equation 3: $F_{n_i,k} = \frac{c_{n_i,k_m}}{\sum_{n_i} c_{n_i,k}}$

Equation 4: $D_k = JS(F_{n_1,k}, F_{n_i,k})$

An interactive visualisation of a phylogenetic tree was generated either from the phylogenetic analysis function or a customised phylogenetic tree that includes additional information, such as species classification, duplication events, and bootstrap values. It is implemented using ggtree and ploty R packages [22], ensuring the ability to handle most common tree formats (e.g., Newick, Nexus, and tre).

Lifestyle prediction

The Lifestyle Prediction function in PhageGE generates a phage lifestyle probability table based on the input of phage WGS data. This function adapted previously reported approaches into our user-friendly interface [18, 23, 25]. By employing an improved search function (i.e. searching a sequence file against the build-in Hidden Markov Model [HMM] database), PhageGE provides an efficient way to predict phage lifestyle based on the phage genomic information.

In brief, we first conducted a search in the Conserved Domain Database (accessed: 11/2023) to collect protein domains from temperate phages [46]. The following key

words were used to identify relevant protein domains: ‘temperate’, ‘lysogen’, ‘integrase’, ‘excisionase’, ‘recombinase’, ‘transposase’, ‘parA|parB’, and ‘xerC|xerD’. We obtained a total of 477 protein domains from the initial collection, which were then subjected to a careful manual curation and filtration (e.g., minimal domain length >30 and validated in the existing experimental data), resulting in a refined set of 261 protein domains. Next, a lifestyle classification model was trained and tested using a published dataset consisting of 1,057 phages from 6 different families (*Inoviridae*, *Myoviridae*, *Plasmaviridae*, *Podoviridae*, *Siphoviridae*, and *Tectiviridae*) across 55 host genera, with known genome and lifestyle information [25]. The dataset was randomly split into training and testing sets, with a ratio of 60:40 (634 phages in the training set and 423 phages in the testing set). At this stage, the testing set was fully set aside for subsequent descriptions related to model training and development. For each genome sequence in the training set, we generated a list of all possible 6-frame translation sequences that were at least 40 amino acids long. HMMER3 was then used to search for the presence or absence of the various protein domains listed above, resulting in a vector for each phage describing the presence (1) or absence (0) of each domain [47]. This information allowed us to filter the initial set of 477 putatively useful protein domains down to the final set of 261. Subsequently, a Random Forest classifier was fitted to the training set of phage genomes, and cross-validation was employed to fine-tune the model hyper-parameters. The ‘best’ performing model was then selected by choosing the hyper-parameters that yielded the highest minimum accuracy across the independent validation set tests. The parameters of that model were then re-fitted to the entire training set data, resulting in the final model.

Annotation comparison

The Rapid Annotation using Subsystem Technology (RAST) server was developed in 2008 to annotate microbial genomes based on the manually curated SEED database [48]. The PHAge Search Tool – Enhanced Release (PHASTER) was specifically designed to identify and annotate prophage sequences within bacteria using prophage/virus databases [49]. More recently, another phage annotation tool, Pharokka, has been developed using PHROGS, CARD, and VFDB databases [50]. Since these pipelines employ different databases for phage genome annotation, it is possible to obtain different annotations from each pipeline. To provide more comprehensive annotation results, there is an urgent need for annotation comparison tables that incorporate all annotation information from RAST, PHASTER, and Pharokka. The Annotation Comparison function in PhageGE generates interactive tables that display comments and differing genome annotation information obtained from RAST, PHASTER, and Pharokka. This comparison includes checking the coding regions and related annotations from each pipeline. Moreover, it provides an overview of common and different annotation counts, facilitating the tracking of differences between the three pipelines. This function is implemented using the flextable, tidyselect, data.table, and tidyverse packages [37].

Code availability and requirements

- Project name: PhageGE (Phage Genome Exploration)
- Project homepage: https://github.com/JinxinMonash/PhageGE
- Operating system(s): Linux, Windows and MacOS (**Table 1**)
- Programming language: R
- License: MIT license

Data availability

In general, all data used in this work were from openly accessible public repositories and released with other publications under open-source licenses. The data used were solely for research purposes, and we confirm that they were not used for any other noncommercial or commercial purpose. The datasets supporting the results of this article are available in the Github repository, [https://github.com/JinxinMonash/PhageGE]. The data used as examples can be found in the release branch called “Example data” or “Example data.zip” within our repository.The GitHub repository also contains up-to-date tutorials.

Competing interests

The authors declare that they have no competing interests.

Funding

This work was supported by the National Institute of Allergy and Infectious Diseases of the National Institutes of Health [grant number R21 AI156766 to J.L.]. The content is solely the responsibility of the authors and does not necessarily represent the official views of the National Institute of Allergy and Infectious Diseases or the National Institutes of Health.

Author’s contributions

J.Z. collected all the data and participated in developing the webserver and writing the manuscript. J.H., Y.W.L., Y.Z., M.A. and D.G. and J.N.S. contributed to the

development of the web server. P.J.B., S.N., J.Z.Y., T.L.Z. and T.V. took part in the discussion of the data. J.Z., F.S. and J.L. conceived the study, coordinated the work and contributed to writing the manuscript. All authors are involved in the discussion and finalisation of the manuscript.

Acknowledgements

J.Z. is a recipient of the 2022 Faculty of Medicine, Nursing and Health Sciences Bridging Fellowship, Monash University. J.L. is an Australian National Health Medical Research Council (NHMRC) Investigator Research Fellow and T.V. is an Australian Research Council (ARC) Industrial Fellow. Y.W.L. is currently an employee of Certara, Australia and Co-Director of the Malaya Translational and Clinical Pharmacometrics Group, University of Malaya, Malaysia.

References

1. Murray CJ, Ikuta KS, Sharara F, Swetschinski L, Aguilar GR, Gray A, et al. Global burden of bacterial antimicrobial resistance in 2019: a systematic analysis. The Lancet. 2022;399 10325:629-55.
2. Luepke KH, Suda KJ, Boucher H, Russo RL, Bonney MW, Hunt TD, et al. Past, present, and future of antibacterial economics: increasing bacterial resistance, limited antibiotic pipeline, and societal implications. Pharmacotherapy: The Journal of Human Pharmacology and Drug Therapy. 2017;37 1:71-84.
3. Bassetti M and Garau J. Current and future perspectives in the treatment of multidrug-resistant Gram-negative infections. Journal of Antimicrobial Chemotherapy. 2021;76 (Suppl 4):iv23-iv37.
4. Böttcher L, Gersbach H and Wernli D. Restoring the antibiotic R&D market to combat the resistance crisis. Science and Public Policy. 2022;49 1:127-31.
5. Uyttebroek S, Chen B, Onsea J, Ruythooren F, Debaveye Y, Devolder D, et al. Safety and efficacy of phage therapy in difficult-to-treat infections: a systematic review. The Lancet Infectious Diseases. 2022; 22 8:E208-E220.
6. Kortright KE, Chan BK, Koff JL and Turner PE. Phage therapy: a renewed approach to combat antibiotic-resistant bacteria. Cell Host & Microbe. 2019;25 2:219-32.
7. Mousavi SM, Babakhani S, Moradi L, Karami S, Shahbandeh M, Mirshekar M, et al. Bacteriophage as a novel therapeutic weapon for killing colistin-resistant multi-drug-resistant and extensively drug-resistant gram-negative bacteria. Current Microbiology. 2021;78 12:4023-36.
8. Lin Y, Chang RY, Rao G, Jermain B, Han M-L, Zhao J, et al. Pharmacokinetics/pharmacodynamics of antipseudomonal bacteriophage

therapy in rats: a proof-of-concept study. Clinical Microbiology and Infection. 2020;26 9:1229-35.

9. Hyman P. Phages for phage therapy: isolation, characterization, and host range breadth. Pharmaceuticals. 2019;12 1:35.

10. Gordillo Altamirano FL and Barr JJ. Phage therapy in the postantibiotic era. Clinical Microbiology Reviews. 2019;32 2:e00066-18.

11. Roach DR, Leung CY, Henry M, Morello E, Singh D, Di Santo JP, et al. Synergy between the host immune system and bacteriophage is essential for successful phage therapy against an acute respiratory pathogen. Cell Host & Microbe. 2017;22 1:38-47. e4.

12. Harrison E and Brockhurst MA. Ecological and evolutionary benefits of temperate phage: what does or doesn't kill you makes you stronger. BioEssays. 2017;39 12:1700112.

13. Gill JJ and Hyman P. Phage choice, isolation, and preparation for phage therapy. Current Pharmaceutical Biotechnology. 2010;11 1:2-14.

14. Abedon ST, García P, Mullany P and Aminov R. Phage therapy: past, present and future. Frontiers Media SA, 2017, p. 981.

15. Debnath M, Prasad GB and Bisen PS. Omics technology. Molecular Diagnostics: Promises and Possibilities. Springer; 2010. p. 11-31.

16. Parmar KM, Dafale NA, Tikariha H and Purohit HJ. Genomic characterization of key bacteriophages to formulate the potential biocontrol agent to combat enteric pathogenic bacteria. Archives of Microbiology. 2018;200 4:611-22.

17. Philipson CW, Voegtly LJ, Lueder MR, Long KA, Rice GK, Frey KG, et al. Characterizing phage genomes for therapeutic applications. Viruses. 2018;10 4:188.

18. McNair K, Bailey BA and Edwards RA. PHACTS, a computational approach to classifying the lifestyle of phages. Bioinformatics. 2012;28 5:614-8.
19. Katoh K and Standley DM. MAFFT multiple sequence alignment software version 7: improvements in performance and usability. Molecular Biology and Evolution. 2013;30 4:772-80.
20. Stamatakis A. RAxML version 8: a tool for phylogenetic analysis and post-analysis of large phylogenies. Bioinformatics. 2014;30 9:1312-3.
21. Minh BQ, Schmidt HA, Chernomor O, Schrempf D, Woodhams MD, Von Haeseler A, et al. IQ-TREE 2: new models and efficient methods for phylogenetic inference in the genomic era. Molecular Biology and Evolution. 2020;37 5:1530-4.
22. Xu S, Li L, Luo X, Chen M, Tang W, Zhan L, et al. Ggtree: A serialized data object for visualization of a phylogenetic tree and annotation data. iMeta. 2022;3 1:e56.
23. Hockenberry AJ and Wilke CO. BACPHLIP: predicting bacteriophage lifestyle from conserved protein domains. PeerJ. 2021;9:e11396.
24. Le T, Nang SC, Zhao J, Yu HH, Li J, Gill JJ, et al. Therapeutic potential of intravenous phage as standalone therapy for recurrent drug-resistant urinary tract infections. Antimicrobial Agents and Chemotherapy. 2023;67 4:e00037-23.
25. Mavrich TN and Hatfull GF. Bacteriophage evolution differs by host, lifestyle and genome. Nature Microbiology. 2017;2 9:1-9.
26. Zhao Y, Feng L, Zhou B, Zhang X, Yao Z, Wang L, et al. A newly isolated bacteriophage vB8388 and its synergistic effect with aminoglycosides against

multi-drug resistant *Klebsiella oxytoca* strain FK-8388. Microbial Pathogenesis. 2023;174:105906.

27. Khan A, Rao TS and Joshi HM. Phage therapy in the Covid-19 era: Advantages over antibiotics. Current Research in Microbial Sciences. 2022;3:100115.

28. Camarillo-Guerrero LF, Almeida A, Rangel-Pineros G, Finn RD and Lawley TD. Massive expansion of human gut bacteriophage diversity. Cell. 2021;184 4:1098-109.

29. Carrigy NB, Larsen SE, Reese V, Pecor T, Harrison M, Kuehl PJ, et al. Prophylaxis of *Mycobacterium tuberculosis* H37Rv infection in a preclinical mouse model via inhalation of nebulized bacteriophage D29. Antimicrobial Agents and Chemotherapy. 2019;63 12:e00871-19.

30. Martinez-Vaz BM and Mickelson MM. In silico phage hunting: bioinformatics exercises to identify and explore bacteriophage genomes. Frontiers in Microbiology. 2020;11:577634.

31. Wu J, Liu Q, Li M, Xu J, Wang C, Zhang J, et al. PhaGAA: an integrated web server platform for phage genome annotation and analysis. Bioinformatics. 2023;39 3:btad120.

32. Sievert C. Interactive web-based data visualization with R, plotly, and shiny. Journal of the Royal Statistical Society Series A: Statistics in Society. 2020;184:1150.

33. Charif D and Lobry JR. SeqinR 1.0-2: a contributed package to the R project for statistical computing devoted to biological sequences retrieval and analysis. Structural approaches to sequence evolution. Springer; 2007. p. 207-32.

34. Pagès H, Aboyoun P, Gentleman R, DebRoy S. Biostrings: Efficient manipulation of biological strings. 2024. Biostrings (Version 2.70.2) https://bioconductor.org/packages/Biostrings.

35. Paradis E and Schliep K. ape 5.0: an environment for modern phylogenetics and evolutionary analyses in R. Bioinformatics. 2019;35 3:526-8.

36. Jones T, Doane W and Jones MT. Package ‘textmineR’. Functions for text mining and topic modeling. 2021. textmineR (Version 3.0.5) https://github.com/TommyJones/textmineR.

37. Wickham H and Wickham MH. Welcome to the tidyverse. Journal of Open Source Software. 2019. 4(43), 1686.

38. Wickham H and Wickham MH. ggplot2: Elegant Graphics for Data Analysis. Springer-Verlag New York . 2016. https://ggplot2.tidyverse.org/

39. Sims GE, Jun S-R, Wu GA and Kim S-H. Alignment-free genome comparison with feature frequency profiles (FFP) and optimal resolutions. Proceedings of the National Academy of Sciences. 2009;106 8:2677-82.

40. Mapleson D, Garcia Accinelli G, Kettleborough G, Wright J and Clavijo BJ. KAT: a K-mer analysis toolkit to quality control NGS datasets and genome assemblies. Bioinformatics. 2017;33 4:574-6.

41. Jain C, Koren S, Dilthey A, Phillippy AM and Aluru S. A fast adaptive algorithm for computing whole-genome homology maps. Bioinformatics. 2018;34 17:i748-i56.

42. Bernard G, Chan CX and Ragan MA. Alignment-free microbial phylogenomics under scenarios of sequence divergence, genome rearrangement and lateral genetic transfer. Scientific Reports. 2016;6 1:1-12.

43. Jacobus AP, Stephens TG, Youssef P, González-Pech R, Ciccotosto-Camp MM, Dougan KE, et al. Comparative genomics supports that Brazilian bioethanol Saccharomyces cerevisiae comprise a unified group of domesticated strains related to cachaça spirit yeasts. Frontiers in Microbiology. 2021;12:644089.
44. Bernard G, Greenfield P, Ragan MA and Chan CX. k-mer similarity, networks of microbial genomes, and taxonomic rank. Msystems. 2018;3 6:e00257-18.
45. Sims GE and Kim S-H. Whole-genome phylogeny of *Escherichia coli*/*Shigella* group by feature frequency profiles (FFPs). Proceedings of the National Academy of Sciences. 2011;108 20:8329-34.
46. Lu S, Wang J, Chitsaz F, Derbyshire MK, Geer RC, Gonzales NR, et al. CDD/SPARCLE: the conserved domain database in 2020. Nucleic Acids Research. 2020;48 D1:D265-D8.
47. Eddy SR. Profile hidden Markov models. Bioinformatics. 1998;14 9:755-63.
48. Overbeek R, Olson R, Pusch GD, Olsen GJ, Davis JJ, Disz T, et al. The SEED and the Rapid Annotation of microbial genomes using Subsystems Technology (RAST). Nucleic Acids Research. 2014;42 D1:D206-D14.
49. Arndt D, Grant JR, Marcu A, Sajed T, Pon A, Liang Y, et al. PHASTER: a better, faster version of the PHAST phage search tool. Nucleic Acids Research. 2016;44 W1:W16-W21.
50. Bouras G, Nepal R, Houtak G, Psaltis AJ, Wormald P-J and Vreugde S. Pharokka: a fast scalable bacteriophage annotation tool. Bioinformatics. 2023;39 1:btac776.

Table 1. Browsers and operating systems (OS) tested with PhageGE

OS	Chrome	Edge	Firefox	Safari
**Linux**	120.0	120.0	121.0	n/a
**MacOS**	107.0	108.0	107.0.1	15.6.1
**Windows**	105.0	108.0	107.0.1	n/a

n/a, not applicable

Table 2. Lifestyle prediction for 8 different phages

	Lytic	Temperate
**KP36**	0.993	0.007
**FK1979**	0.956	0.044
**vB8838**	0.969	0.031
**pKp20**	0.974	0.026
**NC_017985**	0	1
**NC_027339**	0.002	0.998
**NC_009815**	0.016	0.984
**NC_019768**	0.01	0.99

Figures legends:

Figure 1. The workflow and application of PhageGE.

Illustration of the workflow of PhageGE, highlighting its components and processes for phage genomic analysis. (1) **Phylogenetic analysis.** Input: Phage genome files in fasta format are uploaded; Pre-processing: The uploaded genome files are processed to estimate parameters and the are hashed for further analysis. Distance Estimation: K-mers features are normolised and then used for Jaccard index computation. Distance estimation: Distances are estimated based on the computed Jaccard index. (2) **Visualisation**. The results are visualised using the ggtree package and sample information files in CSV format. (3) **Lifestyle Prediction**. Biosequence analysis (HMMER): Biosequence analysis is performed using HMMER. Prediction model: A prediction model based on a phage genome-lifestyle dataset is applied. Lifestyle prediction: The lifestyle of the phages is predicted with the uploaded phage genome. (4) **Annotation Comparison**. Data manipulation: Genome annotation files (phaster.txt, RAST.xls, Pharokka.gff) are manipulated with built-in functions. Annotation comparison table: An annotation comparison table is generated using built-in functions.

Figure 2. Overview of PhageGE and its related functions.

The main functions and item information in PhageGE are illustrated in the figure, highlighting the steps for phylogenetic analysis, tree visualisation, lifestyle prediction, and annotation comparison. **A.** Phylogenetic Analysis: Users can select the genomes of interest by uploading phage whole genome data files (.fasta), selecting the layout of the tree (i.e., phylogram, cladogram, fan, radial and tidy), and clicking the "Explore Tree" button to initiate the phylogenetic analysis. **B.** Phylogenetic Tree Visualisation:

Users can upload a tree file (Newick or .tre format) and related genome information file (.csv). The tree visualisation displays the phylogenetic relationships among the uploaded genomes, with detailed annotations. **C.** Lifestyle Prediction: Users can select a genome of interest for lifestyle prediction by uploading a fasta file (.fasta). By clicking the "Explore Lifestyle Prediction" button, the user can predict the lifestyle of the selected genome, displaying the results with relevant statistics. **D.** Annotation Comparison: Users can upload multiple annotation files (Phaster, RAST, and Pharokka) and select the type of comparison. The resulting comparison table displays the annotated features from each source, facilitating detailed comparative analysis.

Figure 3. Comparison of phylogeny estimations from PhageGE and MSA. A. Alignment-free phylogenetic trees of 15 phages inferred from WGS data, and **B.** the topology of the reference tree inferred from multiple sequence alignment of WGS. The trees illustrate the classification and related taxa positions, demonstrating the consistency and accuracy of PhageGE's alignment-free approach in relation to the traditional MSA-based method.

Figure 4. Interactive visualisation of the phylogenetic tree of 15 phages.

Each coloured dot represents one phage, with the colour indicating the associated taxa. The pink box illustrates the additional information that can be obtained by hovering the cursor over each dot.

Figure 5. Comparison of classification accuracy of PhageGE with previously published tools across all datasets analysed.

Incorrect classification involves misidentifying the phage lifestyle (temperate or lytic).

Figure 1

Click here to access/download;Figure;Figure 1.jpg

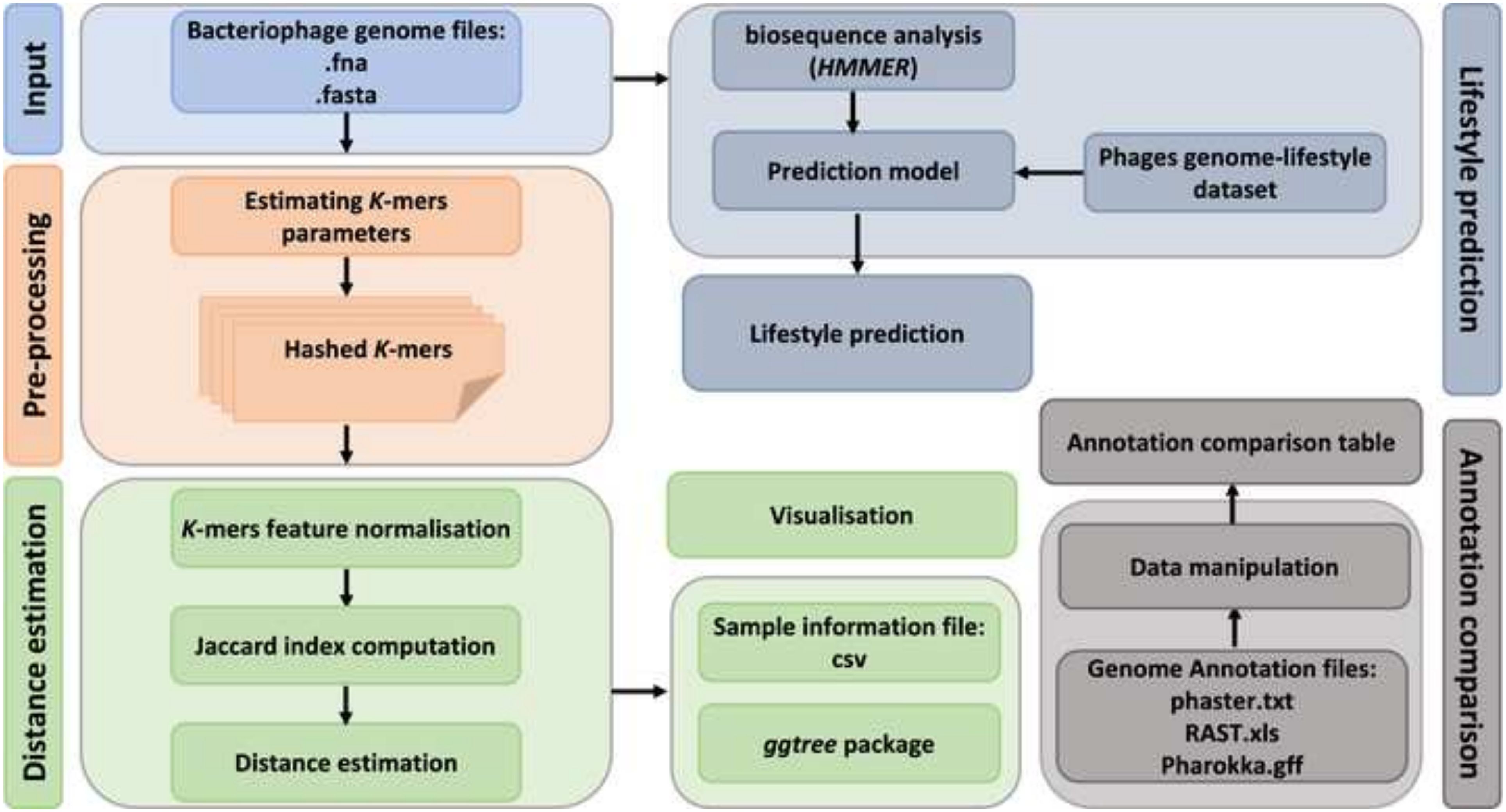

Figure 2

Click here to access/download;Figure;Figure 2.jpg

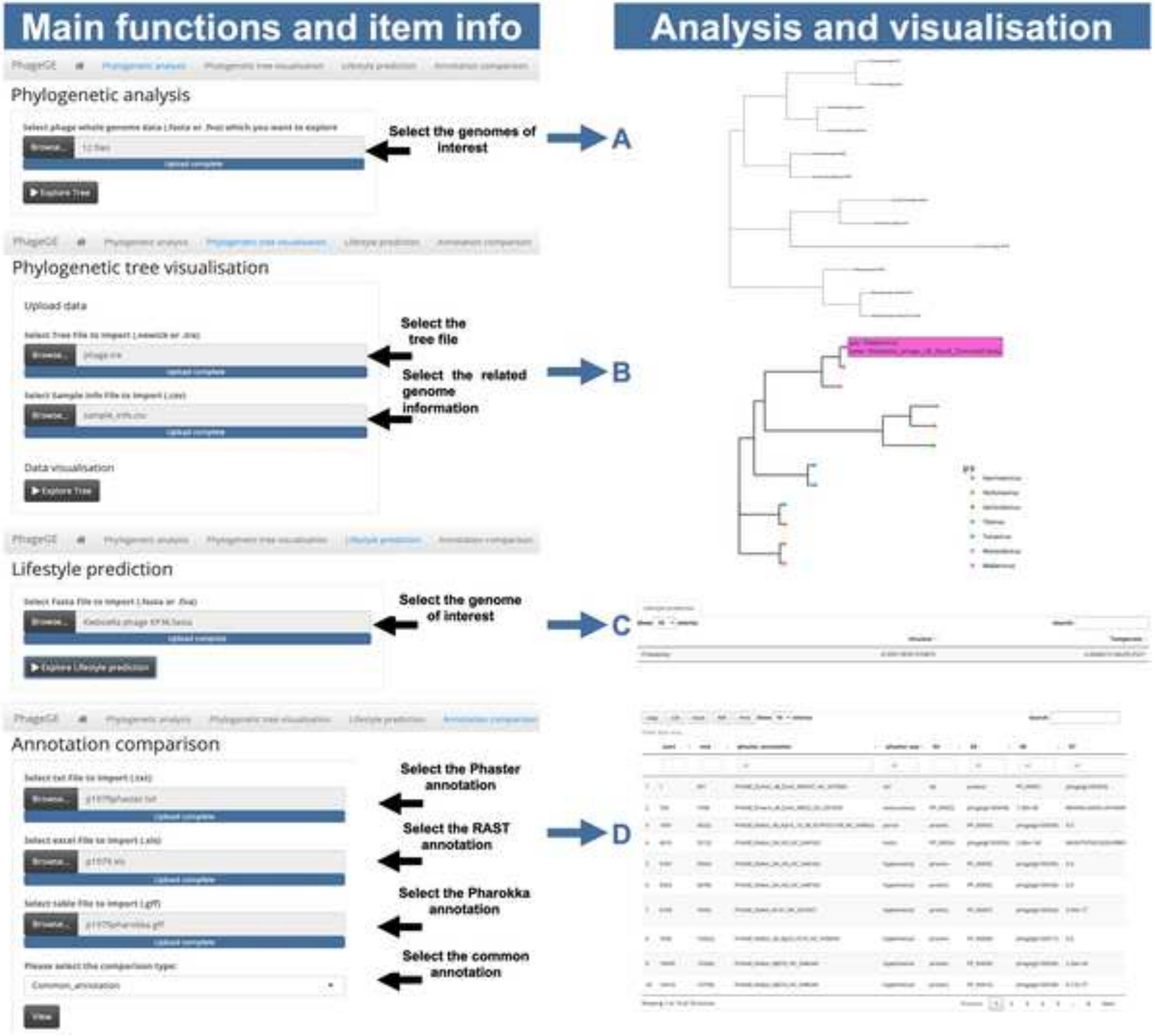

Figure 3

Click here to access/download;Figure;Figure 3.jpg

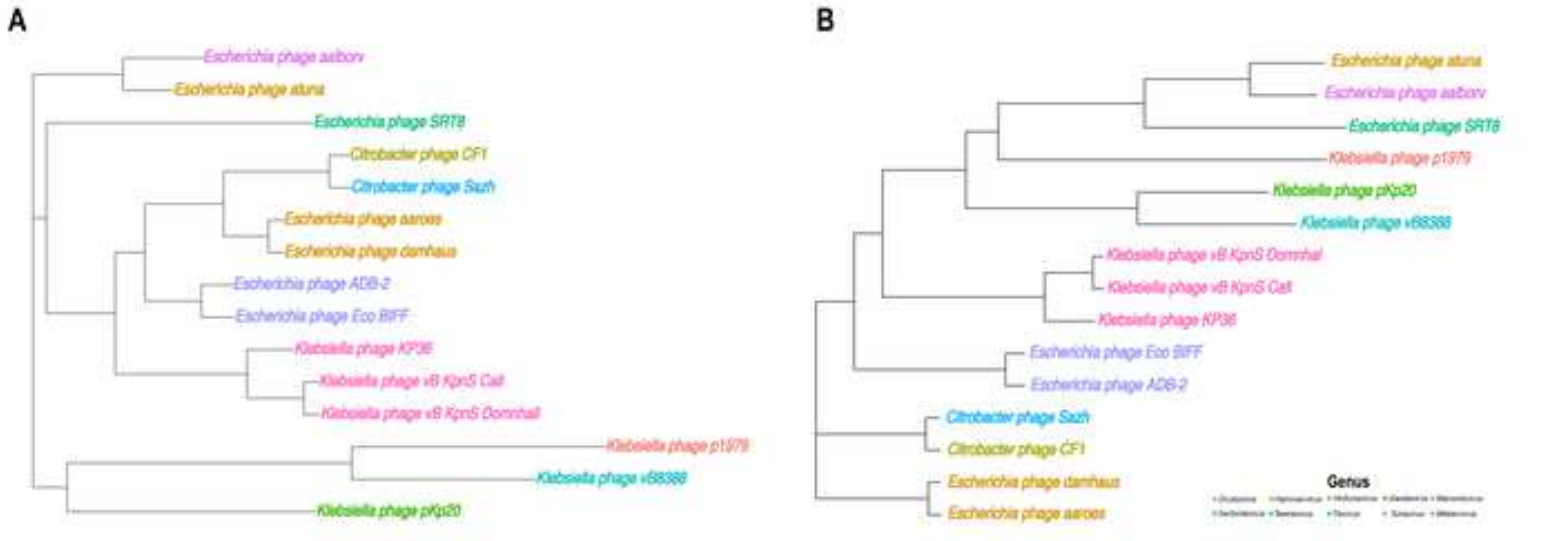

Figure 4

Click here to access/download;Figure;Figure 4.png

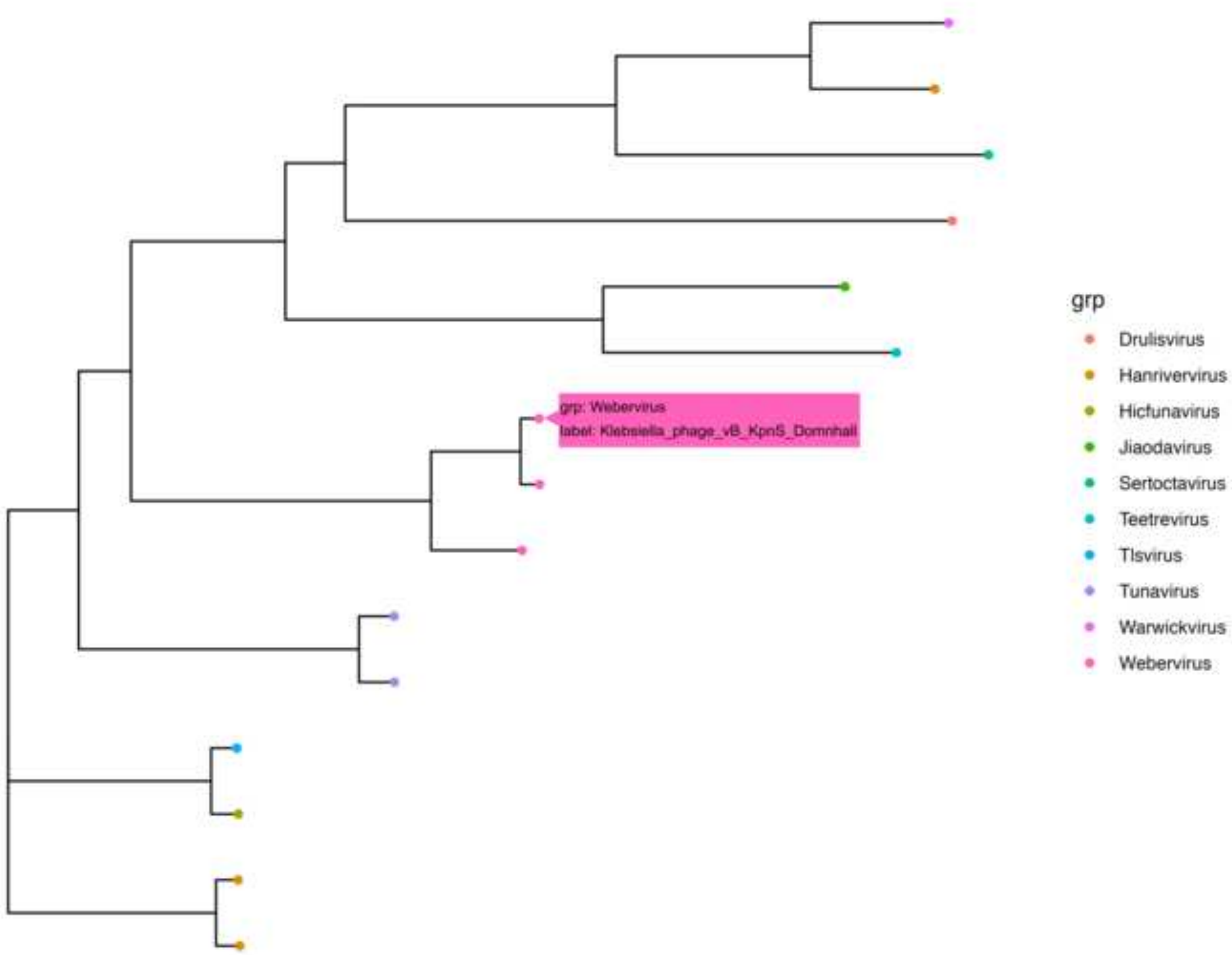

Figure 5

Click here to access/download;Figure;Figure 5.jpeg

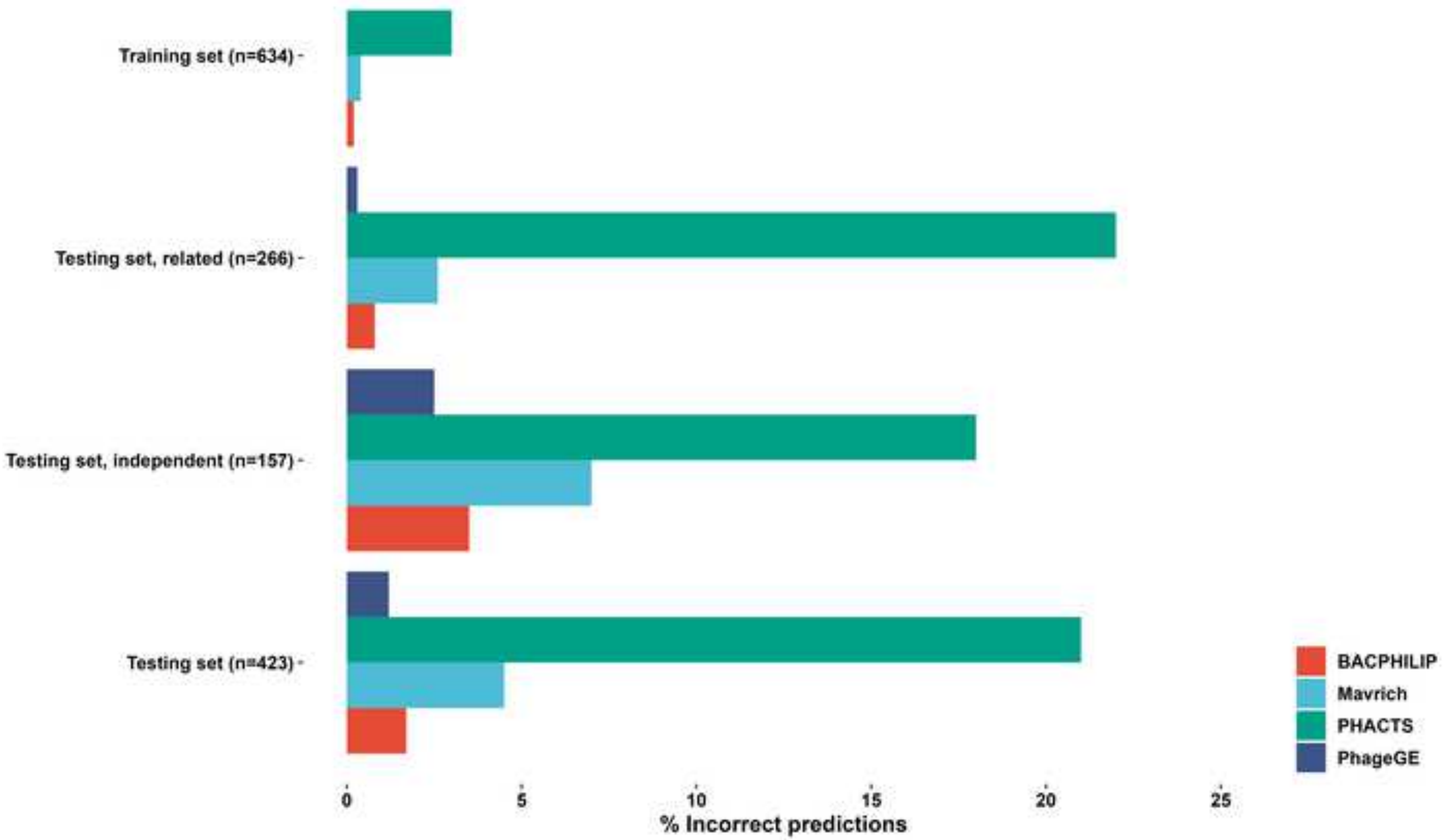

Personal Cover
Click here to access/download;Personal Cover;GIGA-D-24-00040R1 Response to reviewer.docx

MONASH University

Professor Jian Li
Fellow of the American Academy of Microbiology
Head, Laboratory of Antimicrobial Systems Pharmacology
Monash Biomedicine Discovery Institute

Dr Scott Edmunds

Editor-in-Chief

GigaScience

August 19th, 2024

Re: Manuscript GIGA-D-24-00040R1

Dear Dr Edmunds,

Thank you for providing the reviewers' comments on our manuscript "*PhageGE: An interactive web platform for exploratory analysis and visualisation of bacteriophage genomes*" and for the opportunity to revise it. Please find below our point-by-point responses to the editor and reviewers' comments. For your convenience, all major changes have been highlighted in yellow. Line numbers mentioned in our responses below refer to the marked-up version of the manuscript.

Thank you and we are looking forward to your final decision.

Yours sincerely,

Jian Li PhD

19 Innovation Walk
Monash University
VIC 3800, Australia
Telephone: (+61 3) 990 39702 Facsimile: (+61 3) 9905 6450 Email: Jian.Li@monash.edu
Web: https://research.monash.edu/en/persons/jian-li

Unintended recipient: please notify as soon as possible and destroy all pages received

MONASH University

Professor Jian Li
Fellow of the American Academy of Microbiology
Head, Laboratory of Antimicrobial Systems Pharmacology
Monash Biomedicine Discovery Institute

Point-by-point responses

Reviewers' comments:

Reviewer1:

1. I think it's important to state upfront in the abstract what phageGE can do (i.e., phylogenetic analysis, visualisation of phylo tree, predict phage lifecycle, and comparative analysis of phage genome annotations).

Response: We thank the reviewer for the suggestion. We have revised the abstract accordingly (lines 49-59).

2. Please include a link to the phageGE webpage in the abstract.

Response: We thank the reviewer for the suggestion and have provided the webpage in the abstract (line 59).

3. Spelling mistakes throughout the manuscript - as an example L288: comprehensive

Response: We thank the reviewer for the suggestion and have carefully checked our manuscript (line 228).

4. .fna and .fasta are the same format. i.e., fna is a type of FASTA file. fna specifies that the fasta file specifically contains nucleotides (instead of amino acids).

Response: We thank the reviewer for the suggestion and have revised our manuscript accordingly (lines 122, 590).

5. change "biologist friendly" to "User friendly" - this is more a philosophical/psychological change. I think bioinformatics on the command-line should be accessible and something biologists can develop. I think calling GUI programs "biologist-friendly" will deter more biologists from learning bioinformatics.

Response: We thank the reviewer for the suggestion. We have revised our manuscript accordingly, with changes made on lines 49, 89, 97, 219, and 261.

19 Innovation Walk
Monash University
VIC 3800, Australia
Telephone: (+61 3) 990 39702 Facsimile: (+61 3) 9905 6450 Email: Jian.Li@monash.edu
Web: https://research.monash.edu/en/persons/jian-li

Unintended recipient: please notify as soon as possible and destroy all pages received
