# Supplementary material for: PhageGE: an interactive web platform for exploratory analysis and visualization of bacteriophage genomes
Source: Gigascience. 2024 Sep 25;13:giae074. doi: 10.1093/gigascience/giae074 (PMC11423353; doi:10.1093/gigascience/giae074)
Supplement: giae074_Response_to_Reviewer_Comments_Original_Submission [file giae074_response_to_reviewer_comments_original_submission.pdf]

Author's Response To Reviewer Comments

Point-by-point responses

Editor comments:
1. Please register any new software application in the bio.tools and SciCrunch.org databases to receive RRID (Research Resource Identification Initiative ID) and biotoolsID identifiers, and include these in your manuscript. Computational workflows should be registered in workflowhub.eu and the DOIs cited in the relevant places in the manuscript. These will facilitate tracking, reproducibility and re-use of your tool.
Response: We have registered our application in bio.tools and SciCrunch.org databases and included the biotoolsID (biotools:phagege) and RRID (SCR_025380) in the revised manuscript (line 101).

Reviewers' comments:
Reviewer1:
The authors report here a new web-based tool called Phage Genome Explorer (PhageGE) for the interactive analysis of phage genomic data, which facilitates phylogenetic analysis and visualisation, the prediction of lytic vs., lysogenic lifestyles, and the interrogation of data generated by genome annotation tools (e.g., Pharokka). I commend the authors for developing this user-friendly tool that allows for greater access to non-experts. I believe this tool will have utility across clinical research and basic phage biology. I've tested the tool using both author supplied test data and data I've generated, and I have no major comments about the results and usability of PhageGE. However, I believe additional revisions are needed to strengthen the overall manuscript.

1. I would like to see the option to upload multi-fasta files implemented as a means to streamline usability. I think this can be implemented for both "phylogenetic analysis" and "lifestyle prediction" sections.
Response: We thank the reviewer for the suggestion and especially for providing the code for implementing multi-fasta format in our tools. We have incorporated the multi-fasta format into the "Phylogenetic analysis" function and revised the related description in the manuscript (lines 128-130). We have updated the previous "Lifestyle prediction" function for predicting multiple phage genomes simultaneously.

2. How does PhageGE scale to large metagenomic datasets? Unfortunately, I was unable to test this without the multi-fasta input option. However, I think it could scale nicely, especially with a circular tree format.
Response: We thank the reviewer for the suggestion. We have updated phageGE with a multi-fasta format input option and also provided an option for the final tree format (e.g., rectangular and circular format) (lines 128-132). We would like to clarify that the primary aim of PhageGE is to analyse phage genomic data, assuming that users already have assembled phage genomes rather than detecting them directly from large metagenomic datasets. This focus allows us to provide a robust and efficient tool specifically tailored for phage genome analysis. We apologise for any confusion this may have caused. The detection of phage sequences directly from large metagenomic datasets is beyond the current scope of PhageGE. Nevertheless, we acknowledge its importance and will consider developing this functionality in the next version of PhageGE.

3. Viral clusters have been shown to be important in determining viral diversity, and I think it would be a useful addition to the phylogenetic-based analyses. c.f., Camarillo-Guerrero et al., 2021. PMID: 33606979 and rBlast https://github.com/mhahsler/rBLAST
Response: We agree that viral clusters play a crucial role in determining viral diversity, as highlighted by Camarillo-Guerrero et al., and we appreciate the reference to rBlast as a valuable tool in this context. However, the primary aim of PhageGE is to serve as a user-friendly web tool for rapid phylogenetic analysis and lifestyle prediction, particularly catering to users with limited programming experience. Additionally, PhageGE is designed to accelerate the translation of phage therapy into the clinic by providing phage phylogenetic and lifestyle information. As such, we have focused on providing an accessible and efficient platform for these specific purposes. While the inclusion of viral cluster analysis is beyond the current scope of PhageGE, we recognise its importance and potential benefits and will consider incorporating this feature in the next version of PhageGE.

4. On the "Phylogenetic analysis" landing page, I think "select phage whole genome data" should read "select phage genome data" as whole genome data would imply that phage particles were isolated and sequenced.
Response: We apologise for any confusion caused by the terminology on the "Phylogenetic analysis" landing page. We understand that "whole genome data" implies that phages were isolated and sequenced. To clarify, the primary function of PhageGE is to analyse assembled phage genomic data, which should use “phage whole-genome data” in the landing page as well as the usage description. To prevent any further misunderstanding, we have updated the description for PhageGE: "To demonstrate the functions and the scope of application of PhageGE, we herein describe the results of a case study using PhageGE, including phage whole-genome data (i.e., .fasta), a phylogenetic tree file (i.e., .tre), and genome annotation data (i.e., .xls, .txt and .gff), collectively referred to as “Example Data” (Figure 1)." (lines 105-108).

5. "This demonstrates that the phylogenetic analysis performance of PhageGE is accurate and comparable to the multiple sequence alignment-based approach." And "It has demonstrated the ability to accurately reconstruct biologically relevant phylogenies with thousands of microbial genomes [40-42]. The description of this function is briefly outlined below." How do phylogenies obtained using whole phage genomes (k-mer, ANI, or otherwise) compare to those reconstructed using the large terminase gene?
Response: We thank the reviewer for the insightful question regarding the comparison between phylogenies obtained from PhageGE and those reconstructed using the large terminase gene. Although both phylogeny analyses from whole phage genomes (k-mer based) and the large terminase gene can provide insights into phage diversity and evolution, there is a distinction. Whole-genome based analysis utilises the entire genomic content, capturing the full extent of genetic variation across the genome; while phylogeny reconstructed using a single gene (i.e. the large terminase gene) provides a narrower view of the phage's evolutionary history and potentially misses some genetic variations present. Furthermore, phages have the capability to lose or duplicate genes, including the large terminase gene, potentially leading to inaccuracies in phylogenetic inference (Nat. Microbiol., 2017, 2(9), 1-9; Nat. Rev. Microbiol., 2021, 15(3), 161-168). In contrast, k-mer based whole-genome phylogenies offer a comprehensive and high-resolution view of phage relationships, particularly valuable in distinguishing closely related phages and providing a more holistic view of their evolutionary relationships (mBio, 2017, 8(4), 10-1128). Therefore, we integrated a k-mer based whole phage genome phylogenetic analysis function into PhageGE to provide a high-resolution view of phage phylogeny for clinical translation.

6. "Furthermore, combining whole-genome sequencing (WGS) with in silico prediction enables rapid prediction of phage lifestyle [18]. Several popular bioinformatic pipelines and tools are available for such analyses, including MAFFT, RAxML and IQ-TREE (for multiple sequence alignment and phylogenetic analysis) [19-21], ggtree (for the visualisation of phylogeny data) [22], PHACTS and BACPHLIP (for phage lifestyle prediction) [18, 23]." What do each of the programs do? Perhaps restructure writing to reflect programs at higher-order groups. e.g., Several popular bioinformatic pipelines and tools are available for multiple sequence alignment (MAFFT), phylogenetic reconstruction (RAxML, IQ-TREE), visualisation of phylogeny (ggtree), and for phage lifestyle prediction (PHACTS, BACPHLIP).
Response: We thank the reviewer for the suggestion. The sentence has been restructured accordingly (lines 85-91).

7. "However, utilising these tools requires proficient programming skills, therefore, a biologist-friendly pipeline for phage genomic analyses is urgently needed to address the aforementioned limitations in phage genomic analysis." Its not entirely clear what the aforementioned limitations are. Are you referring to: "Optimising phage therapy in patients requires key pharmacological information, including infection cycle, gene content and phage taxonomy"
Response: The limitations refer to proficient programming skills required for phage genomic analysis when using these tools. We have clarified this point in the revised manuscript (lines 88-91).

General editorial revisions are required, some examples are given below:
Response: We thank the reviewer for the suggestions. In addition to the general editorial revisions suggested by the reviewer below, we have substantially revised the manuscript to improve grammar. Minor changes were not highlighted.

8. "To demonstrate the functions and application scope of PhageGE"
To demonstrate the functions and the scope of application of PhageGE
Response: The sentence has been revised accordingly (line 105).

9. "This demonstrates that the phylogenetic analysis performance of PhageGE is accurate and comparable to the multiple sequence alignment-based approach."
This demonstrates that the performance of the phylogenetic analysis of PhageGE is accurate and comparable to the multiple sequence alignment-based approach.
Response: The sentence has been revised accordingly (lines 142-144).

10. "Respectively" is used too frequently and creates confusing sentence constructions.
e.g., "By selecting "common_annotation", a table with 75, 45, 51 genes that were annotated in all three pipelines were generated for KP36, vB8838 and FK1979, respectively. We also identified 17, 7 and 12 unique genes, respectively, from the Pharokka pipeline by selecting "Pharokka_only" option."
Response: We thank the reviewer for the suggestion. The second sentence above has been rewritten (lines 194-195).

11. "By employing an improved searching function (i.e. searching a sequence file against the build-in HMM [Hidden Markov Model] database)"
By employing an improved search function (i.e. searching a sequence file against the built-in HMM [Hidden Markov Model] database)"
Response: The manuscript has been revised accordingly (line 323).

12. "To illustrate the phylogenetic analysis function in PhageGE, we employed our GitHub example dataset which consists of 14 phage genomes (Citrobacter, Escherichia, and Klebsiella) from 9 different genera (Figure 2A)."
Need to make clear what the link between the 14 phage genomes to Citrobacter, Escherichia, and Klebsiella are. Are they 14 genomes of lytic phages that target Citrobacter, Escherichia, and Klebsiella? Or are they 14 phage sequences/genomes detected from bacterial isolate genomes of Citrobacter, Escherichia, and Klebsiella? I think a section describing the origin of data used would be helpful for readers.
Response: We thank the reviewer for the suggestion and have revised the manuscript accordingly (lines 112-121). All 15 phages are lytic phages that target Citrobacter freundiifreundii (2 phages), Escherichia coli (7 phages), and Klebsiella pneumoniae (6 phages).
These 15 phage genomes were selected to demonstrate the application of PhageGE to a wide range of phages targeting clinically relevant pathogens. We included a K. pneumoniae phage, pKp20, and performed the phylogenetic analysis for this phage along with the other 14 phages. Notably, the taxonomic and lifestyle results of pKp20 contributed to a recent successful clinical case (Antimicrob. Agents Chemother., 2023, 67(4), e00037-23).

13. "To compare the results obtained from PhageGE with the multiple sequence alignment-based approach, we also conducted a multiple sequence alignment-based phylogenetic analysis using MAFFT v7.47 alongside the phylogenetic analysis conducted in PhageGE"
What is the first MSA-based approached referring to here? I think the results section requires a brief overview of the steps executed within PhageGE to orientate the readers. This would provide a baseline understanding in an effort to facilitate the comparative narrative.
Response: We have revised the manuscript to clarify this point (lines 126-133). The MSA-based approach here refers to the phylogenetic analysis using MAFFT v7.47 and fasttree v2.1.10. We have also included a brief discussion on the performance of PhageGE in phylogenetic analysis with uploaded phage genomes.

14. "Its aim is to provide an interactive visualisation platform that improves the reusability of phylogenetic data and facilitates the phylogenetic analysis of phage comparative genomics studies." Reusability = reproducibility?
Response: This sentence has been changed to “...interactive visualisation platform that enhances the accessibility of phylogenetic data...” (line 147).

15. "Overall, all four functions from PhageGE serve as a guide for the exploration of phage genomic features and will expedite the clinical translation of phage therapy."

The test data set requires more phage genomes that serve as positive and negative controls, including eukaryotic viruses. Table 2 phage lifecycle prediction needs controls for temperate phages, and non-phage viruses.
Response: We thank the reviewer for the suggestion and have included more phages (e.g. temperate phages) in the lifestyle prediction table (Table 2) to serve as positive (e.g. KP36 and pkp20) and negative (e.g. NC_017985 and NC_027339) controls (lines 176-180). Regarding the inclusion of eukaryotic viruses, PhageGE is for genomic analyses of phages specifically, not non-phage viruses. We have also updated our current function to pop up an error message when non-phage viruses are detected: "The input is not from phage viruses".

16. Figure legends require more descriptive text in order to assess.
Response: We thank the reviewer for the suggestion and have improved the figure legends accordingly.

17. Image quality of figures needs improvement, especially figure 5.
Response: All figures have been updated with a resolution of 300 dpi or higher.

18. Last sentence of first paragraph - upton = upon; Second paragraph - multi-omics has* the
Response: We apologise for the typographic errors and the manuscript has been revised accordingly (lines 78 and 80).

Reviewer2:
Major points:
1. It was seen that various annotation tools have been developed for phage genomes, and there are several works developed as integrated tools or pipelines for phage genome annotation and visualization. For example, Prophage Hunter (Song et al. 2019), Galaxy and Apollo (Ramsey et al. 2020), PhaGAA (Wu et al. 2023), … et al. However, the authors did not mention and discuss those works. Compared with those published works, PhageGE was designed with its functions some different from them, but still limited for the research community.
Response: We thank the reviewer for the comments regarding the comparison of PhageGE with other phage genome annotation and visualisation tools. In the revised manuscript we have clarified that PhageGE serves as a biologist-friendly interactive platform for phage genome analysis with a particular emphasis on phylogeny, lifestyle prediction, interactive phylogenetic tree visualisation, and annotation comparison (lines 92-98). The interactive visualisation capabilities of PhageGE are tailored to improve the accessibility and usability of phylogenetic data, facilitating comparative genomics studies and clinical translation within the phage research community.
Prophage Hunter is for studying active phages from whole genome assemblies of bacteria. The functionalities of PhageGE are designed to complement, rather than replicate, the capabilities of tools like Prophage Hunter.
The main annotation pipeline used in Galaxy and Apollo is PHANOTATE, which has been adapted into the Pharokka pipeline (Bioinformatics, 2023, 39(1), p.btac776). PhageGE focuses on integrating annotations into an interactive environment for comparative genome analysis and visualisation. Our approach enhances the utility of the annotations by providing a platform for deeper exploration and interpretation of phylogenetic relationships.
PhaGAA is an excellent online integrated platform for phage genome annotation and analysis, focusing on DNA/protein-based annotation, host prediction, and lifestyle reorganisation. The lifestyle reorganisation method in PhaGAA directly integrates PhaTYP (Brief. Bioinform., 2023, 24(1), p.bbac487). The primary utility of PhaTYP is analysing phage lifestyle in human neonates' gut data, showcasing its value in studying phages in metagenomic contexts and enhancing our understanding of microbial communities.
In summary, PhageGE offers unique functionalities that complement existing tools, focusing on providing a biologist-friendly and specialised environment for phage genome analysis.

2. As pointed out above, PhageGE's functions were not comprehensive enough, especially did not address the characteristics of the host of bacteriophage or phage-host interaction which are important for phage genome studies. In addition, currently a tool like PhageGE would be expected to analyze metagenomic data with a large of short reads. Moreover, identification of resistance genes, analyzing potentially encoded resistance genes within the phage genome is crucial in phage genome analysis. So, adding analysis function of antibiotic resistance gene dissemination, examining genes related to antibiotic resistance in the

phage genome, especially those that might affect host bacterial resistance through horizontal gene transfer, could greatly enhance the understanding of bacteriophages, their evolution, and host interactions if these analytical functions were integrated into the PhageGE pipeline.
Response: We appreciate the reviewer's valuable suggestions for enhancing PhageGE. We agree that understanding host characteristics and phage-host interactions are crucial; however, they are beyond the current scope of PhageGE. As mentioned in our response to Comment #1 above, PhageGE focuses on phylogenetic analysis and lifestyle prediction, aiming to expedite clinical translation of phage therapy (lines 116-121 and 176-177). This focus has led to a successful clinical case study (Antimicrob. Agents Chemother., 2023, 67(4), e00037-23).
Regarding antibiotic resistance gene (ARG) analysis, we recognise its critical role in understanding phage biology and their potential impact on bacterial resistance through horizontal gene transfer. Notably, recent studies have demonstrated that phages and prophages rarely carry ARGs, and bona fide ARGs attributed to phages in human- or mouse-associated viromes were previously overestimated due to bacterial DNA contamination and relaxed detection thresholds, leading to high false-positive rates (ISME, 2017, 11(1), 237-247; ISME Commun., 2021, 1(1), 55). Nonetheless, we will consider incorporating this function in future versions of PhageGE.

3. As a presentation of an application, the authors provided limited cases with example datasets, and limited analysis.
Response: We thank the reviewer for the suggestion. In the revised manuscript we have included more example datasets to demonstrate each function (e.g., phylogenetic analysis and lifestyle prediction) (lines 112-121, 137-144, and 176-180). Moreover, we have demonstrated the application of functions from PhageGE using a clinical case study (lines 116-121 and 177-180).

Minor points:
4. The authors highlight in the background section the role of phage genome analysis in developing phage therapies. Therefore, it would be beneficial to demonstrate the application of this tool in case studies.
Response: We thank the reviewer for the suggestion. The manuscript has been revised to include a clinical case study (Antimicrob. Agents Chemother., 2023, 67(4), e00037-23) which demonstrates the application of phageGE (lines 112-121 and 176-180). This case study involved a recurrent urinary tract infection, and both taxonomy information from phylogeny analysis and the lifestyle prediction had played key roles in the phage selection.

5. While many offline tools for constructing phage evolutionary trees have been developed, a major disadvantage of a web tool is its lengthy runtime. The capacity of the tool to process a significant number of sequence data and the need for a runtime comparison should be addressed.
Response: We thank the reviewer for the suggestion. In the revised version we have included a comparison of the PhageGE runtime with the MSA-based approach (lines 138-144). On a 2-GHz CPU with 64 GB RAM, PhageGE performed phylogenetic analysis for 15 and 146 phage genomes in 0.22 minutes and 4.42 minutes, respectively. In comparison, the MAS-based approach required more than 30 minutes and 296 minutes accordingly. Therefore, PhageGE offers superior computational and analysis efficiency.

6. The image resolution is too low, at only 144 dpi, insufficient for the required 300 dpi. Many characters in Figure 2A are unclear, suggesting a need for improved resolution.
Response: As per Reviewer 1, Point 17, all figures have been updated with a resolution of 300 dpi or higher.

7. The website http://phagege.com/ is not functioning and cannot be accessed.
Response: We have retested our current version and the url works properly.
