# Supplementary material for: PhageGE: an interactive web platform for exploratory analysis and visualization of bacteriophage genomes
Source: Gigascience. 2024 Sep 25;13:giae074. doi: 10.1093/gigascience/giae074 (PMC11423353; doi:10.1093/gigascience/giae074)
Supplement: giae074_Response_to_Reviewer_Comments_Revision_1 [file giae074_response_to_reviewer_comments_revision_1.pdf]

Author's Response To Reviewer Comments

Reviewers’ comments:
Reviewer1:
1. I think it's important to state upfront in the abstract what phageGE can do (i.e., phylogenetic analysis, visualisation of phylo tree, predict phage lifecycle, and comparative analysis of phage genome annotations).
Response: We thank the reviewer for the suggestion. We have revised the abstract accordingly (lines 49-59).
2. Please include a link to the phageGE webpage in the abstract.
Response: We thank the reviewer for the suggestion and have provided the webpage in the abstract (line 59).
3. Spelling mistakes throughout the manuscript - as an example L288: comprehensive
Response: We thank the reviewer for the suggestion and have carefully checked our manuscript (line 228).
4. .fna and .fasta are the same format. i.e., fna is a type of FASTA file. fna specifies that the fasta file specifically contains nucleotides (instead of amino acids).
Response: We thank the reviewer for the suggestion and have revised our manuscript accordingly (lines 122, 590).
5. change "biologist friendly" to "User friendly" - this is more a philosophical/psychological change. I think bioinformatics on the command-line should be accessible and something biologists can develop. I think calling GUI programs "biologist-friendly" will deter more biologists from learning bioinformatics.
Response: We thank the reviewer for the suggestion. We have revised our manuscript accordingly, with changes made on lines 49, 89, 97, 219, and 261.
