# Supplementary material for: PhageGE: an interactive web platform for exploratory analysis and visualization of bacteriophage genomes
Source: Gigascience. 2024 Sep 25;13:giae074. doi: 10.1093/gigascience/giae074 (PMC11423353; doi:10.1093/gigascience/giae074)
Supplement: giae074_Response_to_Reviewer_Comments_Revision_2 [file giae074_response_to_reviewer_comments_revision_2.pdf]

Author's Response To Reviewer Comments

Editors' comments:
1. Please cite GitHub URL in your References and add the Refence Number. You will need to renumber all your references in the main text as well.
Response: We thank the editor for the suggestion. We have cited our GitHub URL in the references with updating the order in the main text (lines 110, 373 and 384, and reference 24).
2. Please register PhageGE in scicrunch.org and add the RRID number here. Also list the RRID under Source Code Availability below.
Response: We thank the editor for the suggestion and have provided the RRID number (SCR_025380) in the manuscript (lines 271and 377).
