# Supplementary material for: PhageGE: an interactive web platform for exploratory analysis and visualization of bacteriophage genomes
Source: Gigascience. 2024 Sep 25;13:giae074. doi: 10.1093/gigascience/giae074 (PMC11423353; doi:10.1093/gigascience/giae074)
Supplement: giae074_Reviewer_1_Report_Original_Submission — Andre Mu -- 2/18/2024 Reviewed [file giae074_reviewer_1_report_original_submission.pdf]

Reviewer Report

Title: PhageGE: An interactive web platform for exploratory analysis and visualisation of bacteriophage genomes

Version: Original Submission **Date:** 2/18/2024

Reviewer name: Andre Mu

Reviewer Comments to Author:

PhageGE: An interactive web platform for exploratory analysis and visualisation of bacteriophage genomesZhao et al.The authors report here a new web-based tool called Phage Genome Explorer (PhageGE) for the interactive analysis of phage genomic data, which facilitates phylogenetic analysis and visualisation, the prediction of lytic vs., lysogenic lifestyles, and the interrogation of data generated by genome annotation tools (e.g., Pharokka). I commend the authors for developing this user-friendly tool that allows for greater access to non-experts. I believe this tool will have utility across clinical research and basic phage biology.I've tested the tool using both author supplied test data and data I've generated, and I have no major comments about the results and usability of PhageGE. However, I believe additional revisions are needed to strengthen the overall manuscript.1) I would like to see the option to upload multi-fasta files implemented as a means to streamline usability. I think this can be implemented for both "phylogenetic analysis" and "lifestyle prediction" sections. The code below may work:# Read the multi-fasta filefasta <- readLines("input_multifasta.fna")# Initialize variablesOUT <- NULLcurrent_sequence <- NULL# Process each line in the multi-fasta filefor (line in fasta) {if (grepl("^>", line)) {# If line starts with ">" then it is a headerheader <- gsub("[^a-zA-Z0-9]+", "_", substr(line, 2, nchar(line)), perl = TRUE)OUT <- paste0(header, ".fna")write("", file = OUT) # Create an empty output file} else {# Otherwise, the line is sequence dataif (is.null(OUT)) {stop("Error: No header found before sequence data.")}write(line, file = OUT, append = TRUE) # Append sequence data to output file}}2) How does PhageGE scale to large metagenomic datasets? Unfortunately, I was unable to test this without the multi-fasta input option. However, I think it could scale nicely, especially with a circular tree format.3) Viral clusters have been shown to be important in determining viral diversity, and I think it would be a useful addition to the phylogenetic-based analyses.c.f., Camarillo-Guerrero et al., 2021. PMID: 33606979 andrBlast https://github.com/mhahsler/rBLAST4) On the "Phylogenetic analysis" landing page, I think "select phage whole genome data" should read "select phage genome data" as whole genome data would imply that phage particles were isolated and sequenced.The following comments are with regards to the body of the manuscript."This demonstrates that the phylogenetic analysis performance of PhageGE is accurate and comparable to the multiple sequence alignment-based approach."And"It has demonstrated the ability to accurately reconstruct biologically relevant phylogenies with thousands of microbial genomes [40-42]. The description of this function is briefly outlined below."How do phylogenies obtained using whole phage genomes (k-mer, ANI, or otherwise) compare to those reconstructed using the large terminase gene?"Furthermore, combining whole-genome sequencing (WGS) with in silico prediction enables rapid prediction of phage life style [18]. Several popular bioinformatic pipelines and tools are available for such analyses, including MAFFT, RAxML and IQ-TREE

(for multiple sequence alignment and phylogenetic analysis) [19-21], ggtree (for the visualisation of phylogeny data) [22], PHACTS and BACPHLIP (for phage lifestyle prediction) [18, 23]."What do each of the programs do? Perhaps restructure writing to reflect programs at higher-order groups.e.g., Several popular bioinformatic pipelines and tools are available for multiple sequence alignment (MAFFT), phylogenetic reconstruction (RAxML, IQ-TREE), visualisation of phylogeny (ggtree), and for phage lifestyle prediction (PHACTS, BACPHLIP)."However, utilising these tools requires proficient programming skills, therefore, a biologist-friendly pipeline for phage genomic analyses is urgently needed to address the aforementioned limitations in phage genomic analysis."Its not entirely clear what the aforementioned limitations are. Are you referring to:"Optimising phage therapy in patients requires key pharmacological information, including infection cycle, gene content and phage taxonomy"General editorial revisions are required, some examples are given below:"To demonstrate the functions and application scope of PhageGE"To demonstrate the functions and the scope of application of PhageGE"This demonstrates that the phylogenetic analysis performance of PhageGE is accurate and comparable to the multiple sequence alignment-based approach."This demonstrates that the performance of the phylogenetic analysis of PhageGE is accurate and comparable to the multiple sequence alignment-based approach."Respectively" is used too frequently and creates confusing sentence constructions.e.g.,"By selecting "common_annotation", a table with 75, 45, 51 genes that were annotated in all three pipelines were generated for KP36, vB8838 and FK1979, respectively. We also identified 17, 7 and 12 unique genes, respectively, from the Pharokka pipeline by selecting "Pharokka_only" option.""By employing an improved searching function (i.e. searching a sequence file against the build-in HMM [Hidden Markov Model] database)"By employing an improved search function (i.e. searching a sequence file against the built-in HMM [Hidden Markov Model] database)""To illustrate the phylogenetic analysis function in PhageGE, we employed our GitHub example dataset which consists of 14 phage genomes (Citrobacter, Escherichia, and Klebsiella) from 9 different genera (Figure 2A)."Need to make clear what the link between the 14 phage genomes to Citrobacter, Escherichia, and Klebsiella are. Are they 14 genomes of lytic phages that target Citrobacter, Escherichia, and Klebsiella? Or are they 14 phage sequences/genomes detected from bacterial isolate genomes of Citrobacter, Escherichia, and Klebsiella? I think a section describing the origin of data used would be helpful for readers."To compare the results obtained from PhageGE with the multiple sequence alignment-based approach, we also conducted a multiple sequence alignment-based phylogenetic analysis using MAFFT v7.47 alongside the phylogenetic analysis conducted in PhageGE"What is the first MSA-based approached referring to here? I think the results section requires a brief overview of the steps executed within PhageGE to orientate the readers. This would provide a baseline understanding in an effort to facilitate the comparative narrative."Its aim is to provide an interactive visualisation platform that improves the reusability of phylogenetic data and facilitates the phylogenetic analysis of phage comparative genomics studies."Reusability = reproducibility?"Overall, all fours functions from PhageGE serve as a guide for the exploration of phage genomic features and will expedite the clinical translation of phage therapy."The test data set requires more phage genomes that serve as positive and negative controls, including eukaryotic viruses. Table 2 phage lifecycle prediction needs controls for temperate phages, and non-phage viruses.Figure legends require more descriptive text in order to assess.Image quality of figures needs improvement, especially figure 5.Last sentence of first paragraph - upton = uponSecond paragraph - multi-omics has* the

Level of Interest

Please indicate how interesting you found the manuscript: Choose an item.

Quality of Written English

Please indicate the quality of language in the manuscript: Choose an item.

Declaration of Competing Interests

Please complete a declaration of competing interests, considering the following questions:

- Have you in the past five years received reimbursements, fees, funding, or salary from an organisation that may in any way gain or lose financially from the publication of this manuscript, either now or in the future?
- Do you hold any stocks or shares in an organisation that may in any way gain or lose financially from the publication of this manuscript, either now or in the future?
- Do you hold or are you currently applying for any patents relating to the content of the manuscript?
- Have you received reimbursements, fees, funding, or salary from an organization that holds or has applied for patents relating to the content of the manuscript?
- Do you have any other financial competing interests?
- Do you have any non-financial competing interests in relation to this paper?

If you can answer no to all of the above, write 'I declare that I have no competing interests' below. If your reply is yes to any, please give details below.

I declare that I have no competing interests

I agree to the open peer review policy of the journal. I understand that my name will be included on my report to the authors and, if the manuscript is accepted for publication, my named report including any attachments I upload will be posted on the website along with the authors' responses. I agree for my report to be made available under an Open Access Creative Commons CC-BY license (http://creativecommons.org/licenses/by/4.0/). I understand that any comments which I do not wish to be included in my named report can be included as confidential comments to the editors, which will not be published.

Choose an item.

To further support our reviewers, we have joined with Publons, where you can gain additional credit to further highlight your hard work (see: https://publons.com/journal/530/gigascience). On publication of

this paper, your review will be automatically added to Publons, you can then choose whether or not to claim your Publons credit. I understand this statement.

Yes Choose an item.
