# Supplementary material for: PhageGE: an interactive web platform for exploratory analysis and visualization of bacteriophage genomes
Source: Gigascience. 2024 Sep 25;13:giae074. doi: 10.1093/gigascience/giae074 (PMC11423353; doi:10.1093/gigascience/giae074)
Supplement: giae074_Reviewer_1_Report_Revision_1 — Andre Mu -- 7/15/2024 Reviewed [file giae074_reviewer_1_report_revision_1.pdf]

Reviewer Report

Title: PhageGE: An interactive web platform for exploratory analysis and visualisation of bacteriophage genomes

Version: Revision 1 **Date:** 7/15/2024

Reviewer name: Andre Mu

Reviewer Comments to Author:

I thank the authors for addressing most of the concerns from the first review; especially incorporating the multi-fasta upload function.I think it's important to state upfront in the abstract what phageGE can do (i.e., phylogenetic analysis, visualisation of phylo tree, predict phage lifecycle, and comparative analysis of phage genome annotations).Please include a link to the phageGE webpage in the abstract.Spelling mistakes throughout the manuscript - as an example L288: comprehensive.fna and .fasta are the same format. i.e., fna is a type of FASTA file. fna specifies that the fasta file specifically contains nucleotides (instead of amino acids).change "biologist friendly" to "User friendly" - this is more a philosophical/psychological change. I think bioinformatics on the command-line should be accessible and something biologists can develop. I think calling GUI programs "biologist-friendly" will deter more biologists from learning bioinformatics.

Level of Interest

Please indicate how interesting you found the manuscript: Choose an item.

Quality of Written English

Please indicate the quality of language in the manuscript: Choose an item.

Declaration of Competing Interests

Please complete a declaration of competing interests, considering the following questions:

- Have you in the past five years received reimbursements, fees, funding, or salary from an organisation that may in any way gain or lose financially from the publication of this manuscript, either now or in the future?
- Do you hold any stocks or shares in an organisation that may in any way gain or lose financially from the publication of this manuscript, either now or in the future?
- Do you hold or are you currently applying for any patents relating to the content of the manuscript?
- Have you received reimbursements, fees, funding, or salary from an organization that holds or has applied for patents relating to the content of the manuscript?
- Do you have any other financial competing interests?

- Do you have any non-financial competing interests in relation to this paper?

If you can answer no to all of the above, write 'I declare that I have no competing interests' below. If your reply is yes to any, please give details below.

please see confidential comments to editor

I agree to the open peer review policy of the journal. I understand that my name will be included on my report to the authors and, if the manuscript is accepted for publication, my named report including any attachments I upload will be posted on the website along with the authors' responses. I agree for my report to be made available under an Open Access Creative Commons CC-BY license (http://creativecommons.org/licenses/by/4.0/). I understand that any comments which I do not wish to be included in my named report can be included as confidential comments to the editors, which will not be published.

Choose an item.

To further support our reviewers, we have joined with Publons, where you can gain additional credit to further highlight your hard work (see: https://publons.com/journal/530/gigascience). On publication of this paper, your review will be automatically added to Publons, you can then choose whether or not to claim your Publons credit. I understand this statement.

Yes Choose an item.
