# Supplementary material for: PhageGE: an interactive web platform for exploratory analysis and visualization of bacteriophage genomes
Source: Gigascience. 2024 Sep 25;13:giae074. doi: 10.1093/gigascience/giae074 (PMC11423353; doi:10.1093/gigascience/giae074)
Supplement: giae074_Reviewer_2_Report_Original_Submission — Huaiqiu Zhu -- 4/7/2024 Reviewed [file giae074_reviewer_2_report_original_submission.pdf]

Reviewer Report

Title: PhageGE: An interactive web platform for exploratory analysis and visualisation of bacteriophage genomes

Version: Original Submission **Date:** 4/7/2024

Reviewer name: Huaiqiu Zhu

Reviewer Comments to Author:

I have read through the paper titled "PhageGE: An interactive web platform for exploratory analysis and visualisation of bacteriophage genomes" by Zhao et al., thus to understand the authors' work. In this manuscript, the authors developed a graphical interactive pipeline, named PhageGE, to implement the task of phage genome analysis based on phage whole-genome sequence data. As described, the tool PhageGE is able to perform "phage phylogenetic analysis, tree visualisation, lifestyle prediction, and manipulation of phage genome annotation datasets". Part of these functions actually deployed method previously designed by the authors themselves (annotation of lifestyle) and others are existing published tools (RAST, PHASTER, and Pharokka). However, these functions were still not comprehensive enough, especially they did not address the characteristics of the host of bacteriophage or phage-host interaction which are important for phage genome studies. Herein I would like to raise my several concerns on this work as follows.Major points:(1) It was seen that various annotation tools have been developed for phage genomes, and there are several works developed as integrated tools or pipelines for phage genome annotation and visualization. For example, Prophage Hunter (Song et al. 2019), Galaxy and Apollo (Ramsey et al. 2020), PhaGAA (Wu et al. 2023), … et al. However, the authors did not mention and discuss those works. Compared with those published works, PhageGE was designed with its functions some different from them, but still limited for the research community.(2) As pointed out above, PhageGE's functions were not comprehensive enough, especially did not address the characteristics of the host of bacteriophage or phage-host interaction which are important for phage genome studies. In addition, currently a tool like PhageGE would be expected to analyze metagenomic data with a large of short reads. Moreover, identification of resistance genes, analyzing potentially encoded resistance genes within the phage genome is crucial in phage genome analysis. So, adding analysis function of antibiotic resistance gene dissemination, examining genes related to antibiotic resistance in the phage genome, especially those that might affect host bacterial resistance through horizontal gene transfer, could greatly enhance the understanding of bacteriophages, their evolution, and host interactions if these analytical functions were integrated into the PhageGE pipeline.(3) As a presentation of an application, the authors provided limited cases with example datasets, and limited analysis.Minor points:(1) The authors highlight in the background section the role of phage genome analysis in developing phage therapies. Therefore, it would be beneficial to demonstrate the application of this tool in case studies.(2) While many offline tools for constructing phage evolutionary trees have been developed, a major disadvantage of a web tool is its lengthy runtime. The capacity of the tool to process a significant number of sequence data and the need for a runtime comparison should be addressed.(3) The image resolution is too low, at only 144 dpi, insufficient for the required 300 dpi.

Many characters in Figure 2A are unclear, suggesting a need for improved resolution.(4) The website http://phagege.com/ is not functioning and cannot be accessed.

Level of Interest

Please indicate how interesting you found the manuscript: Choose an item.

Quality of Written English

Please indicate the quality of language in the manuscript: Choose an item.

Declaration of Competing Interests

Please complete a declaration of competing interests, considering the following questions:

- Have you in the past five years received reimbursements, fees, funding, or salary from an organisation that may in any way gain or lose financially from the publication of this manuscript, either now or in the future?
- Do you hold any stocks or shares in an organisation that may in any way gain or lose financially from the publication of this manuscript, either now or in the future?
- Do you hold or are you currently applying for any patents relating to the content of the manuscript?
- Have you received reimbursements, fees, funding, or salary from an organization that holds or has applied for patents relating to the content of the manuscript?
- Do you have any other financial competing interests?
- Do you have any non-financial competing interests in relation to this paper?

If you can answer no to all of the above, write 'I declare that I have no competing interests' below. If your reply is yes to any, please give details below.

I declare that I have no competing interests.

I agree to the open peer review policy of the journal. I understand that my name will be included on my report to the authors and, if the manuscript is accepted for publication, my named report including any attachments I upload will be posted on the website along with the authors' responses. I agree for my report to be made available under an Open Access Creative Commons CC-BY license (http://creativecommons.org/licenses/by/4.0/). I understand that any comments which I do not wish to be included in my named report can be included as confidential comments to the editors, which will not be published.

Choose an item.

To further support our reviewers, we have joined with Publons, where you can gain additional credit to further highlight your hard work (see: https://publons.com/journal/530/gigascience). On publication of this paper, your review will be automatically added to Publons, you can then choose whether or not to claim your Publons credit. I understand this statement.

Yes Choose an item.
